# Dynamic Evolution of the Chloroplast Genome in the Green Algal Classes Pedinophyceae and Trebouxiophyceae

**DOI:** 10.1093/gbe/evv130

**Published:** 2015-07-01

**Authors:** Monique Turmel, Christian Otis, Claude Lemieux

**Affiliations:** Département de Biochimie, de Microbiologie et de Bio-Informatique, Institut de Biologie Intégrative et des Systèmes, Université Laval, Québec, Québec, Canada

**Keywords:** Trebouxiophyceae, Pedinophyceae, plastid genomics, genome rearrangements, inverted repeat, horizontal transfer, repeats, introns

## Abstract

Previous studies of trebouxiophycean chloroplast genomes revealed little information regarding the evolutionary dynamics of this genome because taxon sampling was too sparse and the relationships between the sampled taxa were unknown. We recently sequenced the chloroplast genomes of 27 trebouxiophycean and 2 pedinophycean green algae to resolve the relationships among the main lineages recognized for the Trebouxiophyceae. These taxa and the previously sampled members of the Pedinophyceae and Trebouxiophyceae are included in the comparative chloroplast genome analysis we report here. The 38 genomes examined display considerable variability at all levels, except gene content. Our results highlight the high propensity of the rDNA-containing large inverted repeat (IR) to vary in size, gene content and gene order as well as the repeated losses it experienced during trebouxiophycean evolution. Of the seven predicted IR losses, one event demarcates a superclade of 11 taxa representing 5 late-diverging lineages. IR expansions/contractions account not only for changes in gene content in this region but also for changes in gene order and gene duplications. Inversions also led to gene rearrangements within the IR, including the reversal or disruption of the rDNA operon in some lineages. Most of the 20 IR-less genomes are more rearranged compared with their IR-containing homologs and tend to show an accelerated rate of sequence evolution. In the IR-less superclade, several ancestral operons were disrupted, a few genes were fragmented, and a subgroup of taxa features a G+C-biased nucleotide composition. Our analyses also unveiled putative cases of gene acquisitions through horizontal transfer.

## Introduction

Chloroplasts are semiautonomous organelles that possess their own genome; with the assistance of chloroplast-targeted products encoded in the nucleus, they carry out the reactions necessary for the capture of energy from the sun as well as other functions (Gray and Archibald 2012). The chloroplasts of the photosynthetic eukaryotes belonging to the Archaeplastida or Plantae sensu lato (red algae, glaucophytes, and viridiplants) originate from a primary endosymbiosis event involving a cyanobacterium and a nonphotosynthetic eukaryote (Palmer 2003; Rodriguez-Ezpeleta et al. 2005; Gray and Archibald 2012). Although the number of retained cyanobacterial genes varies according to the lineage, the chloroplast genomes of Archaeplastida share many cyanobacterial-like operons and, except for those of red algae, generally contain two copies of a large inverted repeat (IR) encoding the rRNA operon (Green 2011).

How the chloroplast genome is changing through time is best understood for land plants, a branch of the Viridiplantae (green algae and land plants) that emerged about 450 Ma (Jansen and Ruhlman 2012; Wolf and Karol 2012). Studies of a large number of land plant chloroplast DNAs (cpDNAs) (mostly from seed plants) have uncovered the highly conservative nature of this organelle genome. The vast majority of seed plant cpDNAs is 107–218 kb in size and their 101–118 genes, which are interrupted by 21 introns, are dispersed among the IR and the large and small single-copy (LSC and SSC) regions with a nearly identical gene partitioning pattern (Jansen and Ruhlman 2012). The IR has been lost occasionally during land plant evolution; at least five independent losses have been documented in seed plants (Jansen and Ruhlman 2012). Although chloroplast gene order has been maintained over long evolutionary periods, extensive gene rearrangements have occurred in some angiosperm lineages (Jansen and Ruhlman 2012). The most common events underlying changes in land plant cpDNA architecture include alterations in gene order through sequence inversions (reversals) and the contraction/expansion of the IR.

The Viridiplantae also comprise the green algae, which are divided between the Streptophyta and Chlorophyta. The streptophyte algae or charophytes are the closest relatives of land plants and their chloroplast genome shares many similarities with their land plant counterparts (Turmel et al. 2007). Compared with their streptophyte homologs, chlorophyte chloroplast genomes exhibit a much greater diversity in genome and gene organization. To date, fewer than 30 chlorophyte chloroplast genomes have been described in the literature: They range from 64 to 525 kb in size, encode 88–128 standard genes (Lang and Nedelcu 2012), and a number of those containing a large IR display large deviations from the ancestral pattern of gene partitioning among the single-copy regions (in particular, chlorophycean and ulvophycean genomes), which is observed in streptophytes, some prasinophyceans, and the pedinophycean *Pedinomonas minor* (Maul et al. 2002; Pombert et al. 2005, 2006; de Cambiaire et al. 2006; Robbens et al. 2007; Brouard et al. 2008; Smith and Lee 2009; Turmel, Gagnon, et al. 2009; Smith et al. 2010; Lemieux et al. 2014b). Additional genomic changes experienced by chlorophyte chloroplast genomes include the loss of the IR, extensive gene rearrangements, expansion of gene and intergenic sequences, invasion by repeat elements and introns, acquisition of foreign genes by horizontal transfer, changes in nucleotide composition, and gene fragmentation (Maul et al. 2002; Bélanger et al. 2006; Pombert et al. 2005, 2006; de Cambiaire et al. 2006, 2007; Robbens et al. 2007; Turmel et al. 2008; Smith and Lee 2009; Turmel, Gagnon, et al. 2009; Turmel, Otis, et al. 2009; Brouard et al. 2008, 2011, 2010; Smith et al. 2010, 2011; Lemieux et al. 2014b).

Considering that the chloroplast genome experienced tremendous alterations within the Chlorophyta and that only a few taxa have been investigated in each of the major lineages of this division, it is still unclear what were the ancestral conditions of these lineages and whether distinct lineages differ in their evolutionary patterns. Of course, knowledge of the branching order among and within the main chlorophyte lineages is required to infer what genomic changes accompanied the emergence of new lineages. In this regard, the phylogeny of chlorophytes is in constant flux (Leliaert et al. 2012; Marin 2012; Fucikova et al. 2014; Lemieux et al. 2014a), and at this time, it is thought that the first branches of the Chlorophyta are occupied by prasinophycean lineages, with prasinophycean clade VII being sister to all core chlorophytes (Pedinophyceae + Chlorodendrophyceae + Chlorellales + Trebouxiophyceae + Ulvophyceae + Chlorophyceae).

**Fig. 1.—:**
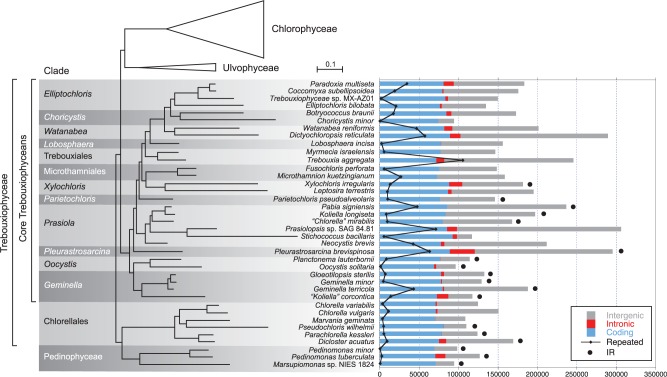
Phylogenetic relationships among the 38 core chlorophytes examined in this study and total lengths of coding, intronic, intergenic, and small repeated sequences (>30 bp) in their chloroplast genomes. The presence of a large IR encoding rRNA genes is also indicated. The best-scoring ML tree that Lemieux et al. (2014a) inferred from 79 cpDNA-encoded proteins under the GTR+Γ4 model is presented. Note that intron-encoded genes were not considered as coding sequences but rather as intron sequences and that the *O. solitaria*, *P. brevispinosa,* and *T. aggregata* genome sequences are not complete.

The Trebouxiophyceae is a species-rich class of the Chlorophyta that exhibits numerous lineages as evidenced by 18S rDNA analyses and displays remarkable variation in morphology and ecology (Lewis and McCourt 2004; Friedl and Rybalka 2012; Leliaert et al. 2012). It includes several species participating in symbiosis with fungi to form lichens, photosynthetic symbionts in ciliates, metazoan and plants, as well as species that have lost photosynthetic capacity. To identify the interrelationships between the major clades of trebouxiophyceans and gain information on the evolutionary history of the chlorophyte chloroplast genome, we recently sequenced the chloroplast genomes of 27 trebouxiophyceans and two pedinophyceans, thus bringing to 3 and 35 the total number of photosynthetic taxa analyzed for their chloroplast genome in the Pedinophyceae and Trebouxiophyceae (Lemieux et al. 2014a). Phylogenetic analyses of 79 cpDNA-encoded proteins and genes from 61 chlorophytes, including the 38 pedinophyceans and trebouxiophyceans, revealed that the Trebouxiophyceae is not monophyletic. Two major clades containing trebouxiophycean taxa were identified: A clade of 29 core trebouxiophyceans that is sister to the Ulvophyceae and Chlorophyceae, and a clade comprising the Chlorellales and Pedinophyceae that is sister to the core trebouxiophyceans + Ulvophyceae + Chlorophyceae (see fig. 1). Like most of the chlorellaleans, early-diverging core trebouxiophyceans are predominantly planktonic species, whereas core trebouxiophyceans occupying later-diverging lineages are mostly terrestrial or aeroterrestrial algae.

In this study, we report the structural features of the 29 newly sequenced chloroplast genomes that were used to reconstruct the abovementioned phylogenies and include in our comparative genome analysis the previously sampled members of the Pedinophyceae and Trebouxiophyceae (Wakasugi et al. 1997; de Cambiaire et al. 2007; Turmel, Otis, et al. 2009; Smith et al. 2011; Servin-Garciduenas and Martinez-Romero 2012). We sought to identify the main genomic changes that occurred in the various lineages investigated. The examined genomes display considerable variability at all levels except gene content. Our results highlight the high propensity of the rDNA-containing IR to vary in size, gene content and gene order, and the repeated losses it experienced during trebouxiophycean evolution. Overall, the structural genomic data provide independent support for many of the relationships we identified in our previous phylogenomic study.

## Materials and Methods

### Source and Annotations of Chloroplast Genomes

The pedinophycean and trebouxiophycean chloroplast genomes compared in this study are those that were used to construct the phylogenies recently reported by Lemieux et al. (2014a). GenBank accession numbers for all 38 genomes are provided in table 1. All genomes are available as complete genome sequences, except those of the core trebouxiophyceans *Oocystis solitaria* (1 contig)*, Pleurastrosarcina brevispinosa* (1 contig), and *Trebouxia aggregata* (41 contigs). The presence of abundant repeats in the *T. aggregata* genome prevented us from assembling the complete sequence.

**Table 1:** GenBank Accession Numbers and Main Features of the Chloroplast Genomes Examined in This Study

Taxon	Accession No.^a^	A+T	Size (bp)				Genes (no.)^b^	Introns^c^		Repeats
		(%)	Genome	IR	LSC	SSC		GI	GII	(%)^d^
*Marsupiomonas* sp. NIES 1824	KM462870*	59.7	94,262	9,926	68,185	6,225	106			0.3
*Pedinomonas tuberculata*	KM462867*	66.6	126,694	16,074	86,619	7,927	107	5	5	2.1
*Pedinomonas minor*	NC_016733	65.2	98,340	10,639	70,398	6,664	106			0
*Dicloster acuatus*	KM462885*	70.0	169,201	22,061	87,535	37,544	112	6		5.4
*Parachlorella kessleri*	NC_012978	70.0	123,994	10,913	88,297	13,871	112	1		4.0
*Pseudochloris wilhelmii*	KM462886*	63.3	109,775	12,798	66,211	17,968	113	1		4.2
*Marvania geminata*	KM462888*	61.8	108,470				113	1		3.0
*Chlorella vulgaris*	NC_001865	68.4	150,613				113	3		7.3
*Chlorella variabilis*	NC_015359	65.9	124,579				113	3		2.4
*Koliella corcontica*	KM462874*	72.0	117,543	15,891	77,346	8,415	105	8		11.6
*Geminella terricola*	KM462881*	67.3	187,843	18,786	139,317	10,954	109	1	1	22.7
*Geminella minor*	KM462883*	72.1	129,187	11,970	95,317	9,930	108	1	1	3.2
*Gloeotilopsis sterilis*	KM462877*	70.5	132,626	13,730	95,069	10,097	109	2	1	5.1
*Oocystis solitaria*	FJ968739^e^	71.0	>96,287	>378^f^	71,295		110	1	1	0.7
*Planctonema lauterbornii*	KM462880*	66.8	114,128	10,577	81,906	11,068	111	1		7.3
*Pleurastrosarcina brevispinosa*	KM462875*^e^	65.5	>295,314	45,468	>194,027 ^g^	10,351	111	16	3	21.3
*Neocystis brevis*	KM462873*	68.6	211,747				112	5		19.8
*Stichococcus bacillaris*	KM462864*	68.1	116,952	8,272	51,357	49,051	107	4	1	4.3
*Prasiolopsis* sp. SAG 84.81	KM462862*	64.9	306,152				108	7	1	23.1
“*Chlorella*” *mirabilis*	KM462865*	68.5	167,972	6,835	121,087	33,215	110			5.5
*Koliella longiseta*	KM462868*	68.6	197,094	10,619	141,677	34,179	111			4.0
*Pabia signiensis*	KM462866*	66.6	236,463	27,336	141,652	40,139	111			20.0
*Parietochloris pseudoalveolaris*	KM462869*	68.4	145,947	6,786	115,976	16,399	109			6.8
*Leptospira terrestris*	NC_009681	72.7	195,081				107	4		4.8
*Xylochloris irregularis*	KM462872*	60.3	181,542	28,473	76,371	48,225	110	15		7.1
*Microthamnion kuetzingianum*	KM462876*	65.3	158,609				107			16.7
*Fusochloris perforata*	KM462882*	64.9	148,459				107			3.5
*Trebouxia aggregata*	EU123962–EU124002^e^	65.2	>245,724				100	8		42.7
*Myrmecia israelensis*	KM462861*	69.6	146,596				112			3.6
*Lobosphaera incisa*	KM462871*	72.2	156,031				111	1		1.4
*Dictyochloropsis reticulata*	KM462860*	64.1	289,394				111	3	5	19.7
*Watanabea reniformis*	KM462863*	58.8	201,425				110	6	1	23.0
*Choricystis minor*	KM462878*	54.6	94,206				111			0
*Botryococcus braunii*	KM462884*	57.6	172,826				112	1	2	9.8
*Elliptochloris bilobata*	KM462887*	54.2	134,677				110	3		15.1
*Trebouxiophyceae* sp. MX-AZ01	NC_018569	42.3	149,707				114	4		0.9
*Coccomyxa subellipsoidea*	NC_015084	49.2	175,731				114	1		10.6
*Paradoxia multiseta*	KM462879*	49.4	183,394				114	14		18.6

^a^The asterisks denote the 29 genomes sequenced by Lemieux et al. (2014a) and described here for the first time.

^b^Intronic genes and freestanding ORFs not usually found in green plant chloroplast genomes are not included in these values. Duplicated genes were counted only once.

^c^Number of group I (GI) and group II (GII) introns is given.

^d^Nonoverlapping repeat elements were mapped on each genome with RepeatMasker using the repeats ≥30 bp identified with REPuter as input sequences.

^e^Because the *Oocystis solitaria, Pleurastrosarcina brevispinosa,* and *Trebouxia aggregata* chloroplast genomes are partially sequenced, the values reported for their sizes represent underestimates and those corresponding to other genomic features may be inaccurate.

^f^The exact sizes of the *O. solitaria* IR and SSC regions could not be determined because the IR/SSC junction has not been identified.

^g^The size of the *P. brevispinosa* LSC region was underestimated because this region contains a sequencing gap.

The methods that were used to generate and annotate the 29 chloroplast genomes are described in Lemieux et al. (2014a). The same methods were employed to reannotate previously described genomes to produce very high quality annotations. Coding sequences of nonstandard chloroplast genes were identified as follows: Free-standing open-reading frames (ORFs) of more than 100 codons were obtained using GETORF in EMBOSS 6.6.0 (Rice et al. 2000) and their translated products were subjected to BLASTP similarity searches against the nonredundant database at the National Center for Biotechnology Information (NCBI) (http://blast.ncbi.nlm.nih.gov/Blast.cgi, last accessed July 14, 2015). Only the ORFs with similarities to genes of known function were annotated. Intron types and boundaries were determined by modeling intron secondary structures (Michel et al. 1989; Michel and Westhof 1990) and by comparing intron-containing genes with intronless homologs. Circular and linear genome maps were drawn with OGDraw (Lohse et al. 2007).

### Analyses of Gene Organization

The sidedness index (*C*_s_) was determined as described by Cui et al. (2006) using the formula *C*_s_ = (*n* − *n*_SB_)/(*n* − 1), where *n* is the total number of standard genes in the genome and *n*_SB_ is the number of sided blocks, that is, the number of blocks including adjacent genes on the same strand.

Alignments of whole genomes from taxa belonging to selected clades were carried out using the ProgressiveMauve algorithm of Mauve 2.3.1 (Darling et al. 2010) after removal of one copy of the IR from the IR-containing genomes. The numbers of reversals separating all genome pairs in these clades were estimated with MGR 2.03 (Bourque and Pevzner 2002) using the permutation matrix file generated by Mauve, which records the order and orientation of locally collinear blocks.

The ancestral genomic reconstruction option of MLGO (Maximum Likelihood for Gene-Order Analysis) (Hu et al. 2014) was employed to predict the order of the 91 genes shared by all compared genomes at each internode of the amino acid-based phylogeny previously inferred by Lemieux et al. (2014a). The gene order matrix we analyzed took gene polarity into account and contained only one copy of the IR sequence and of other duplicated gene loci within the IR or SC region. The numbers of reversals separating the internal and terminal nodes of the genome rearrangement tree were computed using GRIMM 2.01 (Tesler 2002). For comparison of branch lengths, the genome rearrangement tree was scaled using Ktreedist (Soria-Carrasco et al. 2007) so that its global divergence was as similar as possible to that of the protein tree.

A maximum-likelihood (ML) tree was inferred using the phylogeny reconstruction option of MLGO and a matrix of gene order containing all standard genes, including copies of duplicated genes. Confidence of branch points was estimated by 1,000 bootstrap replications.

We used a custom-built program to identify the regions that display the same gene order in the compared chloroplast genomes. Gene order in each genome was converted to all possible pairs of signed genes and the presence/absence of the gene pairs shared by two or more genomes was coded as binary characters in Mesquite 3.01 (Maddison WP and Maddison DR 2015). Gains/losses of gene pairs that occurred during the evolution of pedinophycean and trebouxiophycean taxa were identified by tracing these characters with MacClade 4.08 (Maddison DR and Maddison WP 2000) under the Dollo principle of parsimony on the tree topology inferred by Lemieux et al. (2014a).

### Analyses of Repetitive Sequences

To estimate the proportion of repeated sequences in individual chloroplast genomes, repeats ≥30 bp were retrieved using the REPFIND program of REPuter 2.74 (Kurtz et al. 2001) with the options -f (forward) -p (palindromic) -l (minimum length = 30 bp) -allmax and then masked on the genome sequence using REPEATMASKER (http://www.repeatmasker.org/, last accessed July 14, 2015) running under the cross_match search engine (http://www.phrap.org/, last accessed July 14, 2015). The repeats identified by BLASTN 2.2.30+ searches of each chloroplast genome against itself (word size = 30) were defined into distinct elements using RECON 1.08 (Bao and Eddy 2002) and these elements were then classified in different groups of size intervals. The G+C contents of the repeated and unique sequences within each chloroplast genome were calculated from the outputs of REPEATMASKER that were generated with the -xsmall option (under this option the repeat regions are returned in lower case and nonrepetitive regions in capitals in the masked file).

### G+C Content of Protein-Coding Genes

The G+C content of protein-coding genes was determined at each codon position using DAMBE (Xia 2013) and the concatenated nucleotide data set (79 genes, 15,468 codons) of Lemieux et al. (2014a).

## Results

### A+T Content, Genome Size, and Presence/Absence of IR

The maps of the 29 newly sequenced chloroplast genomes are shown in supplementary figure S1, Supplementary Material online, along with those previously reported for their homologs in the Pedinophyceae and Trebouxiophyceae. The main structural features of these genomes are summarized in table 1. All 38 compared chloroplast genomes, except three from the Trebouxiophyceae (*T. aggregata*, *P. brevispinosa,* and *O. solitaria*), have been completely sequenced. As expected, most of these genomes are rich in A+T (table 1). Only six, all from core trebouxiophyceans belonging to the *Elliptochloris* + *Choricystis* clade, have an A+T content of less than 58.0% and among these, the most G+C-biased genome, with 42.3% A+T, is that of Trebouxiophyceae sp. MX-AZ01.

Total chloroplast genome size in our study group ranges from 94,206 (in *Choricystis minor*) to 306,152 pb (in *Prasiolopsis* sp. SAG 84.81) and varies markedly within some individual trebouxiophycean lineages (table 1 and fig. 1). For example, in the *Prasiola* clade, the genome of the minute alga *Stichococcus bacillaris* is 2.6-fold smaller than that of its closest relative, *Prasiolopsis* sp. SAG 84.81. Most of the genomes smaller than 150 kb are found in the Pedinophyceae, Chlorellales, and the *Geminella* + *Oocystis* clade, whereas those larger than 190 kb are restricted to core trebouxiophycean lineages that diverged after the *Geminella* + *Oocystis* clade.

**Fig. 2.—:**
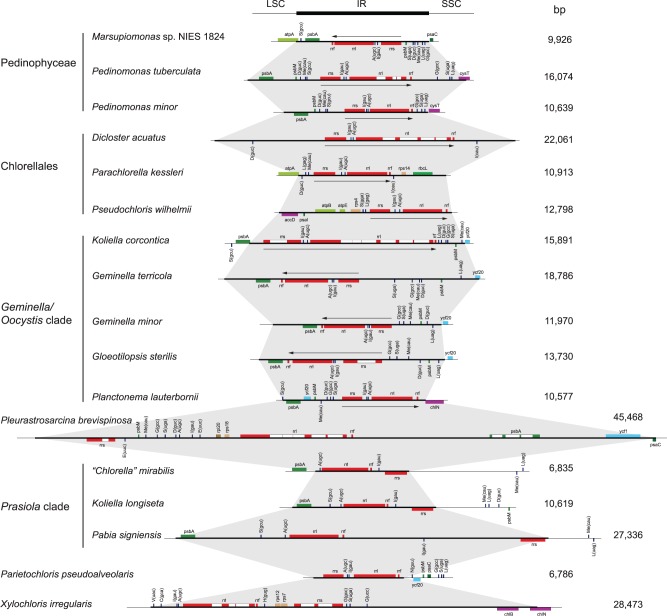
Gene organization of the large IRs in the chloroplast genomes examined in this study. Coding sequences of the rRNA genes are represented in red and, for all the IRs featuring an ancestral rDNA operon, the direction of transcription of this operon is shown by an arrow. The *O. solitaria* IR is not represented because its extent remains unknown. All gene maps are drawn to scale.

Only 18 of the 38 compared chloroplast genomes possess a large IR, part of which encodes the rRNA genes (table 1 and fig. 1). Taxa lacking such an IR are found in the Chlorellales (three of the six algae sampled from this clade) and in core trebouxiophycean lineages that diverged after the *Geminella + Oocystis* clade (three of the six algae examined in the *Prasiola* clade, three of the four representatives of the Microthamniales + *Xylochloris* clade, and all members from the superclade containing the Trebouxiales and the *Lobosphaera, Watanabea*, *Choricystis,* and *Elliptochoris* clades). The IR shows important fluctuation in size both among and within lineages (fig. 2). The smallest (6.8 kb) and largest (45.5 kb) IRs are found in core trebouxiophyceans representing independent lineages: *Parietochloris pseudoalveolaris* and *P. brevispinosa,* respectively. Among the lineages represented by multiple taxa, the *Prasiola* clade displays the most important IR size variation (4-fold). Note here that a member of this clade, *S. bacillaris,* exhibits two copies of a 8,272-bp sequence that are inverted relative to one another and separated by similar sized single-copy regions; however, this IR lacks the rRNA genes (table 1 and supplementary fig. S1, Supplementary Material online).

Like the IR size, the proportion of noncoding sequences (i.e., introns and intergenic regions) in the examined cpDNAs is highly variable both among and within lineages (fig. 1). The intergenic regions, which represent up to 68% of the genome (in *Prasiolopsis*), are the noncoding sequences contributing the most to the observed genome size variation. The largest genomes (>200 kb) generally contain not only the highest amount of intergenic regions but also the greatest abundance of repeats of more than 30 bp (table 1, fig. 1, and supplementary fig. S2, Supplementary Material online). With 42.7% of small repeats, the genome of the lichen symbiotic *T. aggregata*, whose 41 contigs total 245.7 kb, is the most repeat-rich genome identified in our study. In general, the genomes with the greatest proportions of repeated sequences contain the largest numbers of distinct repeat elements (supplementary fig. S2, Supplementary Material online). In any given genome, small repeats are generally heterogeneous in size, composed of direct as well as palindromic sequences, and richer in G+C content than unique sequences (supplementary fig. S2, Supplementary Material online). The proportions of distinct repeats assigned to six categories of size intervals (30–39, 40–59, 60–89, 90–149, 150–249, and >250 bp) reveal that the distribution of repeat sizes is variable among and within lineages (supplementary fig. S2, Supplementary Material online).

### Standard Genes

**Fig. 3.—:**
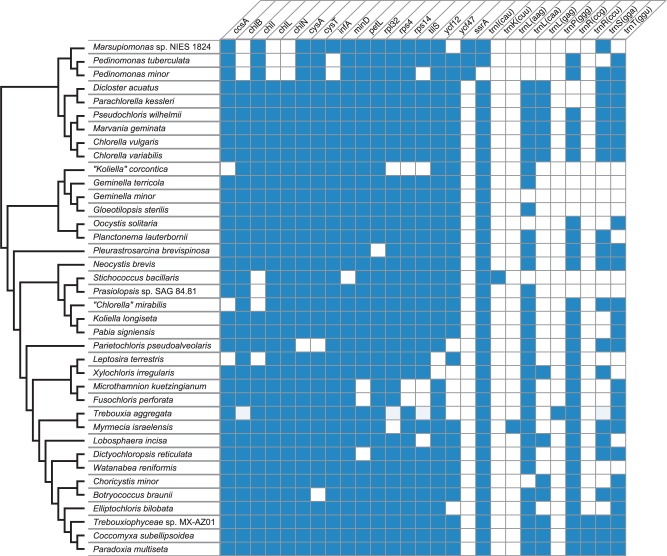
Gene repertoires of the chloroplast genomes examined in this study. Only the genes that are missing in one or more genomes are indicated. The presence of a standard gene is denoted by a blue box. A total of 91 genes are shared by all compared genomes that have been completely sequenced: *accD, atpA, B, E, F, H, I, cemA, clpP, ftsH, petA, B, D, G, psaA, B, C, I, J, M, psbA, B, C, D, E, F, H, I, J, K, L, M, N, T, Z, rbcL, rpl2, 5, 12, 14, 16, 19, 20, 23, 36, rpoA, B, C1, C2, rps2, 3, 7, 8, 9, 11, 12, 18, 19, rrf, rrl, rrs, tufA, ycf1, 3, 4, 20, trnA*(ugc), *C*(gca), *D*(guc), *E*(uuc), *F*(gaa), *G*(gcc), *G*(ucc), *H*(gug), *I*(gau), *K*(uuu), *L*(uaa), *L*(uag), *Me*(cau), *Mf*(cau), *N*(guu), *P*(ugg), *Q*(uug), *R*(ucu), *R*(acg), *S*(gcu), *S*(uga), *T*(ugu), *V*(uac), *W*(cca), and *Y*(gua). Eight of these genes (*petG*, *psbI*, *trnI*(gau), *L*(uaa), *P*(ugg), *R*(ucu), *S*(gcu), *T*(ugu)) have not been identified in the partial chloroplast genome sequence of *T. aggregata*. Note that *ycf12* (*psb30*) codes for a subunit of the photosystem II complex (Kashino et al. 2007).

The 35 completely sequenced cpDNAs contain 105–114 unique standard genes, that is, genes usually present in chloroplast genomes (table 1). Included in this category are all identified tRNA genes, even though four of these genes ((*trnK*(cuu), *trnL*(aag), *trnP*(ggg), and *trnR*(ccu)) occur rarely in chlorophyte cpDNAs (fig. 3). All genomes share a set of 91 genes coding for three rRNAs (*rrs*, *rrl,* and *rrf*), 25 tRNAs, and 63 proteins (see legend of fig. 3). As expected, virtually all standard genes in IR-less genomes are present in one copy; the only exceptions are the *Marvania geminata trnG*(gcc) and *Prasiolopsis rrf* genes, which occur in two identical and nonidentical copies (106/121 identity), respectively. These duplicated copies of these two genes may represent remnants of an ancestral IR. Note that *trnG*(gcc) is located near the IR/LSC boundary in the closely related alga *Pseudochloris wilhemii*.

Three protein-coding genes, the *rpoB* and *rpoC2* genes encoding subunits of the RNA polymerase and the *tilS* gene encoding the tRNA(Ile)-lysidine synthetase, are fragmented and are not associated with sequences typical of group I or group II introns in several core trebouxiophyceans (supplementary fig. S3, Supplementary Material online). The pieces of these fragmented genes are contiguous on all genome sequences, except for the fragments of the *Xylochloris irregularis* and *Watanabea reniformis tilS*. Fragmented structures have been previously reported for *rpoB* (Bélanger et al. 2006; de Cambiaire et al. 2006, 2007; Turmel et al. 2008; Brouard et al. 2008, 2010, 2011) and *rpoC2* (Turmel et al. 2008) of other core chlorophytes and in the case of *rpoB*, it was observed that the genes of *Leptosira terrestris* and of three chlorophycean green algae are fragmented at the same site, near the junction of a conserved segment of 80 codons and a highly variable region (de Cambiaire et al. 2007). Alignments of the proteins encoded by the *rpoB*, *rpoC2,* and *tilS* genes examined in the present investigation revealed that all these genes, except the *S. bacillaris rpoB,* share common fragmentation sites. Note that the *P. brevispinosa rpoC2* gene exhibits three sites of fragmentation: The first site corresponds to that found in the *Chlamydomononas moewusii* gene (Turmel et al. 2008), whereas the second site corresponds to those in the *S. bacillaris* and *W. reniformis* genes.

The standard genes accounting for the variable coding capacity of the analyzed chloroplast genomes consist of 16 protein-coding genes, 10 tRNA genes and the gene for tmRNA (*ssrA*), a small regulatory RNA that has both mRNA and tRNA activities and interacts with stalled ribosomes to resume translation on the SsrA mRNA moiety (supplementary fig. S4, Supplementary Material online). Although *rpl32* is missing only in the incompletely sequenced genome of *P. brevispinosa*, this gene loss appears genuine because our search for *rpl32* in the sequence assembly of total cellular DNA proved unsuccessful. The *ssrA* gene is present in the three taxa sampled from the Pedinophyceae but appears to be absent from the chloroplast genomes of the investigated trebouxiophyceans. This gene was previously identified in the chloroplasts of the streptophyte *Mesostigma viride* and the prasinophycean *Nephroselmis olivacea* (Gueneau de Novoa and Williams 2004). In the course of this study, we also localized it in the recently sequenced cpDNAs of two additional prasinophyceans (*Nephroselmis astigmatica* and *Picocystis salinarum*) (Lemieux et al. 2014b), using a Smith–Waterman search for similarity and the 5′ and 3′ conserved regions of standard one-piece tmRNAs as query sequences. The 5′ and 3′ terminal sequences composing the tRNA-like domains as well as the internal mRNA-like coding region are conserved in chlorophyte and streptophyte *ssrA* genes (supplementary fig. S4, Supplementary Material online)*.* All six known chlorophyte *ssrA* genes reside in the immediate vicinity of *rbcL* and are encoded on the same DNA strand (supplementary fig. S1, Supplementary Material online).

Although the *trnR*(ccu) gene is restricted to three taxa of the *Elliptochloris* clade, *trnK*(cuu), *trnL*(aag) and *trnP*(ggg) are found exclusively in *S. bacillaris*, *Myrmecia israelensis* and *T. aggregata*, respectively (fig. 3)*.* Our BLASTN similarity searches against the nonredundant database of NCBI suggest that each of the four tRNA genes arose from duplication and subsequent sequence divergence of an existing chloroplast gene: *trnR*(ccu) originated from *trnR*(ucu), *trnK*(cuu) from *trnK*(uuu), *trnL*(aag) from *trnL*(uag), and *trnP*(ggg) from *trnG*(gcc). Prior to our study, the prasinophycean *Pycnococcus provasolii* was the only known chlorophyte carrying *trnP*(ggg) in its chloroplast (Turmel, Gagnon, et al. 2009) and aside from *Coccomyxa subellipsoideae*, *trnR*(ccu) had been localized only in the chloroplasts of the chlorophycean *Oedogonium cardiacum* (Brouard et al. 2008) and the ulvophyceans *Pseudendoclonium akinetum* and *Oltmannsiellopsis viridis* (Pombert et al. 2005, 2006).

Additional tRNA genes (*trnD*(guc), *trnE*(uuc), *trnG*(gcc), and *trnG*(uuu)) were likely duplicated in other lineages of the Trebouxiophyceae, yielding identical copies. The two *trnG*(gcc) sequences in the IR-less genome of *M. geminata* are either the products of a duplication event or as mentioned earlier, the remnants of an ancestral IR. One of the two *trnN*(guu) loci in the partially assembled *O. solitaria* genome lies just 3′ of the rDNA operon and may therefore be part of the IR (supplementary fig. S1, Supplementary Material online). The *Dicloster acuatus trnD*(guc) sequences are present in the IR and LSC regions, whereas both loci of *P. brevispinosa trnE*(uuc) map within the IR. Duplicates of the latter gene have also been reported in the chloroplasts of other chlorophytes (de Cambiaire et al. 2006; Brouard et al. 2010).

### Proportion of G+C in Standard Protein-Coding Genes

Given the important range of variation in G+C content observed for the compared chloroplast genomes, we examined the G+C composition of protein-coding genes at each codon position among these genomes using the concatenated nucleotide data set (79 genes from 63 taxa, 15,468 codons) analyzed by Lemieux et al. (2014a). We found that the G+C content at third codon positions ranges from 10% to 25% in the majority of examined chlorophyte cpDNAs (supplementary fig. S5, Supplementary Material online). In contrast, higher G+C values ranging from 29% (*Elliptochloris bilobata*) to 64% (Trebouxiophyceae sp. MX-AZ01) are observed at third codon positions for the G+C-biased genomes characterizing all members of the *Elliptochloris* + *Choricystis* clade. The compositional bias is much less pronounced at the functionally constrained first and second codon positions. Interestingly, two other chlorophytes with a relatively high G+C content in their chloroplast genomes (*X. irregularis*, 39.7% G+C and *Marsupiomonas* sp. NIES 1824, 40.3% G+C) have a G+C content of more than 30% at the third codon positions of their protein-coding genes.

### Unusual Genes

We discovered potential coding sequences that are not usually found in green plant chloroplast genomes by carrying out BLASTP similarity searches against the nonredundant NCBI database using as query sequences free-standing ORFs of more than 100 codons. ORFs showing similarities (*E*-value threshold of 1e-06) with proteins of known functions and/or recognized protein domains were identified in 13 of the examined genomes and grouped into ten categories according to their putative function/domain (table 2). All of these ORFs encode putative products acting on DNA or RNA. In all five instances where a given ORF is found in different species, we find that the latter belong to different lineages. Three individual genomes, those of *Paradoxia multiseta*, *Prasiolopsis* sp., and *Dicloster acuatus*, exhibit two or more ORFs with the same function and/or recognized protein domain; in these cases, nonidentical copies are present in each genome.

**Table 2:** Nonstandard Genes Identified as Freestanding ORFs in the Chloroplast Genomes Examined in This Study

Taxon	ORF^a^	Genomic Coordinates	Conserved Domain
*Neocystis brevis*	148	110000–109554	DNA breaking-rejoining enzymes, C-terminal catalytic domain (cd00397)
*Paradoxia multiseta*	119	105729–106088	DNA breaking-rejoining enzymes, C-terminal catalytic domain (cd00397)
*Paradoxia multiseta*	298	35212–36108	DNA breaking-rejoining enzymes, C-terminal catalytic domain (cd00397)
*Prasiolopsis* sp. SAG 84.81	154	277159–277623	Integrase core domain (pfam00665)
*Prasiolopsis* sp. SAG 84.81	298	296164–297060	Integrase core domain (pfam00665)
*Prasiolopsis* sp. SAG 84.81	200	274101–274703	Putative integrase/recombinase
*Dictyochloropsis reticulata*	102	51790–51482	Serine recombinase family, resolvase and invertase subfamily, catalytic domain (cd03768)
*Botryococcus braunii*	117	24161–23808	Phage-associated DNA primase (COG3378)
*Prasiolopsis* sp. SAG 84.81	653	183296–185257	Phage/plasmid primase, P4 family, C-terminal domain (TIGR01613)
*Watanabea reniformis*	403	111049–112260	Primase C terminal 1 (smart00942)
*Dicloster acuatus*	153	116412–116873	DNA polymerase type-B family catalytic domain (cd00145)
*Dicloster acuatus*	328	93575–94561	DNA polymerase type-B alpha subfamily catalytic domain (cd05532)
*Marvania geminata*	242	8571–7843	Deoxyribonucleoside kinase (cd01673)
*Microthamnion kuetzingianum*	139	12286–12705	Type II restriction endonuclease *Nla*III; HNH endonuclease
*Pedinomonas tuberculata*	214	109148–109792	*Hae*III restriction endonuclease (pfam09556)
*Chlorella variabilis*	123^b^	99567–99938	N-6 DNA methylase (pfam02384)
*Chlorella variabilis*	338^b^	100401–101417	N-6 DNA methylase (pfam02384)
*Chlorella variabilis*	152^b^	101377–101835	N-6 DNA methylase (pfam02384)
*Chlorella variabilis*	175^c^	19229–19756	LAGLIDADG DNA endonuclease family (pfam00961)
*Neocystis brevis*	331^c^	21453–22448	LAGLIDADG DNA endonuclease family (pfam03161)
*Trebouxiophyceae* sp. MX-AZ01	119^c^	7868–8227	LAGLIDADG DNA endonuclease family (pfam03161)
*Dictyochloropsis reticulata*	671^c^	127765–129780	Reverse transcriptase with group II intron origin (cd01651)
			Group II intron, maturase-specific domain (pfam08388)
*Pleurastrosarcina brevispinosa*	214^c^	282883–283527	Reverse transcriptase with group II intron origin (cd01651)

^a^Reported here are the freestanding ORFs larger than 100 codons that revealed similarity (*E*-value threshold of 1e-06) with proteins of known function and/or recognized protein domains in our BLASTP searches. Each ORF is identified by the number of amino acid residues in the encoded protein.

^b^The *orf123*, *orf338,* and *orf152* of *Chlorella variabilis* may be part of a larger ORF considering that they are contiguous on the genome sequence and all show similarity to N-6 DNA methylases.

^c^These ORFs are not encoded within recognizable group I and group II intron sequences and thus appear to be free-standing.

Interestingly, three members of the *Prasiola* clade (*Neocystis brevis*, *Pabia signiensis,* and “*Chlorella” mirabilis*) share with the deep-sea γ-proteobacterium *Marinobacter manganoxydans* an ORF encoding a hypothetical protein (supplementary fig. S6, Supplementary Material online). This hypothetical gene is located within the IR in *Pa. signiensis,* at two distinct sites in “*Chlorella” mirabilis* and at four sites in *N. brevis* (supplementary fig. S1, Supplementary Material online).

### Gene Partitioning Patterns between the IR and Single-Copy Regions

**Fig. 4.—:**
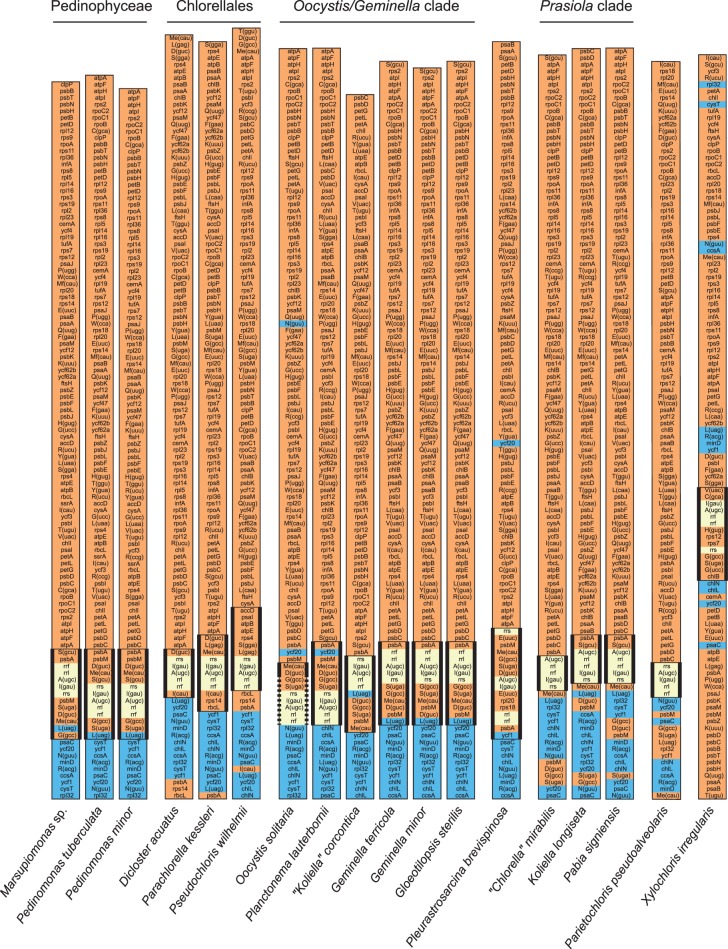
Gene partitioning patterns of the IR-containing chloroplast genomes examined in this study. The IRs span the sequence delimited by thick vertical lines; only the IR/LSC junction was identified in the *O. solitaria* genome, with the sequence corresponding to the dotted lines being most likely part of the IR. Note that the gene sequences spanning the IR/SSC or IR/LSC junction are represented in the SSC or LSC region, respectively. The five genes composing the rDNA operon are highlighted in yellow. The color assigned to each of the remaining genes is dependent upon the position of the corresponding gene relative to the rDNA operon in previously reported IR-containing prasinophycean and streptophyte cpDNAs displaying an ancestral gene partitioning pattern. The genes highlighted in blue are found within or near the SSC region in ancestral genomes (downstream of the rDNA operon), whereas those highlighted in orange are found within or near the LSC region (upstream of the rDNA operon).

All 18 chloroplast genomes containing a large rDNA-encoding IR, with a single exception (the *X. irregularis* genome), display a pattern of gene partitioning that closely resembles the pattern observed for several prasinophycean and streptophyte algae (fig. 4). However, a few genes typically located 5′ of the rDNA operon (i.e., near the LSC region) in prasinophycean and streptophyte genomes are found 3′ of the rRNA operon or near/within the SSC region in the pedinophycean and most trebouxiophycean IR-containing genomes. In addition, genes ancestrally located 3′ of the rDNA operon (i.e., near/within the SSC region) have been shifted to the LSC side in four of the analyzed genomes. The genomes of the two trebouxiophyceans from the *Oocystis* clade show the most similarity to the ancestral partitioning pattern.

All genes found in the pedinophycean IRs, with two exception (*psbA* and *trnS*(gcu)), are also IR-encoded in all four members of the *Geminella* clade. Included in this gene set are the genes present in the SSC regions of the prasiolalean genomes, which are not typically found in the SSC region in genomes exhibiting the ancestral partitioning pattern. Based on the gene content differences observed for the IR in trebouxiophycean lineages, it is clear that the IR/SSC and IR/LSC boundaries each underwent frequent shifts in both directions (i.e., either toward the neighboring single-copy region or toward the IR) during evolution.

Numerous differences in gene order are also observed between the IRs of the analyzed genomes. For example, in the IRs of *Marsupiomonas* and three of the four representatives of the *Geminella* clade, the position of the rDNA operon relative to the SSC region differs from that generally found in prasinophycean and streptophyte cpDNAs, implying that inversions of sequences within the IR occurred frequently. Genes within the rDNA operon were not spare from such rearrangements in the *Prasiola* clade and the *Parietochloris pseudoalveolaris* and *X. irregularis* lineages.

### Gene Organization

Considering that adjacent genes in the chloroplast genomes of several core chlorophytes have been reported to exhibit a strong propensity to be located on the same strand (de Cambiaire et al. 2007), we examined the degree to which neighboring genes are clustered on the same strand in pedinophycean and trebouxiophycean chloroplast genomes. This feature is best evaluated using the *C*_s_ index of Cui et al. (2006); when *C*_s_ reaches the maximum value of 1, all genes are located on one strand. The calculated *C*_s_ values vary from 0.66 (*Chlorella vulgaris*) to 0.88 (*L. terrestris*) and their distribution is generally not correlated with phylogenetic relationships (supplementary fig. S7, Supplementary Material online). The chloroplast genomes with the most “sided” structures are found in the *Parietochloris* and *Xylochloris* + Microthamniales lineages, whereas those with the lowest *C*_s_ values are found in the *Chlorella* and *Geminella* + *Oocystis* lineages.

To gain insight into the extent of sequence rearrangements in the compared chloroplast genomes, we aligned genomes from the Pedinophyceae and various clades of the Trebouxiophyceae using the ProgressiveMauve algorithm of Mauve 2.3.1 (Darling et al. 2010) (supplementary fig. S8, Supplementary Material online). Moreover, for each of these clades, the permutation matrix file generated by Mauve, which records the order and orientation of each locally collinear block, was analyzed with MGR (Bourque and Pevzner 2002) in order to determine the numbers of reversals separating all genome pairs (supplementary fig. S8, Supplementary Material online). The results revealed that the genomes of the Pedinophyceae and the *Geminella* + *Oocystis* clade (with 3–17 and 1–19 reversals, respectively) underwent fewer changes in sequence order compared with the four other clades examined (maximal number of reversals, 39, was observed for the *Prasiola* clade).

**Fig. 5.—:**
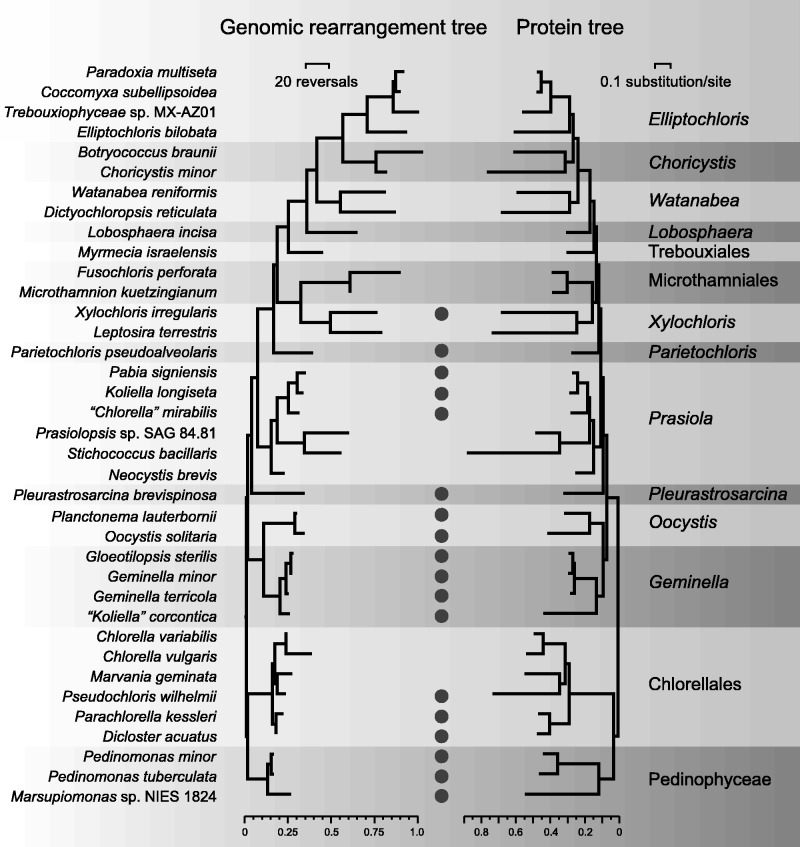
Extent of gene rearrangements in the chloroplast genomes examined in this study. A signed gene-order matrix of the 91 genes shared by all compared genomes was used to predict the number of sequence reversals on each branch of the best-scoring ML tree inferred from 79 cpDNA-encoded proteins (Lemieux et al. 2014a). For comparison of branch lengths, both the genome rearrangement and protein trees are represented; the genome rearrangement tree was scaled using Ktreedist (Soria-Carrasco et al. 2007) so that its global divergence is as similar as possible to that of the protein tree. The gray circles denote the genomes containing a large IR. The partially sequenced *T. aggregata* chloroplast genome was not included in this analysis because it is available as multiple contigs and lacks several genes.

Because the progressiveMauve algorithm has difficulty identifying locally collinear blocks in genomic regions with important sequence divergence and can thus underestimate the number of genome rearrangements (Darling et al. 2010; Kittichotirat et al. 2010), the extent of sequence rearrangements in the examined chloroplast genomes was also evaluated using a matrix of signed gene order for the 91 standard genes shared by all genomes (without duplicated copies). This matrix was applied to MLGO (Hu et al. 2014) and GRIMM (Tesler 2002) to generate a genome rearrangement tree showing the extent of gene reversals on each branch of the topology inferred from cpDNA-encoded protein sequences (fig. 5). The maximal number of observed rearrangements reached 150 reversals in this analysis (between the *Botryococcus braunii* and *Dictyochloropsis reticulata* genomes). In agreement with the abovementioned results, it was found that the genomes from the most basal lineages (Pedinophyceae, Chlorellales, and *Geminella* + *Oocystis* clade) are more conserved in gene order than those from the *Prasiola*, *Xylochloris* + Microthamniales, and Trebouxiales +*Lobosphaera* + *Watanabea + Choricystis* + *Elliptochloris* clades. In contrast to the protein tree, the genome rearrangement tree displays longer internodes and branches in the portion containing the core trebouxiophycean lineages that diverged after the *Oocystis*/*Geminella* clade (supplementary fig. S9, Supplementary Material online).

We also tested the performance of gene order data in resolving phylogenetic relationships among pedinophycean and trebouxiophycean taxa. For this purpose, we used the phylogenetic reconstruction option of MLGO; this likelihood-based phylogenetic inference tool encodes gene order data as binary characters and provides bootstrap support for genomic changes including not only gene rearrangements but also gene insertions, deletions, and duplications. Using a gene order matrix containing all standard genes, including copies of duplicated genes, we recovered a tree that is largely congruent with the topology inferred from cpDNA-encoded protein sequences (supplementary fig. S10, Supplementary Material online). This gene order tree exhibits several of the monophyletic groups identified in the protein tree (the Pedinophyceae, Chlorellales, Microthamniales, and the *Geminella, Oocystis, Prasiola* and *Elliptochloris* + *Choricystis* lineages) but the interrelationships between these lineages are mostly unresolved. Consistent with the rearrangement tree inferred with the ancestral genomic reconstruction option of MLGO (fig. 5), longer branches were observed for the late-diverging lineages of core trebouxiophyceans.

**Fig. 6.—:**
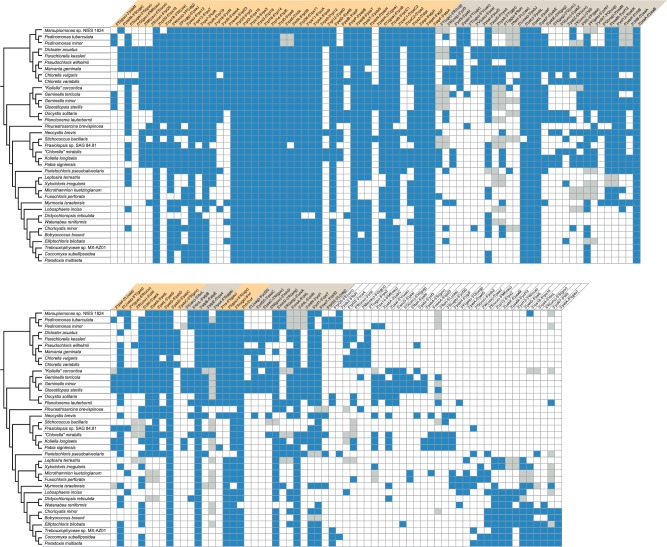
Distribution of shared gene pairs in the chloroplast genomes examined in this study. Among all possible gene pairs in the signed gene-order matrix of the 91 genes common to all compared taxa, we selected those that are shared between at least five taxa. The presence of a gene pair is denoted by a blue box. A gray box refers to a gene pair in which at least one gene is missing due to gene loss. Gene pairs were organized in blocks of contiguous gene pairs (shown as alternating colors) to facilitate the identification of conserved gene clusters. The partially sequenced *T. aggregata* chloroplast genome was not included in this analysis because it is available as multiple contigs and lacks several genes.

To uncover the gene clusters that are conserved in pedinophycean and trebouxiophycean chloroplast genomes, we analyzed all possible gene pairs in these individual genomes and identified those that are shared between at least five taxa (fig. 6). Numerous gene pairs and gene clusters are present in nearly all examined genomes and as expected, most of these gene linkages represent polycistronic units inherited from the cyanobacterial ancestor of chloroplasts. Consistent with the observation that core trebouxiophyceans from the most derived lineages have experienced more frequent genome rearrangements, a number of gene pairs are uniformly present in early-diverging lineages but missing in later-diverging core trebouxiophycean lineages, whereas derived forms of gene pairs are found exclusively in the latter lineages. Genome rearrangements led to the disruption of several ancestral operons, including the rDNA, *atpA*, *petA*, *psbB*, *psbE*, *rpoB,* and ribosomal protein operons. The rDNA operon is broken at one or more sites in most, if not all, core trebouxiophycean taxa from lineages that diverged after the *Geminella* + *Oocystis* clade (see supplementary fig. S11, Supplementary Material online). For example, in *P. brevispinosa*, there are breakpoints between *rrs* and *trnI*(gau) and between *trnA*(ugc) and *rrl,* and in the Prasiolales, all taxa share common breakpoints between *trnI*(gau) and *trnA*(ugc) and between *rrs* and *trnI*(gau). Only *N. brevis* (Prasiolales) displays linked *rrs* and *trnI*(gau) genes; however, given that the spacer separating these genes is very long (3.2 kb), it is possible that they are not cotranscribed and that their linkage is the result of secondary rearrangements.

### Intron Content

**Fig. 7.—:**
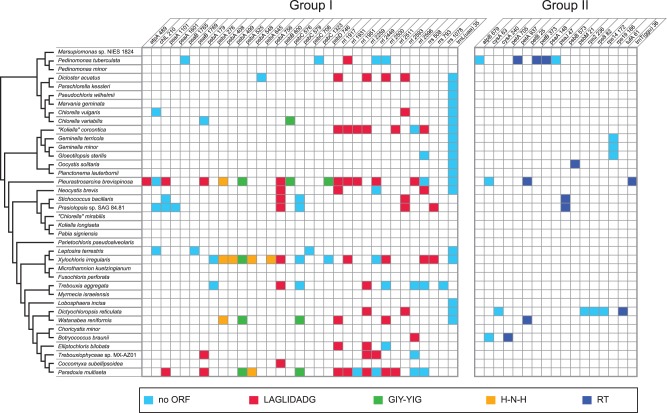
Distribution of group I and group II introns among the chloroplast genomes examined in this study. A light blue box represents an intron lacking an ORF, whereas a colored box represents an intron containing an ORF (see the color code at the bottom of the figure for the type of intron-encoded protein). Intron insertion sites in protein-coding and tRNA genes are given relative to the corresponding genes in *Mesostigma* cpDNA; insertion sites in *rrs* and *rrl* are given relative to the *Escherichia coli* 16S and 23S rRNAs, respectively. For each insertion site, the position corresponding to the nucleotide immediately preceding the intron is reported. Abbreviations: LAGLIDADG, LAGLIDADG homing endonuclease; GIY-YIG, GIY-YIG homing endonuclease; H-N-H, H-N-H homing endonuclease; RT, reverse transcriptase and/or intron maturase and/or H-N-H endonuclease.

Twenty-eight of the 38 chloroplast genomes examined carry group I and/or group II introns, all of which are *cis*-spliced (table 1). In any given genome, intron number varies between 1 (in ten taxa) and 19 (in *P. brevispinosa*). As expected for mobile genetic elements, the intron distribution is very irregular (fig. 7). Although the three smallest complete genomes lack introns, intron abundance is not necessarily correlated with genome size (fig. 1). For instance, the 117-kb *Koliella corcontica* and 306-kb *S. bacillaris* genomes (the latter being the largest genome investigated) each carry eight introns, whereas several genomes of intermediate size have only one or none.

Group I introns were found to be more frequent than group II introns (126 vs. 22) (fig. 7). We identified a total of 33 distinct group I intron insertion sites that are distributed among 11 genes, with 20 sites located in just three genes (*psbA*, *rrl,* and *rrs*). For group II introns, we observed 17 insertion sites among 14 genes. Unlike the group II intron positions, most of the individual group I intron sites occur in two or more unrelated taxa. The only two group II intron positions shared by more than one taxon (the *psbB* and *rps19* sites) are both lineage-specific.

At least half of the group I (70/126) and group II (11/22) introns we identified contain ORFs (fig. 7). The group I introns encode three different types of homing endonucleases (LAGLIDADG, GIY-YIG, and H-N-H) that drive the mobility and persistence of their own coding sequences (Stoddard 2014). The LAGLIDADG endonuclease genes (total of 54) represent the most prevalent type of intron ORFs: They occur at 14 of the 21 sites displaying ORF-containing group I introns, including all sites found within the rRNA genes. Interestingly, the same type of endonuclease gene is observed in all introns occupying a given site. The group II introns encode putative reverse transcriptases and/or intron maturases and/or H–N–H endonucleases (the domains identified in individual intron ORFs are annotated in the GenBank accessions of the chloroplast genome sequences). These proteins stabilize the catalytically active RNA structure for forward and reverse splicing, and convert the integrated intron RNA back into DNA (Lambowitz and Zimmerly 2011).

## Discussion

With the study reported here, the Trebouxiophyceae currently represents the class of green algae that has been the most broadly sampled for its chloroplast genome. Prior to our comparative analyses, relatively little was known about the extent of chloroplast genomic changes throughout this algal group. We investigated 35 trebouxiophycean taxa and three pedinophyceans and found that the chloroplast genome experienced important alterations at multiple levels during the evolution of the Trebouxiophyceae and Pedinophyceae.

Most of the genomes analyzed comprise 107–113 different standard genes (table 1 and fig. 1); thus, the size of the gene complement closely resembles those reported for a number of ulvophycean and prasinophycean chloroplast genomes (Pombert et al. 2005, 2006; Turmel, Gagnon, et al. 2009; Lemieux et al. 2014b) but is larger compared with the chlorophycean genomes investigated so far (Brouard et al. 2010, 2011). Despite the similarity in gene content, genome size displays a 3.3-fold variation (i.e., from 94 to 306 kb), and the differences observed are mainly attributable to a combination of three types of changes: Fluctuations in length of intergenic regions (which in turn are highly correlated with the abundance of small repeats), changes in intron content, and contractions/expansions of the large IR (fig. 1). Extensive variation in the proportion of noncoding sequences can be seen even within individual lineages, as exemplified by the 117- and 306-kb IR-less genomes of the closely related prasiolalean species *Prasiolopsis* sp. and *Stichococcus bacillaris*. Pico- and nanoplanktonic taxa (*Cho. minor, Marsupiomonas* sp. NIES 1824, *M. geminata*, *Pe. minor,* and *Ps. wilhelmii*) tend to carry the smallest genomes (see supplementary fig. S12, Supplementary Material online), a correlation that had been previously noted in prasinophyceans (Lemieux et al. 2014b).

### Repeated Losses of the IR during Trebouxiophycean Evolution

**Fig. 8.—:**
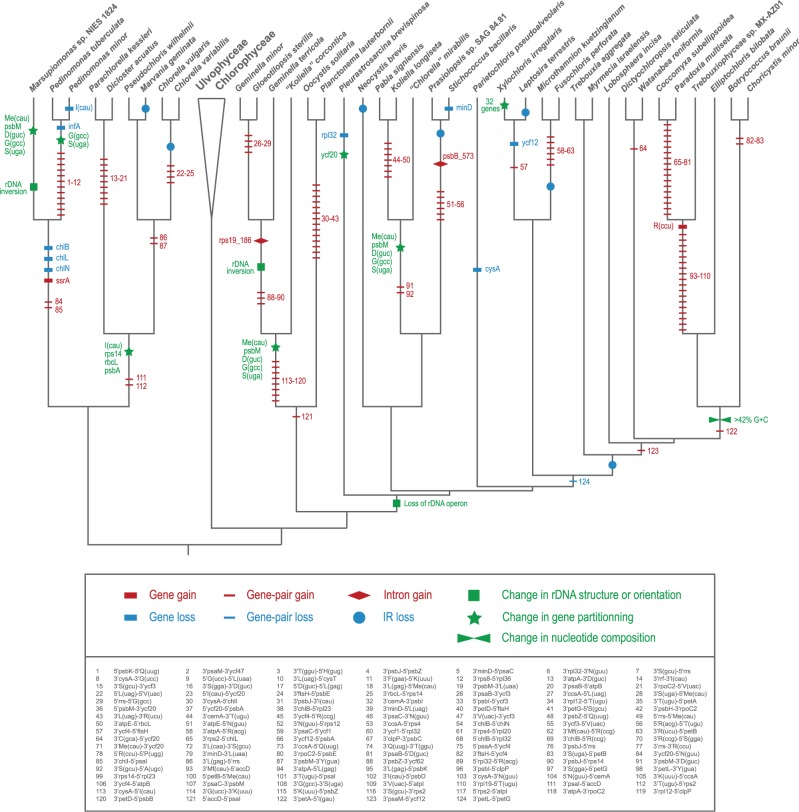
Inferred gains and losses of chloroplast genomic features during the evolution of trebouxiophyceans. Note that conserved gene pairs could not be inferred for the *T. aggregata* chloroplast genome because the sequence of this genome is partial and fragmented on 41 contigs. The gene pairs corresponding to the numbered characters are listed at the bottom of the figure.

Considering that six of the eight previously analyzed trebouxiophycean chloroplast genomes featured no large IR (table 1), it was unclear how many times this structure was lost in the Trebouxiophyceae. Here, we found that all three pedinophyceans but less than half of the trebouxiophyceans (15 of 35) feature a large IR that partly encodes the rRNA genes (fig. 1). Mapping of the IR presence/absence on the phylogeny inferred by Lemieux et al. (2014a) reveals seven independent losses of this structure: Two losses in the Chlorellales, two in the Prasiolales, one in the Microthamniales, one in the lineage leading *Leptosira*, and one in the superclade containing the Trebouxiales, *Lobosphaera*, *Watanabea*, *Choricystis* and *Elliptochloris* lineages (fig. 8). Considering that the only IR-containing genome in the clade sister to the *Parieotochloris* lineage (the *X. irregularis* cpDNA) is very atypical in its gene partitioning pattern (with each SC region containing a number of genes ancestrally located in the SSC and LSC), one could speculate that the *Xylochloris* IR arose de novo. According to this hypothesis, the abovementioned superclade demarcated by an IR loss would also comprise the Microthamniales and *Xylochloris* lineages and five IR losses would be required to account for the phylogeny inferred by Lemieux et al. (2014a). This evolutionary scenario may seem unlikely because an IR loss and replacement has not been documented in any viridiplant chloroplast genome. The de novo creation of an IR, however, may also be invoked to account for the unusual 8.3-kb IR that contains no rRNA genes in the prasiolalean *S. bacillaris*, although the possibility that it represents the remnant of a bona fide rDNA-encoding IR cannot be excluded. To the best of our knowledge, only the plastid of the nonphotosynthetic orchid *Rhizanthella gardneri* has been reported to carry a reduced IR lacking any rRNA genes (Delannoy et al. 2011).

The chloroplast genome also sustained independent losses of the IR in other major groups of green algae as well as in land plants (Jansen and Ruhlman 2012; Ruhlman and Jansen 2014). This structure has been lost at least four times in prasinophyceans (Turmel, Gagnon, et al. 2009; Lemieux et al. 2014b), three times in zygnematalean streptophytes (Turmel et al. 2005; Civan et al. 2014), once in the Chlorophyceae (Brouard et al. 2010), and twice in the Ulvophyceae (Lu et al. 2011; Leliaert and Lopez-Bautista 2015; Melton et al. 2015). In the case of the Chlorophyceae, the reported loss unites two major lineages of the OCC clade (Chaetophorales and Chaetopeltidales). Broader taxon sampling of each of these major groups is expected to uncover additional events of IR loss.

### Dynamic Evolution of the IR

The IR ranges from approximately 7 to 28 kb in most of the 15 trebouxiophyceans carrying this structure and reaches 45 kb in the core trebouxiophycean *P. brevispinosa* (fig. 2). As previously mentioned, in all species but *X. irregularis*, the IR divides the genome into SSC and LSC regions whose gene contents are similar to those found in the chloroplasts of most prasinophycean algae and streptophytes. The main differences we observed relative to the ancestral gene partitioning pattern are mainly accounted for by the relocalization of a few genes ancestrally present in the LSC region to the IR or immediately adjacent SSC sequence. The IRs of both the pedinophyceans and core trebouxiophyceans belonging to the *Oocystis*/*Geminella* clade include the same set of five relocalized genes (*psbM*, *trnS*(uga), *trnD*(guc), *trnMe*(cau), and *trnG*(gcc)) (fig. 4), but in the Prasiolales and the *Parietochloris pseudoalveolaris* lineage, most if not all of these five genes were relocated to the SSC region as a result of IR contractions and were subsequently dispersed through gene reversals. The large IR of *P. brevispinosa* includes four additional genes that are present in the LSC in most prasinophycean and streptophyte cpDNAs. Notably, the gene partitioning pattern observed in the Chlorellales is unique in exhibiting in the IR and/or SSC, a different set of reshuffled genes (*trnI*(cau), *rps14*, *rbcL,* and *psbA*). Compared with the genomes examined in our study, the only two IR-containing genomes currently available for the Ulvophyceae also differ from the ancestral partitioning pattern but show more extensive gene transfers from the LSC to the SSC region (Pombert et al. 2005, 2006). In the Chlorophyceae, restructuring of the chloroplast genome appears to be particularly dynamic, involving frequent transfers from both LSC to SSC and SSC to LSC. The minor deviations from the ancestral gene partitioning pattern that we report for the Pedinophyceae and Trebouxiophyceae are consistent with the notion that these classes represent deeper branches than the Ulvophyceae and Chlorophyceae (Lemieux et al. 2014a).

Events of IR expansion and contraction occurred frequently through evolutionary time in both the Pedinophyceae and Trebouxiophyceae, leading not only to changes in gene content but also to gene rearrangements and gene duplications. We observed IR boundary shifts in all lineages in which more than one IR-containing genome was investigated. In the Chlorellales, each of the three examined genomes displays unique IR/LSC and IR/SSC junctions, implying boundary shifts toward the IR or outward on both the SSC and LSC sides of the IR. Movement of the IR is thought to be caused by inversions or double-strand DNA break repairs (Goulding et al. 1996; Wang et al. 2008) and small-scale changes have been identified for most of the land plants investigated so far although the highly rearranged chloroplast genomes in the family Geraniaceae appear to have undergone much larger IR expansions and contractions due to impaired recombination (Guisinger et al. 2011; Weng et al. 2014). Because taxon sampling in our study is too broad and also because the IR is subject to frequent gene rearrangements and insertion/deletion events, our data do not allow us to accurately predict neither the number of IR expansions and contractions nor the mechanisms underlying these events. In some cases, multiple steps of IR boundary shifts are required to explain our observations. For instance, the differences between the *Koliella longiseta* and “*Chlorella” mirabilis* genomes at the IR/LSC boundary are attributable to a minimum of two steps: The first involving *trnS*(gcu) and the other *psbA* (fig. 4). The latter genes are adjacent in the *K. longiseta* IR but lie at opposite ends of the LSC region in the *“Chlorella” mirabilis* genome. In the Pedinophyceae, small-scale IR boundary shifts encompassing a few hundreds base pairs caused no apparent change in gene content but led to partial duplications of *atpA* at the LSC/IR junction of *Marsupiomonas* sp*.* and of *cysT* at the SSC/IR junction of *Pedinomonas tuberculata* (fig. 2).

Gene rearrangements in the IR also took place through sequence inversions in the Pedinophyceae and *Geminella* clade (figs. 2 and 4). The multiple gene rearrangements distinguishing the IR of *Marsupiomonas* sp. from those of the two *Pedinomonas* taxa include the inversion of the entire rDNA operon. As observed in most prasinophycean and streptophyte IR-containing cpDNAs, the rDNA operon is transcribed toward the SSC region in the *Pedinomonas* species. Reversal of the rDNA operon also occurred during the evolutionary span separating *“Koliella” corcontica* from the three other members of the *Geminella* clade (fig. 8). Furthermore, the rDNA operon was disrupted at multiple sites in later diverging lineages of core trebouxiophyceans, likely starting with the very early breakage of the intergenic region between *rrs* and *trnI*(gau) (fig. 8 and supplementary fig. S11, Supplementary Material online). Given the complexity of the associated rearrangements, it is challenging to reconstruct the series of inversions that led to the various configurations observed for the rRNA and tRNA genes composing the operon. Obviously, these events were accompanied by the gains of new promoters in order to enable the expression of the relocated genes. Disruptions of the chloroplast rDNA operon have been reported for other viridiplant lineages, especially for highly rearranged genomes lacking an IR (Turmel et al. 2005; Turmel, Gagnon, et al. 2009; Guisinger et al. 2011; Lu et al. 2011; Civan et al. 2014; Lemieux et al. 2014b; Leliaert and Lopez-Bautista 2015). As in the evolutionary scenario that has been inferred for the Geraniaceae (Weng et al. 2014), a series of IR contractions and expansions as well as inversions might have occurred in a number of green algal lineages, leading to complete loss of the IR and/or breakage of the rDNA operon.

### Variable Levels of Chloroplast Genome Rearrangements across Lineages

Land plant and green algal chloroplast genomes lacking an IR are usually more rearranged than their IR-containing homologs (Turmel et al. 2005; de Cambiaire et al. 2007; Turmel, Gagnon, et al. 2009; Brouard et al. 2010; Lemieux et al. 2014b; Ruhlman and Jansen 2014). The genomic rearrangement tree reported here suggests that gene order in most of the trebouxiophycean IR-less genomes was also reconfigured at a faster rate compared with their IR-containing homologs (fig. 5). In the superclade characterized by an IR loss, several ancestral operons of cyanobacterial origin (*petA, psbB, psbE, rpoB*, rDNA, and ribosomal protein operons) were disrupted (fig. 6) and a few genes (*rpoB, rpoC2,* and *tilS*) were fragmented (supplementary fig. S3, Supplementary Material online). We cannot conclude, however, that IR loss is the main force driving genome destabilization because three independent trebouxiophycean lineages with IR-less genomes (*Myrmecia israelensis*, *N. brevis,* and some chlorellalean lineages) feature short branches in the rearrangement tree and retain the ancestral gene content typical of the SSC partition (supplementary fig. S13, Supplementary Material online). Interestingly, the lineages with a faster rate of genome rearrangements tend to show an accelerated rate of sequence evolution (fig. 5 and supplementary fig. S9, Supplementary Material online). Increased genome rearrangements have also been correlated with accelerated substitution rates in the mitochondria of insects (Shao et al. 2003) and arthropods (Xu et al. 2006), and in the chloroplasts of seed plants belonging to the Geraniaceae (Weng et al. 2014).

Inversions caused by recombination between repeated sequences are thought to be the main mechanism for gene shuffling in chloroplast genomes (Palmer 1991; Jansen and Ruhlman 2012). In the Geraniaceae, the extent of chloroplast genome rearrangements has been correlated with the proportion and numbers of repeated sequences, a significant portion of which consists of fully or partially duplicated gene sequences (Weng et al. 2014). In our study, we observed a very irregular distribution of repeat-rich genomes on the phylogenetic tree (fig. 1 and supplementary fig. S2, Supplementary Material online) and found no strict correlation between the proportion/sizes of repeats and the degree of gene rearrangements (figs. 1 and 5, and supplementary fig. S2, Supplementary Material online). Moreover, in contrast to the situation observed in land plants, the majority of repeats identified here and in previously studied green algal cpDNAs has no similarity with gene-coding regions. We identified at least three cases where sister taxa differ considerably in the proportion of repeats but are similar in their level of genomic rearrangements (*Geminella terricola* and *Geminella minor*, *Pa. signiensis* and *K. longiseta*, *Prasiolopsis* sp. SAG 84.81 and *S. bacillaris*). Though we cannot eliminate the possibility that repeats have promoted or have been associated in some way with changes in chloroplast gene order during the evolution of the Trebouxiophyceae, their rapid evolution might have obscured or eliminated signals of past rearrangement events in which they participated. Alternatively, as breakpoints of genome rearrangements have been associated with tRNA genes in viridiplant genomes, homologous recombination between tRNA genes might have lead to gene inversions in these genomes (Hiratsuka et al. 1989; Turmel et al. 2002; Haberle et al. 2008).

### A Trebouxiophycean Clade with G+C-Biased Chloroplast Genomes

We found that all six representatives of a highly derived monophyletic group within the IR-less superclade of core trebouxiophyceans (the *Elliptochloris*/*Choricystis* clade) display a G+C-biased nucleotide composition in their chloroplast genome (G+C contents of 42–58%, table 1). This trait is unusual considering that the vast majority of available viridiplant chloroplast genomes is A+T-biased possibly due to selection for translational efficiency and to AT mutation pressure coupled with inefficient DNA repair systems (Lynch 2007). In the *Elliptochloris*/*Choricystis* clade, protein-coding genes show a greater range of variation in G+C content at third codon positions compared with the more functionally constrained first and second codon positions (supplementary fig. S5, Supplementary Material online). This observation is consistent with the analyses of nucleotide composition previously reported for two free-living green algae belonging to this clade, *Co. subellipsoidea* (Smith et al. 2011) and Trebouxiophyceae sp. MX-AZ01 (Servin-Garciduenas and Martinez-Romero 2012). Interestingly, the mitochondrial and nuclear genomes of *Co. subellipsoidea* (Smith et al. 2011) and the mitochondrial genome of Trebouxiophyceae sp. MX-AZ01 (Servin-Garciduenas and Martinez-Romero 2012) have also high levels of G and C. It has been argued that the forces driving the nucleotide composition toward G and C in both organelles of *Co. subellipsoidea* are neutral and linked to a nuclear mutation affecting GC-biased gene conversion or cell-wide features (e.g., life history-related traits or metabolic features) (Smith et al. 2011). In this context, it is worth mentioning that the chloroplast *rbcL* gene has been found to be richer in A+T in symbiotic species of *Coccomyxa* than in free-living species (Smith et al. 2011). It will be interesting to see whether the mitochondrial and nuclear genomes of the other members of the *Elliptochloris*/*Choricystis* clade have also G+C-biased nucleotide compositions.

### Acquisitions of Foreign Genes

Prior to our investigation, gains of foreign genes by organelle genomes had been documented for several green algal lineages: Prasinophyceae (Turmel, Gagnon, et al. 2009; Lemieux et al. 2014b), Ulvophyceae (Leliaert and Lopez-Bautista 2015), Chlorophyceae (Brouard et al. 2008), and streptophyte algae (Turmel et al. 2002, 2013). Here, we report evidence suggesting that bacterial/viral genes were transferred to the chloroplast multiple times during the evolution of pedinophyceans and trebouxiophyceans. As observed previously, all the sequences associated with these putative events encode proteins acting on DNA or RNA, and most have homologs in other green algal chloroplasts and/or mitochondria. These sequences consist of all the ORFs listed in table 2, with the exception of the five ORFs encoding LAGLIDADG homing endonucleases and reverse transcriptases; they code for DNA breaking–rejoining enzymes, integrases, primases, and type-B DNA polymerases (table 2). To our surprise, we found putative restriction endonuclease genes in *Pe. tuberculata* and *Microthamnion kuetzingianum*, an N-6 DNA methylase gene possibly associated with a bacterial restriction/modification system in *C. variabilis*, a desoxyribonucleotide kinase gene of viral origin in *M. germinata*, and a bacterial serine recombinase/resolvase in *Dictyochloropsis reticulans*. This is the first time that these unusual coding sequences are observed in green algal chloroplast genomes; however, the presence of bacterial C-5 DNA methylase genes, which are also associated with restriction/modification systems, has been recently documented in the chloroplast genome of the ulvophycean *Tydemania expeditiones* (Leliaert and Lopez-Bautista 2015) and the mitochondrial genome of the charophycean *Klebsormidium* sp. (Turmel et al. 2013). Considering the substantial proportion of trebouxiophycean taxa in which we detected ORFs of putative bacterial and viral origins together with the fact that the identified ORFs are not conserved in members of the same lineage, it is plausible that the chloroplast genome participated in frequent events of horizontal gene transfer during the evolution of the Trebouxiophyceae and that the captured sequences were not conserved over long evolutionary periods because they conferred no selective advantage. In this regard, it is intriguing that three members of the *Prasiola* clade, including the early diverging *N. brevis*, have maintained an hypothetical gene found in the marine γ-proteobacterium *Marinobacter manganoxydans* (Alteromonadales) and closely related marine bacteria (supplementary fig. S6, Supplementary Material online). It is worth mentioning here that many strains of this genus have the ability to interact with marine algae and plankton (Amin et al. 2012); their close association with a marine ancestor of the Prasiolales might have facilitated gene transfer to the chloroplast.

The ORFs encoding putative LAGLIDADG homing endonucleases and reverse transcriptases are more likely to be remnants of group I and group II introns that were originally present in standard chloroplast genes. These intron sequences might have found their way into intergenic regions through a variety of processes, including intragenomic proliferation of mobile introns, degeneration of a duplicated intron-containing gene, genomic rearrangement or horizontal transfer of mobile introns. Interestingly, two of the three trebouxiophycean cpDNAs carrying a free-standing LAGLIDADG homing endonuclease gene also feature standard genes interrupted by LAGLIDADG endonuclease-encoding group I introns, whereas both trebouxiophycean cpDNAs carrying a free-standing reverse transcriptase gene also possess standard genes with reverse transcriptase-encoding group II introns (table 2 and fig. 7).

### Reconstruction of Chloroplast Genomic Changes and Its Impact on Previously Inferred Phylogenetic Relationships

Investigation of structural changes in organelle genomes may complement phylogenetic analyses by reinforcing observed relationships and helping to resolve phylogenetic issues. The chloroplast genome analyses presented here, in particular those based on gene order, support several of the relationships observed in the phylogenies inferred from sequence data (fig. 8 and supplementary fig. S10, Supplementary Material online). However, because of the highly dynamic evolution of the chloroplast genome, structural genomic characters provide little information on higher order relationships within the Trebouxiophyceae and on the monophyletic/nonmonophyletic status of this class.

As pointed out by Lemieux et al. (2014a), the question as to whether the Chlorellales and Pedinophyceae form a monophyletic group remains unsettled. It is possible that the affiliation of these lineages in phylogenomic analyses of chloroplast genes and proteins is the result of systematic errors of phylogenetic reconstructions arising from model misspecification (Lemieux et al. 2014a). In this context, it is interesting to note that the tree we inferred here from gene order data did not recover the Chlorellales as sister to the Pedinophyceae, placing instead this lineage as sister to the *Geminella* clade with low bootstrap support (supplementary fig. S10, Supplementary Material online). Consistent with this observation, our reconstruction of chloroplast genomic changes revealed no synapomorphic character uniting the Chlorellales and Pedinomonadales (fig. 8). The unique partitioning pattern observed for the Chlorellales clearly indicates that the chloroplast genome followed a distinctive evolutionary pathway in this algal group. Phylogenomic studies using a broader sampling of chlorophytes that include the Chlorodendrophyceae, additional ulvophycean taxa, and representatives of the prasinophyte CCMP 1205 lineage will be needed to resolve the ancient and rapid radiations of core chlorophytes.

## Conclusions

Despite the remarkable diversity of the Trebouxiophyceae, surprisingly little was known about the chloroplast genomes of this class. To fill this gap, we have tripled the number of fully sequenced chloroplast genomes and expanded the phylogenetic breadth of the sampled taxa. The 35 trebouxiophycean and three pedinophycean genomes compared in this study display considerable variability at all levels, except standard gene content. Our study highlights the highly dynamic nature of the trebouxiophycean chloroplast genome, in particular with regards to the large IR sequence that experienced repeated losses and extensive changes in size, gene content, and gene order during evolution. Of the seven predicted IR losses, one event demarcates a superclade of 11 taxa representing five late-diverging lineages. Several ancestral operons were disrupted in this superclade, a few genes were fragmented, and a subgroup of taxa gained a G+C-biased nucleotide composition. The broad taxon sampling used in our investigation prevented us from deciphering the mechanisms and forces underlying the observed IR losses, large IR expansions/contractions, and gene rearrangements. Studies on other viridiplant lineages, mainly land plants, suggest that a diversity of nonadaptive forces influence chloroplast genome architecture and that these forces differ among lineages (Smith et al. 2013; Knox 2014; Weng et al. 2014; Wu and Chaw 2014). To advance our understanding of the dynamic history of the chloroplast genome in the Trebouxiophyceae, it will be necessary to analyze additional taxa from the lineages that display the most extensive genomic changes.

## Supplementary Material

Supplementary figures S1–S13 are available at *Genome Biology and Evolution* online (http://www.gbe.oxfordjournals.org/).

Supplementary Data

## References

[evv130-B1] Amin SA Green DH Al Waheeb D Gardes A Carrano CJ 2012 Iron transport in the genus *Marinobacter*. Biometals 25:135–147. 21894542 10.1007/s10534-011-9491-9

[evv130-B2] Bao Z Eddy SR 2002 Automated *de novo* identification of repeat sequence families in sequenced genomes. Genome Res. 12:1269–1276. 12176934 10.1101/gr.88502 PMC186642

[evv130-B3] Bélanger AS 2006 Distinctive architecture of the chloroplast genome in the chlorophycean green alga *Stigeoclonium helveticum*. Mol Genet Genomics. 276:464–477. 16944205 10.1007/s00438-006-0156-2

[evv130-B4] Bourque G Pevzner PA 2002 Genome-scale evolution: reconstructing gene orders in the ancestral species. Genome Res. 12:26–36. 11779828 PMC155248

[evv130-B5] Brouard JS Otis C Lemieux C Turmel M 2008 Chloroplast DNA sequence of the green alga *Oedogonium cardiacum* (Chlorophyceae): unique genome architecture, derived characters shared with the Chaetophorales and novel genes acquired through horizontal transfer. BMC Genomics 9:290. 18558012 10.1186/1471-2164-9-290 PMC2442088

[evv130-B6] Brouard JS Otis C Lemieux C Turmel M 2010 The exceptionally large chloroplast genome of the green alga *Floydiella terrestris* illuminates the evolutionary history of the Chlorophyceae. Genome Biol Evol. 2:240–256. 20624729 10.1093/gbe/evq014 PMC2997540

[evv130-B7] Brouard JS Otis C Lemieux C Turmel M 2011 The chloroplast genome of the green alga *Schizomeris leibleinii* (Chlorophyceae) provides evidence for bidirectional DNA replication from a single origin in the chaetophorales. Genome Biol Evol. 3:505–515. 21546564 10.1093/gbe/evr037 PMC3138424

[evv130-B8] Civan P Foster PG Embley MT Seneca A Cox CJ 2014 Analyses of charophyte chloroplast genomes help characterize the ancestral chloroplast genome of land plants. Genome Biol Evol. 6:897–911. 24682153 10.1093/gbe/evu061 PMC4007539

[evv130-B9] Cui L 2006 Adaptive evolution of chloroplast genome structure inferred using a parametric bootstrap approach. BMC Evol Biol. 6:13. 16469102 10.1186/1471-2148-6-13 PMC1421436

[evv130-B10] Darling AE Mau B Perna NT 2010 progressiveMauve: multiple genome alignment with gene gain, loss and rearrangement. PLoS One 5:e11147. 20593022 10.1371/journal.pone.0011147 PMC2892488

[evv130-B11] de Cambiaire JC Otis C Lemieux C Turmel M 2006 The complete chloroplast genome sequence of the chlorophycean green alga *Scenedesmus obliquus* reveals a compact gene organization and a biased distribution of genes on the two DNA strands. BMC Evol Biol. 6:37. 16638149 10.1186/1471-2148-6-37 PMC1513399

[evv130-B12] de Cambiaire JC Otis C Turmel M Lemieux C 2007 The chloroplast genome sequence of the green alga *Leptosira terrestris*: multiple losses of the inverted repeat and extensive genome rearrangements within the Trebouxiophyceae. BMC Genomics 8:213. 17610731 10.1186/1471-2164-8-213 PMC1931444

[evv130-B13] Delannoy E Fujii S Colas des Francs-Small C Brundrett M Small I 2011 Rampant gene loss in the underground orchid *Rhizanthella gardneri* highlights evolutionary constraints on plastid genomes. Mol Biol Evol. 28:2077–2086. 21289370 10.1093/molbev/msr028 PMC3112369

[evv130-B14] Friedl T Rybalka N 2012 Systematics of the green algae: a brief introduction to the current status. In: Luttge U Beyschlag W Budel B Francis D, editors. Progress in botany. vol. 73 Berlin (Germany): Springer-Verlag p. 259–280.

[evv130-B15] Fucikova K 2014 New phylogenetic hypotheses for the core Chlorophyta based on chloroplast sequence data. Front Ecol Evol. 2:63.

[evv130-B16] Goulding SE Olmstead RG Morden CW Wolfe KH 1996 Ebb and flow of the chloroplast inverted repeat. Mol Gen Genet. 252:195–206. 8804393 10.1007/BF02173220

[evv130-B17] Gray MW Archibald JM 2012 Origins of mitochondria and plastids. In: Bock R Knoop V, editors. Genomics of chloroplasts and mitochondria. Dordrecht (The Netherlands): Springer. p. 1-30.

[evv130-B18] Green BR 2011 Chloroplast genomes of photosynthetic eukaryotes. Plant J. 66:34–44. 21443621 10.1111/j.1365-313X.2011.04541.x

[evv130-B19] Gueneau de Novoa P Williams KP 2004 The tmRNA website: reductive evolution of tmRNA in plastids and other endosymbionts. Nucleic Acids Res. 32:D104–D108. 14681369 10.1093/nar/gkh102 PMC308836

[evv130-B20] Guisinger MM Kuehl JV Boore JL Jansen RK 2011 Extreme reconfiguration of plastid genomes in the angiosperm family Geraniaceae: rearrangements, repeats, and codon usage. Mol Biol Evol. 28:583–600. 20805190 10.1093/molbev/msq229

[evv130-B21] Haberle RC Fourcade HM Boore JL Jansen RK 2008 Extensive rearrangements in the chloroplast genome of *Trachelium caeruleum* are associated with repeats and tRNA genes. J Mol Evol. 66:350–361. 18330485 10.1007/s00239-008-9086-4

[evv130-B22] Hiratsuka J 1989 The complete sequence of the rice (*Oryza sativa*) chloroplast genome: intermolecular recombination between distinct tRNA genes accounts for a major plastid DNA inversion during the evolution of the cereals. Mol Gen Genet. 217:185–194. 2770692 10.1007/BF02464880

[evv130-B23] Hu F Lin Y Tang J 2014 MLGO: phylogeny reconstruction and ancestral inference from gene-order data. BMC Bioinformatics 15:354. 25376663 10.1186/s12859-014-0354-6 PMC4236499

[evv130-B24] Jansen RK Ruhlman TA 2012 Plastid genomes of seed plants. In: Bock R Knoop V, editors. Genomics of chloroplasts and mitochondria. Dordrecht (The Netherlands): Springer. p. 103-126.

[evv130-B25] Kashino Y 2007 Ycf12 is a core subunit in the photosystem II complex. Biochim Biophys Acta. 1767:1269–1275. 17935689 10.1016/j.bbabio.2007.08.008

[evv130-B26] Kittichotirat W Bumgarner R Chen C 2010 Markedly different genome arrangements between serotype a strains and serotypes b or c strains of *Aggregatibacter actinomycetemcomitans*. BMC Genomics 11:489. 20825670 10.1186/1471-2164-11-489 PMC2996985

[evv130-B27] Knox EB 2014 The dynamic history of plastid genomes in the Campanulaceae *sensu lato* is unique among angiosperms. Proc Natl Acad Sci U S A. 111:11097–11102. 25024223 10.1073/pnas.1403363111 PMC4121848

[evv130-B28] Kurtz S 2001 REPuter: the manifold applications of repeat analysis on a genomic scale. Nucleic Acids Res. 29:4633–4642. 11713313 10.1093/nar/29.22.4633 PMC92531

[evv130-B29] Lambowitz AM Zimmerly S 2011 Group II introns: mobile ribozymes that invade DNA. Cold Spring Harb Perspect Biol. 3:a003616. 20463000 10.1101/cshperspect.a003616 PMC3140690

[evv130-B30] Lang BF Nedelcu AM 2012 Plastid genomes of algae. In: Bock R Knoop V, editors. Genomics of chloroplasts and mitochondria. Dordrecht (The Netherlands): Springer. p. 59-87.

[evv130-B31] Leliaert F 2012 Phylogeny and molecular evolution of the green algae. CRC Crit Rev Plant Sci. 31:1–46.

[evv130-B32] Leliaert F Lopez-Bautista JM 2015 The chloroplast genomes of *Bryopsis plumosa* and *Tydemania expeditiones* (Bryopsidales, Chlorophyta): compact genomes and genes of bacterial origin. BMC Genomics 16:204. 25879186 10.1186/s12864-015-1418-3 PMC4487195

[evv130-B33] Lemieux C Otis C Turmel M 2014a Chloroplast phylogenomic analysis resolves deep-level relationships within the green algal class Trebouxiophyceae. BMC Evol Biol. 14:211. 25270575 10.1186/s12862-014-0211-2 PMC4189289

[evv130-B34] Lemieux C Otis C Turmel M 2014b Six newly sequenced chloroplast genomes from prasinophyte green algae provide insights into the relationships among prasinophyte lineages and the diversity of streamlined genome architecture in picoplanktonic species. BMC Genomics 15:857. 25281016 10.1186/1471-2164-15-857 PMC4194372

[evv130-B35] Lewis LA McCourt RM 2004 Green algae and the origin of land plants. Am J Bot. 91:1535–1556. 21652308 10.3732/ajb.91.10.1535

[evv130-B36] Lohse M Drechsel O Bock R 2007 OrganellarGenomeDRAW (OGDRAW): a tool for the easy generation of high-quality custom graphical maps of plastid and mitochondrial genomes. Curr Genet. 52:267–274. 17957369 10.1007/s00294-007-0161-y

[evv130-B37] Lu F 2011 The *Bryopsis hypnoides* plastid genome: multimeric forms and complete nucleotide sequence. PLoS One 6:e14663. 21339817 10.1371/journal.pone.0014663 PMC3038852

[evv130-B38] Lynch M 2007 The origins of genome architecture. Sunderland (MA): Sinauer Associates.

[evv130-B39] Maddison DR Maddison WP 2000 MacClade 4: analysis of phylogeny and character evolution. Sunderland (MA): Sinauer Associates. 10.1159/000156416 2606395

[evv130-B40] Maddison WP Maddison DR 2015 Mesquite: a modular system for evolutionary analysis. Version 3.02. Available from: http://mesquiteproject.org

[evv130-B41] Marin B 2012 Nested in the Chlorellales or independent class? Phylogeny and classification of the Pedinophyceae (Viridiplantae) revealed by molecular phylogenetic analyses of complete nuclear and plastid-encoded rRNA operons. Protist 163:778–805. 22192529 10.1016/j.protis.2011.11.004

[evv130-B42] Maul JE 2002 The *Chlamydomonas reinhardtii* plastid chromosome: islands of genes in a sea of repeats. Plant Cell 14:2659–2679. 12417694 10.1105/tpc.006155 PMC153795

[evv130-B43] Melton JT 3rd Leliaert F Tronholm A Lopez-Bautista JM 2015 The complete chloroplast and mitochondrial genomes of the green macroalga *Ulva* sp. UNA00071828 (Ulvophyceae, Chlorophyta). PLoS One 10:e0121020. 25849557 10.1371/journal.pone.0121020 PMC4388391

[evv130-B44] Michel F Umesono K Ozeki H 1989 Comparative and functional anatomy of group II catalytic introns—a review. Gene 82:5–30. 2684776 10.1016/0378-1119(89)90026-7

[evv130-B45] Michel F Westhof E 1990 Modelling of the three-dimensional architecture of group I catalytic introns based on comparative sequence analysis. J Mol Biol. 216:585–610. 2258934 10.1016/0022-2836(90)90386-Z

[evv130-B46] Palmer JD 1991 Plastid chromosomes: structure and evolution. In: Vasil IK, editor. The molecular biology of plastids. San Diego (CA): Academic Press p. 5–53.

[evv130-B47] Palmer JD 2003 The symbiotic birth and spread of plastids: how many times and whodunit? J Phycol. 39:4–12.

[evv130-B48] Pombert JF Lemieux C Turmel M 2006 The complete chloroplast DNA sequence of the green alga *Oltmannsiellopsis viridis* reveals a distinctive quadripartite architecture in the chloroplast genome of early diverging ulvophytes. BMC Biol. 4:3. 16472375 10.1186/1741-7007-4-3 PMC1402334

[evv130-B49] Pombert JF Otis C Lemieux C Turmel M 2005 The chloroplast genome sequence of the green alga *Pseudendoclonium akinetum* (Ulvophyceae) reveals unusual structural features and new insights into the branching order of chlorophyte lineages. Mol Biol Evol. 22:1903–1918. 15930151 10.1093/molbev/msi182

[evv130-B50] Rice P Longden I Bleasby A 2000 EMBOSS: the European molecular biology open software suite. Trends Genet. 16:276–277. 10827456 10.1016/s0168-9525(00)02024-2

[evv130-B51] Robbens S 2007 The complete chloroplast and mitochondrial DNA sequence of *Ostreococcus tauri*: organelle genomes of the smallest eukaryote are examples of compaction. Mol Biol Evol. 24:956–968. 17251180 10.1093/molbev/msm012

[evv130-B52] Rodriguez-Ezpeleta N 2005 Monophyly of primary photosynthetic eukaryotes: green plants, red algae, and glaucophytes. Curr Biol. 15:1325–1330. 16051178 10.1016/j.cub.2005.06.040

[evv130-B53] Ruhlman T Jansen R 2014 The plastid genomes of flowering plants. In: Maliga P, editor. Chloroplast biotechnology. New York: Humana Press p. 3–38.

[evv130-B54] Servin-Garciduenas LE Martinez-Romero E 2012 Complete mitochondrial and plastid genomes of the green microalga *Trebouxiophyceae* sp. strain MX-AZ01 isolated from a highly acidic geothermal lake. Eukaryot Cell. 11:1417–1418. 23104370 10.1128/EC.00244-12 PMC3486031

[evv130-B55] Shao R Dowton M Murrell A Barker SC 2003 Rates of gene rearrangement and nucleotide substitution are correlated in the mitochondrial genomes of insects. Mol Biol Evol. 20:1612–1619. 12832626 10.1093/molbev/msg176

[evv130-B56] Smith DR 2010 The *Dunaliella salina* organelle genomes: large sequences, inflated with intronic and intergenic DNA. BMC Plant Biol. 10:83. 20459666 10.1186/1471-2229-10-83 PMC3017802

[evv130-B57] Smith DR 2011 The GC-rich mitochondrial and plastid genomes of the green alga *Coccomyxa* give insight into the evolution of organelle DNA nucleotide landscape. PLoS One 6:e23624. 21887287 10.1371/journal.pone.0023624 PMC3162594

[evv130-B58] Smith DR 2013 Organelle genome complexity scales positively with organism size in volvocine green algae. Mol Biol Evol. 30:793–797. 23300255 10.1093/molbev/mst002

[evv130-B59] Smith DR Lee RW 2009 The mitochondrial and plastid genomes of *Volvox carteri*: bloated molecules rich in repetitive DNA. BMC Genomics 10:132. 19323823 10.1186/1471-2164-10-132 PMC2670323

[evv130-B60] Soria-Carrasco V Talavera G Igea J Castresana J 2007 The K tree score: quantification of differences in the relative branch length and topology of phylogenetic trees. Bioinformatics 23:2954–2956. 17890735 10.1093/bioinformatics/btm466

[evv130-B61] Stoddard BL 2014 Homing endonucleases from mobile group I introns: discovery to genome engineering. Mob DNA. 5:7. 24589358 10.1186/1759-8753-5-7 PMC3943268

[evv130-B62] Tesler G 2002 GRIMM: genome rearrangements web server. Bioinformatics 18:492–493. 11934753 10.1093/bioinformatics/18.3.492

[evv130-B63] Turmel M Brouard JS Gagnon C Otis C Lemieux C 2008 Deep division in the Chlorophyceae (Chlorophyta) revealed by chloroplast phylogenomic analyses. J Phycol. 44:739–750. 10.1111/j.1529-8817.2008.00510.x 27041432

[evv130-B64] Turmel M Gagnon MC O’Kelly CJ Otis C Lemieux C 2009 The chloroplast genomes of the green algae *Pyramimonas, Monomastix*, and *Pycnococcus* shed new light on the evolutionary history of prasinophytes and the origin of the secondary chloroplasts of euglenids. Mol Biol Evol. 26:631–648. 19074760 10.1093/molbev/msn285

[evv130-B65] Turmel M Otis C Lemieux C 2002 The chloroplast and mitochondrial genome sequences of the charophyte *Chaetosphaeridium globosum*: insights into the timing of the events that restructured organelle DNAs within the green algal lineage that led to land plants. Proc Natl Acad Sci U S A. 99:11275–11280. 12161560 10.1073/pnas.162203299 PMC123247

[evv130-B66] Turmel M Otis C Lemieux C 2005 The complete chloroplast DNA sequences of the charophycean green algae *Staurastrum* and *Zygnema* reveal that the chloroplast genome underwent extensive changes during the evolution of the Zygnematales. BMC Biol. 3:22. 16236178 10.1186/1741-7007-3-22 PMC1277820

[evv130-B67] Turmel M Otis C Lemieux C 2009 The chloroplast genomes of the green algae *Pedinomonas minor, Parachlorella kessleri*, and *Oocystis solitaria* reveal a shared ancestry between the Pedinomonadales and Chlorellales. Mol Biol Evol. 26:2317–2331. 19578159 10.1093/molbev/msp138

[evv130-B68] Turmel M Otis C Lemieux C 2013 Tracing the evolution of streptophyte algae and their mitochondrial genome. Genome Biol Evol. 5:1817–1835. 24022472 10.1093/gbe/evt135 PMC3814193

[evv130-B69] Turmel M Pombert JF Charlebois P Otis C Lemieux C 2007 The green algal ancestry of land plants as revealed by the chloroplast genome. Int J Plant Sci. 168:679–689.

[evv130-B70] Wakasugi T 1997 Complete nucleotide sequence of the chloroplast genome from the green alga *Chlorella vulgaris*: the existence of genes possibly involved in chloroplast division. Proc Natl Acad Sci U S A. 94:5967–5972. 9159184 10.1073/pnas.94.11.5967 PMC20890

[evv130-B71] Wang RJ 2008 Dynamics and evolution of the inverted repeat-large single copy junctions in the chloroplast genomes of monocots. BMC Evol Biol. 8:36. 18237435 10.1186/1471-2148-8-36 PMC2275221

[evv130-B72] Weng ML Blazier JC Govindu M Jansen RK 2014 Reconstruction of the ancestral plastid genome in Geraniaceae reveals a correlation between genome rearrangements, repeats, and nucleotide substitution rates. Mol Biol Evol. 31:645–659. 24336877 10.1093/molbev/mst257

[evv130-B73] Wolf PG Karol KG 2012 Plastomes of bryophytes, lycophytes and ferns. In: Bock R Knoop V, editors. Genomics of chloroplasts and mitochondria. Dordrecht (The Netherlands): Springer. p. 89-102.

[evv130-B74] Wu CS Chaw SM 2014 Highly rearranged and size-variable chloroplast genomes in conifers II clade (cupressophytes): evolution towards shorter intergenic spacers. Plant Biotechnol J. 12:344–353. 24283260 10.1111/pbi.12141

[evv130-B75] Xia X 2013 DAMBE5: a comprehensive software package for data analysis in molecular biology and evolution. Mol Biol Evol. 30:1720–1728. 23564938 10.1093/molbev/mst064 PMC3684854

[evv130-B76] Xu W Jameson D Tang B Higgs PG 2006 The relationship between the rate of molecular evolution and the rate of genome rearrangement in animal mitochondrial genomes. J Mol Evol. 63:375–392. 16838214 10.1007/s00239-005-0246-5

