# Supplementary material for: Dynamic Evolution of the Chloroplast Genome in the Green Algal Classes Pedinophyceae and Trebouxiophyceae
Source: Genome Biol Evol. 2015 Jul 1;7(7):2062–82. doi: 10.1093/gbe/evv130 (PMC4524492; doi:10.1093/gbe/evv130)
Supplement: Supplementary Data [file supp_7_7_2062__index.html]

Dynamic Evolution of the Chloroplast Genome in the Green Algal Classes Pedinophyceae and Trebouxiophyceae — Supplementary Data 

# Dynamic Evolution of the Chloroplast Genome in the Green Algal Classes Pedinophyceae and Trebouxiophyceae

## Supplementary Data

files

- Supplementary Data - pdf file
- Supplementary Data - pdf file
