# Supplementary material for: Dynamic Evolution of the Chloroplast Genome in the Green Algal Classes Pedinophyceae and Trebouxiophyceae
Source: Genome Biol Evol. 2015 Jul 1;7(7):2062–82. doi: 10.1093/gbe/evv130 (PMC4524492; doi:10.1093/gbe/evv130)
Supplement: Supplementary Data [file supp_evv130_Supplementary_figures_S2-S13.pdf]

Dynamic Evolution of the Chloroplast Genome in the Green Algal Classes Pedinophyceae and Trebouxiophyceae

Supplementary Figures S2-S13

Monique Turmel, Christian Otis, and Claude Lemieux

Institut de biologie intégrative et des systèmes, Département de biochimie, de microbiologie et de bio-informatique, Université Laval, Québec (QC) Canada

Corresponding Author: Monique Turmel
Institut de Biologie Intégrative et des Systèmes
1030 ave de la medicine, Pavillon Charles-Eugène Marchand
Université Laval, Québec (QC) Canada G1V 0A6;
phone: 418-656-2131 ext. 7623; Fax: 418-656-7176;
email: monique.turmel@bcm.ulaval.ca

Fig. S2. – Number, proportion and G+C content of repeats ≥ 30 bp in the chloroplast genomes examined in this study. Repeat elements are divided into six size categories that are denoted by different shades of gray. The average G+C content of the repeated sequences found in a given genome is provided along with the average G+C content of the unique sequences.

Fig. S3. – Structure of the *rpoB*, *rpoC2* and *tilS* genes in the chloroplast genomes examined in this study. A blue box indicates that the coding sequence consists of distinct ORFs that are not associated with typical group I or group II sequences, whereas a gray box indicates that the gene is not fragmented. A white box denotes the absence of the gene. All fragmented genes, with the exception of the *Pleurastrosarcina brevispinosa rpoC2 (rpoC2a, b, c* and *d)*, consist of two distinct ORFs. Note that the fragmented structure of *tilS* has not been reported for the previously described *Coccomyxa subellipsoidea* and *Trebouxiophyceae* sp. MX-AZ01 genomes (Servin-Garciduenas and Martinez-Romero 2012; Smith, et al. 2011).

Fig. S4. – Alignment of the pedinophycean *ssrA* genes with their prasinophycean and streptophyte orthologs. The sequences of the tRNA-like domains and mRNA-like coding region of the gene product (tmRNA) are framed. Genome coordinates are shown on each side of the alignment.

Fig. S5. – G+C content of protein-coding genes at each codon position among 63 green algal chloroplast genomes. G+C values were calculated using the concatenated nucleotide data set (79 genes, 15,468 codons) analyzed by Lemieux et al. (2014a). The shaded area denotes the six core trebouxiophyceans displaying a G+C-biased nucleotide composition.

Fig. S6. – Alignment of hypothetical proteins from three members of the *Prasiola* clade and the deep-sea γ-proteobacterium *Marinobacter manganoxydans.* The genomic coordinates of the ORF sequences corresponding to these proteins are as follows: "*Chlorella*" *mirabilis orf170a* (160463-160975) and *orf170b* (128638-128126); *Neocystis brevis orf169* (185786-186295), *orf171* (19096-19611), *orf179* (183005-183544) and *orf187* (1218-1781); and *Pabia signiensis orf205* (144859-144242).

Fig. S7. – Propensity of adjacent genes to be located on the same strand in pedinophycean and trebouxiophycean chloroplast genomes as evaluated by the sidedness index (Cui, et al. 2006).

Fig. S8. – Extent of chloroplast genome rearrangements in the Pedinophyceae (*A*) and five clades of the Trebouxiophyceae (*B-F*). For each clade, the genome alignment carried out using the ProgressiveMauve algorithm of Mauve 2.3.1 (Darling, et al. 2010) is presented. Below the alignment, a matrix shows the numbers of reversals separating all genome pairs; this matrix was produced by MGR (Bourque and Pevzner 2002) from the permutation matrix file generated by Mauve, which records the order and orientation of each locally collinear block.

Fig. S9. – Scatter plot comparing the relative lengths of the branches and internodes in the genome rearrangement and protein trees shown in fig. 5. The red dots represent the branches and internodes of core trebouxiophycean lineages that diverged after the *Oocystis*/*Geminella* clade, whereas the light blue dots represent earlier-diverging lineages. The branches and internodes in

the upper triangle defined by the dotted line are longer in the genome rearrangement tree relative to the protein tree.

Fig. S10. – Phylogenetic positions of pedinophycean and trebouxiophycean taxa as inferred from chloroplast gene order data using the tree reconstruction option of MLGO (Hu, et al. 2014). The gene order matrix contained all standard genes found in each genome, including all copies of duplicated genes. The 50% majority rule consensus tree of 1000 bootstrap replicates is shown.

Fig. S11. – Organization of the five genes making up the ancestral rDNA operon in the chloroplast genomes examined in this study. Two genes were considered to be linked when no standard genes were identified in the sequence separating them. Linked genes are connected by a horizontal line; coding regions shown above or below this line are transcribed to the right or to the left, respectively. Gene sequences and intergenic spacers are not drawn to scale, and introns are not represented (consult fig. 7 for intron content).

Fig. S12. – Scatter plot comparing the cell sizes and chloroplast genome sizes of pedinophycean and trebouxiophycean taxa. The five smallest genomes belong to the following pico- or nano-planctonic green algae: *Choricystis minor, Marsupiomonas* sp. NIES 1824, *Marvania geminata*, *Pedinomonas minor* and *Pseudochloris wilhelmii.* The trendline is a linear curve fit of the data.

Fig. S13. – Gene partitioning patterns of the IR-less chloroplast genomes examined in this study. The five genes composing the rDNA operon are highlighted in yellow. The color assigned

to each of the remaining genes is dependent upon the position of the corresponding gene relative to the rDNA operon in previously reported IR-containing prasinophycean and streptophyte cpDNAs displaying an ancestral gene partitioning pattern. The genes highlighted in dark blue are found within or near the SSC region in ancestral genomes (downstream of the rDNA operon), whereas those highlighted in orange are found within or near the LSC region (upstream of the rDNA operon). The genes shown in light blue were relocated from the LSC to the IR/SSC region in IR-containing pedinophycean and trebouxiophycean genomes (see fig. 4).

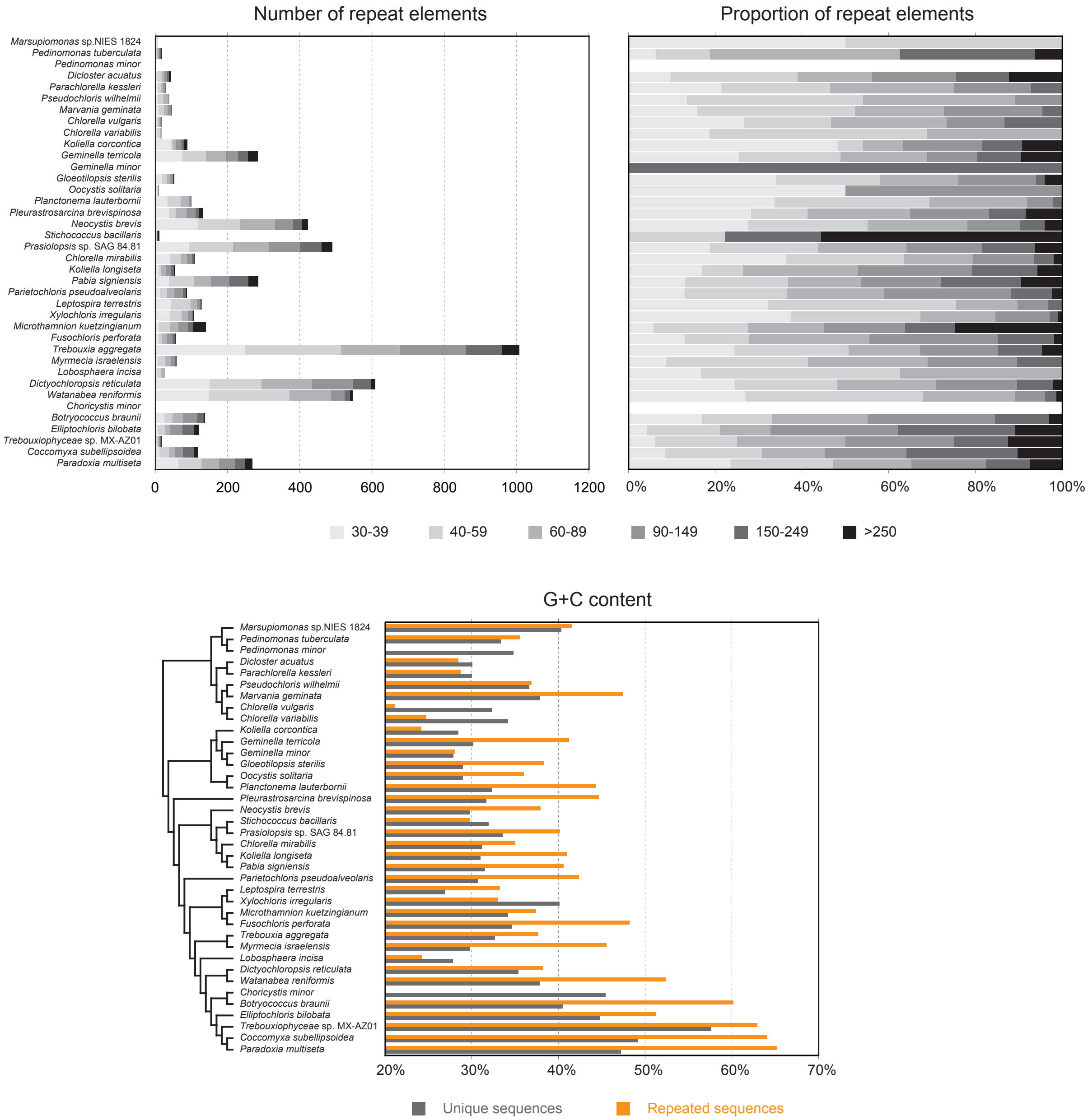
Number of repeat elements
Proportion of repeat elements
Marsupiomonas sp.NIES 1824
Pedinomonas tuberculata
Pedinomonas minor
Dicloster acuatus
Parachlorella kessleri
Pseudochloris wilhelmii
Marvania geminata
Chlorella vulgaris
Chlorella variabilis
Koliella corcontica
Geminella terricola
Geminella minor
Gloeotilopsis sterilis
Oocystis solitaria
Planctonema lauterbornii
Pleurastrosarcina brevispinosa
Neocystis brevis
Stichococcus bacillaris
Prasiolopsis sp. SAG 84.81
Chlorella mirabilis
Koliella longiseta
Pabia signiensis
Parietochloris pseudoalveolaris
Leptospira terrestris
Xylochloris irregularis
Microthamnion kuetzingianum
Fusochloris perforata
Trebouxia aggregata
Myrmecia israelensis
Lobosphaera incisa
Dictyochloropsis reticulata
Watanabea reniformis
Choricystis minor
Botryococcus braunii
Elliptochloris bilobata
Trebouxiophyceae sp. MX-AZ01
Coccomyxa subellipsoidea
Paradoxia multiseta
0
200
400
600
800
1000
1200
0%
20%
40%
60%
80%
100%
30-39
40-59
60-89
90-149
150-249
>250
G+C content
20%
30%
40%
50%
60%
70%
Unique sequences
Repeated sequences

Figure S2

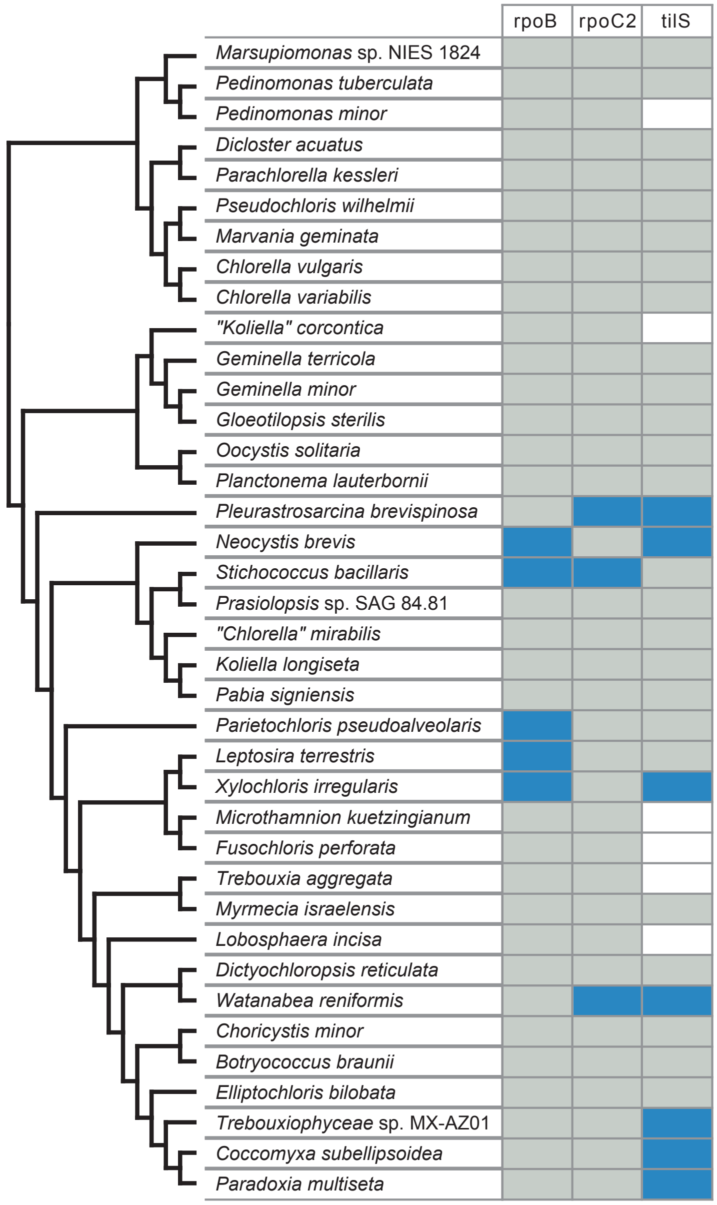
rpoB
rpoC2
tilS
Marsupiomonas sp. NIES 1824
Pedinomonas tuberculata
Pedinomonas minor
Dicloster acuatus
Parachlorella kessleri
Pseudochloris wilhelmii
Marvania geminata
Chlorella vulgaris
Chlorella variabilis
"Koliella" corcontica
Geminella terricola
Geminella minor
Gloeotilopsis sterilis
Oocystis solitaria
Planctonema lauterbornii
Pleurastrosarcina brevispinosa
Neocystis brevis
Stichococcus bacillaris
Prasiolopsis sp. SAG 84.81
"Chlorella" mirabilis
Koliella longiseta
Pabia signiensis
Parietochloris pseudoalveolaris
Leptosira terrestris
Xylochloris irregularis
Microthamnion kuetzingianum
Fusochloris perforata
Trebouxia aggregata
Myrmecia israelensis
Lobosphaera incisa
Dictyochloropsis reticulata
Watanabea reniformis
Choricystis minor
Botryococcus braunii
Elliptochloris bilobata
Trebouxiophyceae sp. MX-AZ01
Coccomyxa subellipsoidea
Paradoxia multiseta

Figure S3

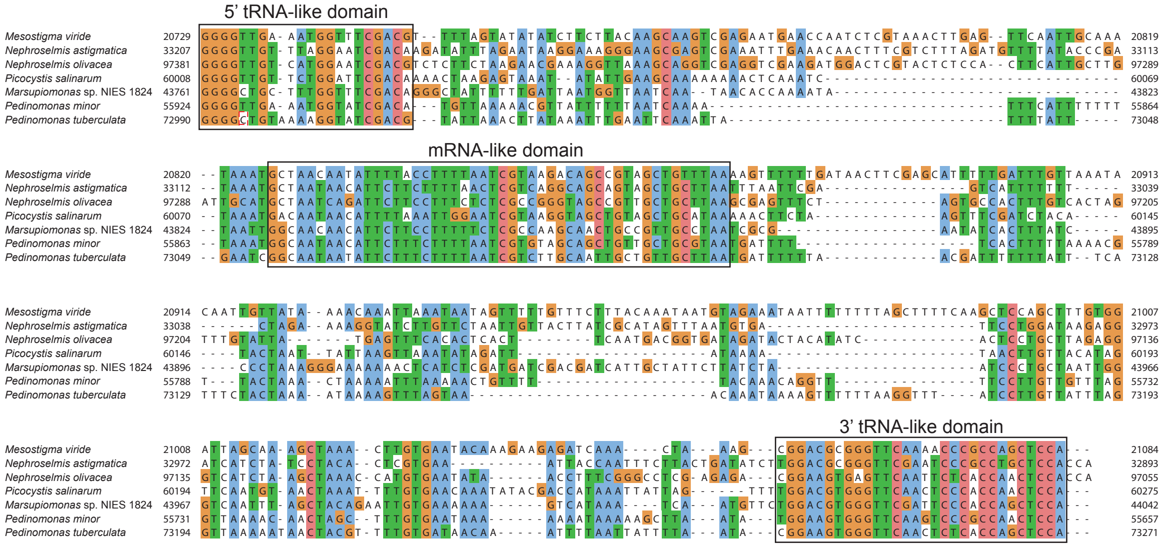
5' tRNA-like domain
mRNA-like domain
3' tRNA-like domain
Mesostigma viride
Nephroselmis astigmatica
Nephroselmis olivacea
Picocystis salinarum
Marsupiomonas sp. NIES 1824
Pedinomonas minor
Pedinomonas tuberculata

Figure S4

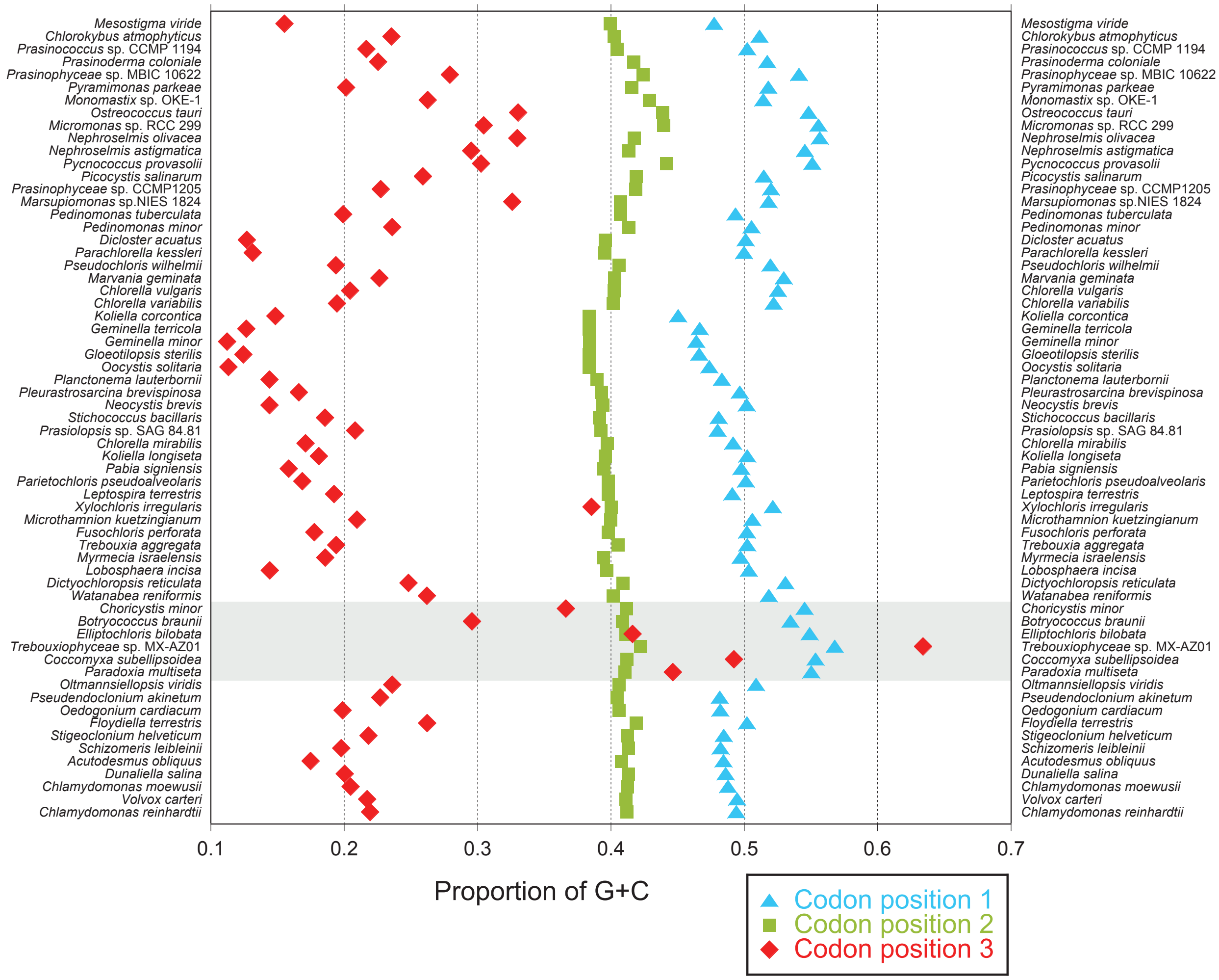
Mesostigma viride
Chlorokybus atmophyticus
Prasinococcus sp. CCMP 1194
Prasinoderma coloniale
Prasinophyceae sp. MBIC 10622
Pyramimonas parkeae
Monomastix sp. OKE-1
Ostreococcus tauri
Micromonas sp. RCC 299
Nephroselmis olivacea
Nephroselmis astigmatica
Pycnococcus provasolii
Picocystis salinarum
Prasinophyceae sp. CCMP1205
Marsupiomonas sp.NIES 1824
Pedinomonas tuberculata
Pedinomonas minor
Dicloster acuatus
Parachlorella kessleri
Pseudochloris wilhelmii
Marvania geminata
Chlorella vulgaris
Chlorella variabilis
Koliella corcontica
Geminella terricola
Geminella minor
Gloeotilopsis sterilis
Oocystis solitaria
Planctonema lauterbornii
Pleurastrosarcina brevispinosa
Neocystis brevis
Stichococcus bacillaris
Prasiolopsis sp. SAG 84.81
Chlorella mirabilis
Koliella longiseta
Pabia signiensis
Parietochloris pseudoalveolaris
Leptospira terrestris
Xylochloris irregularis
Microthamnion kuetzingianum
Fusochloris perforata
Trebouxia aggregata
Myrmecia israelensis
Lobosphaera incisa
Dictyochloropsis reticulata
Watanabea reniformis
Choricystis minor
Botryococcus braunii
Elliptochloris bilobata
Trebouxiophyceae sp. MX-AZ01
Coccomyxa subellipsoidea
Paradoxia multiseta
Oltmannsiellopsis viridis
Pseudendoclonium akinetum
Oedogonium cardiacum
Floydiella terrestris
Stigeoclonium helveticum
Schizomeris leibleinii
Acutodesmus obliquus
Dunaliella salina
Chlamydomonas moewusii
Volvox carteri
Chlamydomonas reinhardtii
0.1
0.2
0.3
0.4
0.5
0.6
0.7
Proportion of G+C
Codon position 1
Codon position 2
Codon position 3

Figure S5

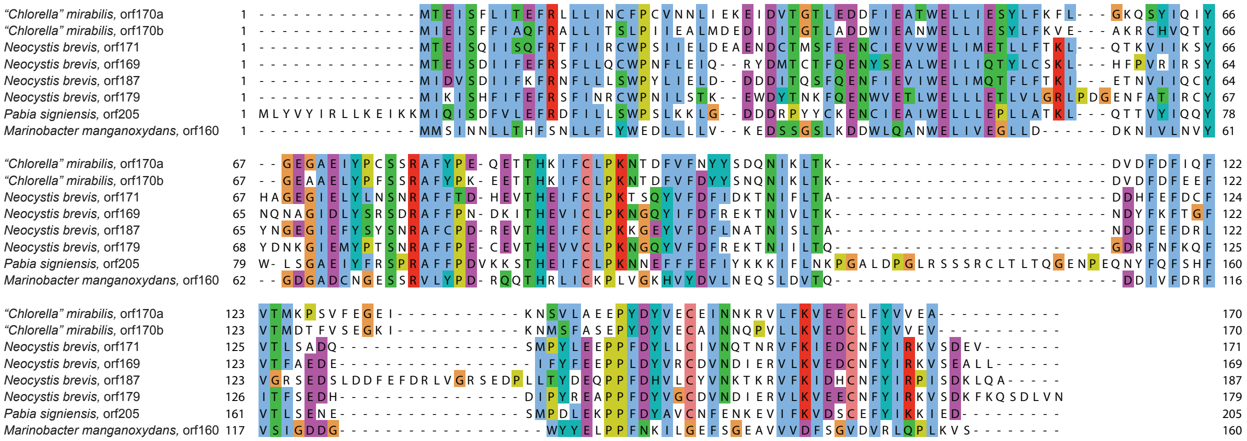

Figure S6

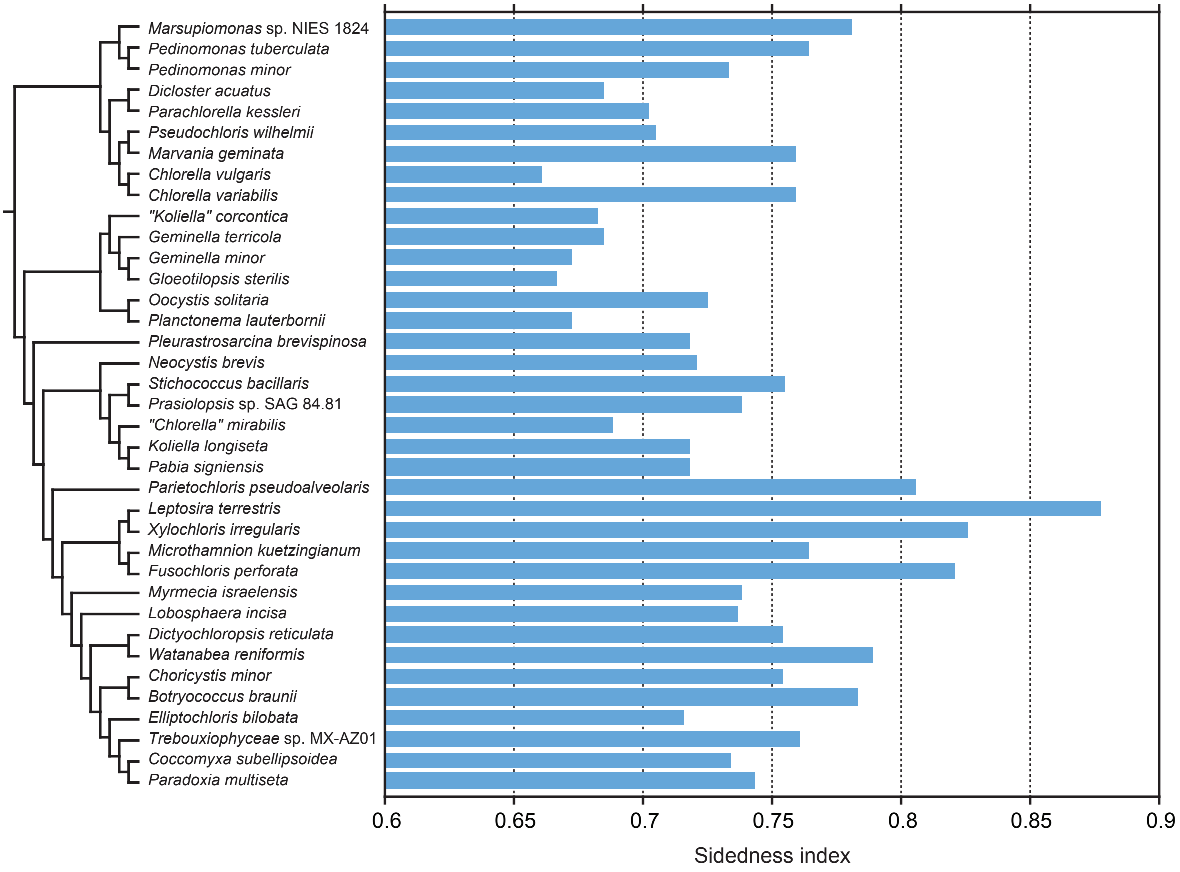
Marsupiomonas sp. NIES 1824
Pedinomonas tuberculata
Pedinomonas minor
Dicloster acuatus
Parachlorella kessleri
Pseudochloris wilhelmii
Marvania geminata
Chlorella vulgaris
Chlorella variabilis
"Koliella" corcontica
Geminella terricola
Geminella minor
Gloeotilopsis sterilis
Oocystis solitaria
Planctonema lauterbornii
Pleurastrosarcina brevispinosa
Neocystis brevis
Stichococcus bacillaris
Prasiolopsis sp. SAG 84.81
"Chlorella" mirabilis
Koliella longiseta
Pabia signiensis
Parietochloris pseudoalveolaris
Leptosira terrestris
Xylochloris irregularis
Microthamnion kuetzingianum
Fusochloris perforata
Myrmecia israelensis
Lobosphaera incisa
Dictyochloropsis reticulata
Watanabea reniformis
Choricystis minor
Botryococcus braunii
Elliptochloris bilobata
Trebouxiophyceae sp. MX-AZ01
Coccomyxa subellipsoidea
Paradoxia multiseta
0.6
0.65
0.7
0.75
0.8
0.85
0.9
Sidedness index

Figure S7

Pedinophyceae

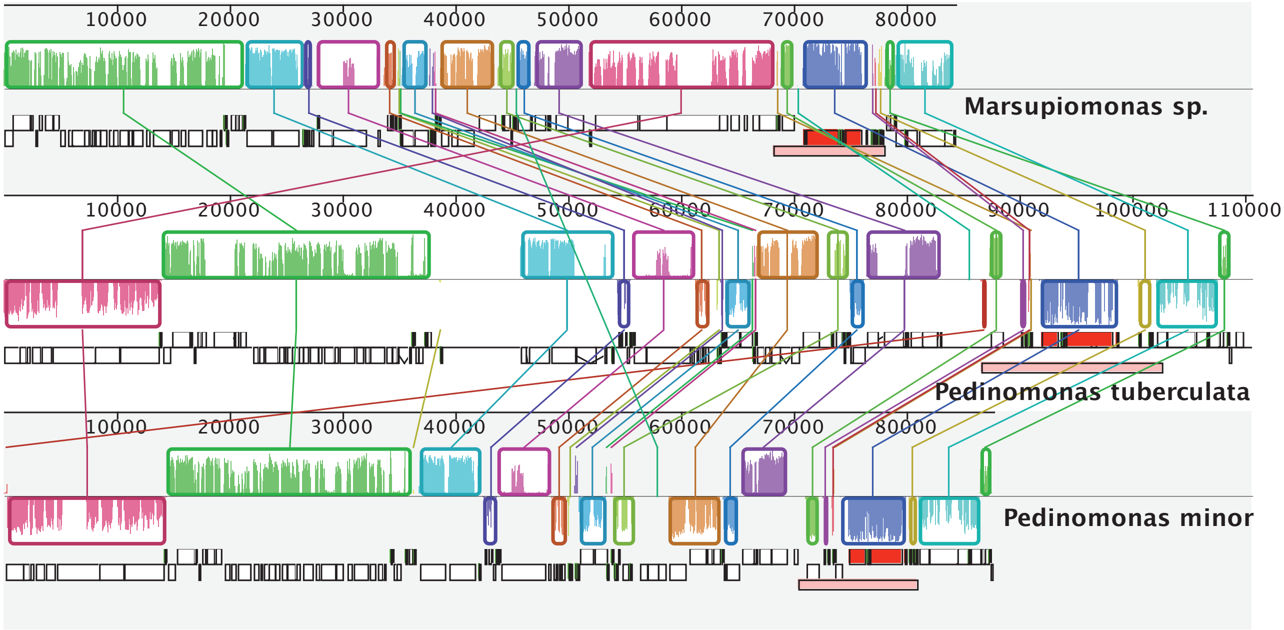

	MARSspeci	PEDItuber	PEDIminor
MARSspeci	0	14	17
PEDItuber	14	0	3
PEDIminor	17	3	0

Figure S8A

Chlorellales

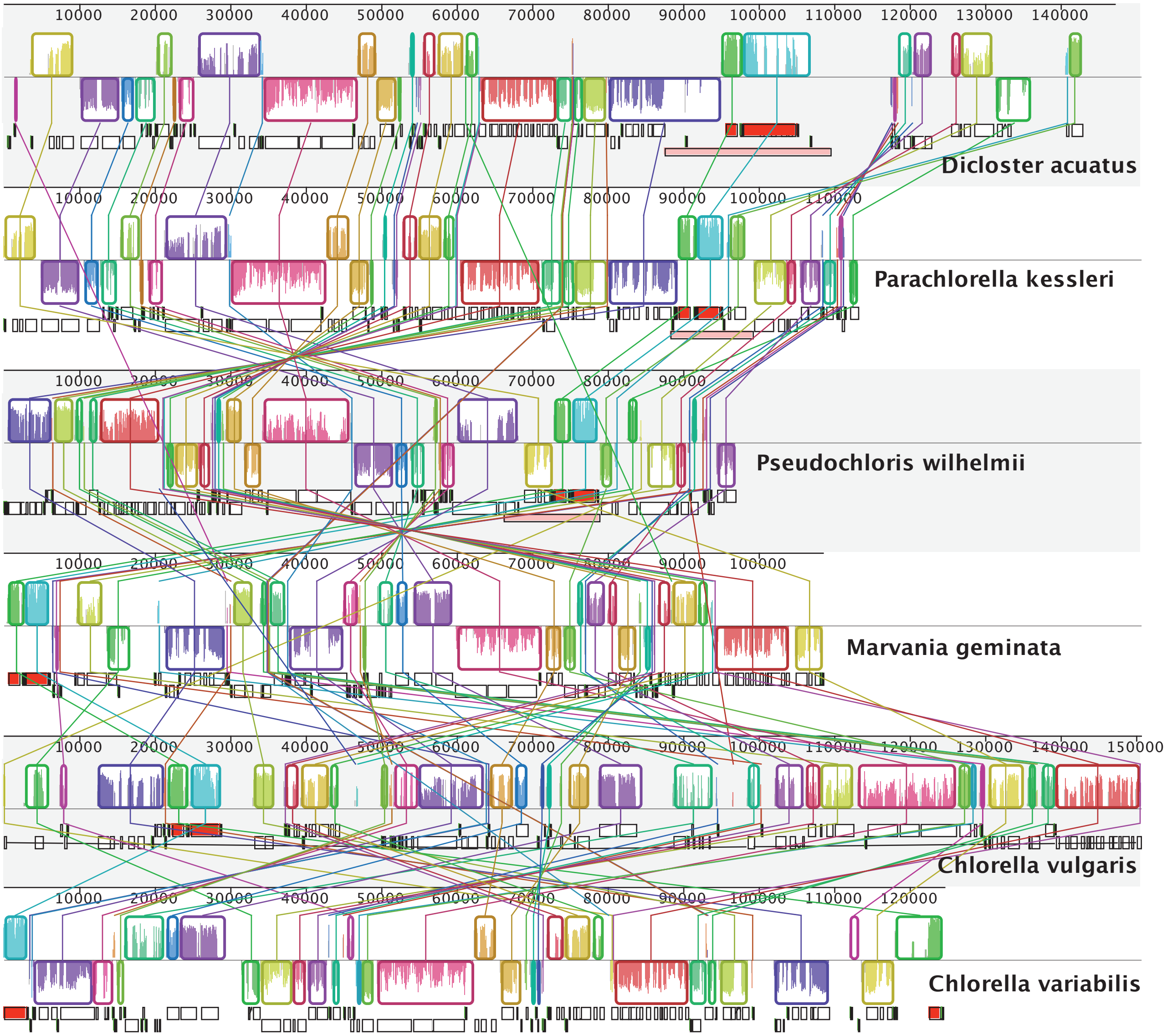

	CHLOvulga	CHLOvaria	MARVgemin	PSEUwilhe	PARAkessl	DICLacuat
CHLOvulga	0	19	27	24	24	24
CHLOvaria	19	0	13	12	9	8
MARVgemin	27	13	0	14	14	11
PSEUwilhe	24	12	14	0	10	8
PARAkessl	24	9	14	10	0	3
DICLacuat	24	8	11	8	3	0

Figure S8B

Geminella/Oocystis clade

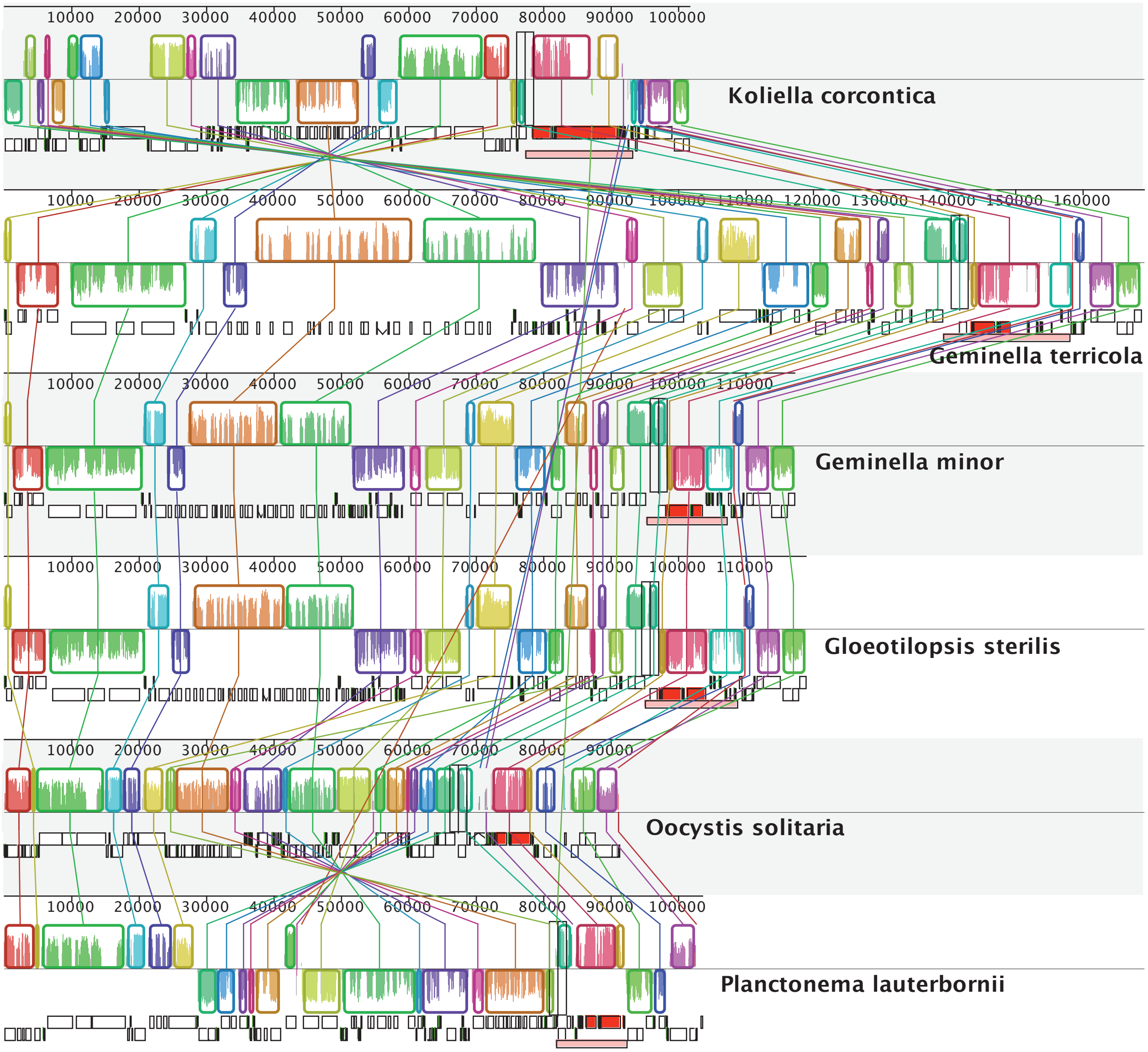

	OOCYsolit	PLANlaute	GLOEsteri	GEMIminor	GEMIterri	KOLIcorco
OOCYsolit	0	3	17	17	18	17
PLANlaute	3	0	18	18	19	18
GLOEsteri	17	18	0	0	1	4
GEMIminor	17	18	0	0	1	4
GEMIterri	18	19	1	1	0	5
KOLIcorco	17	18	4	4	5	0

Figure S8C

Prasiola clade

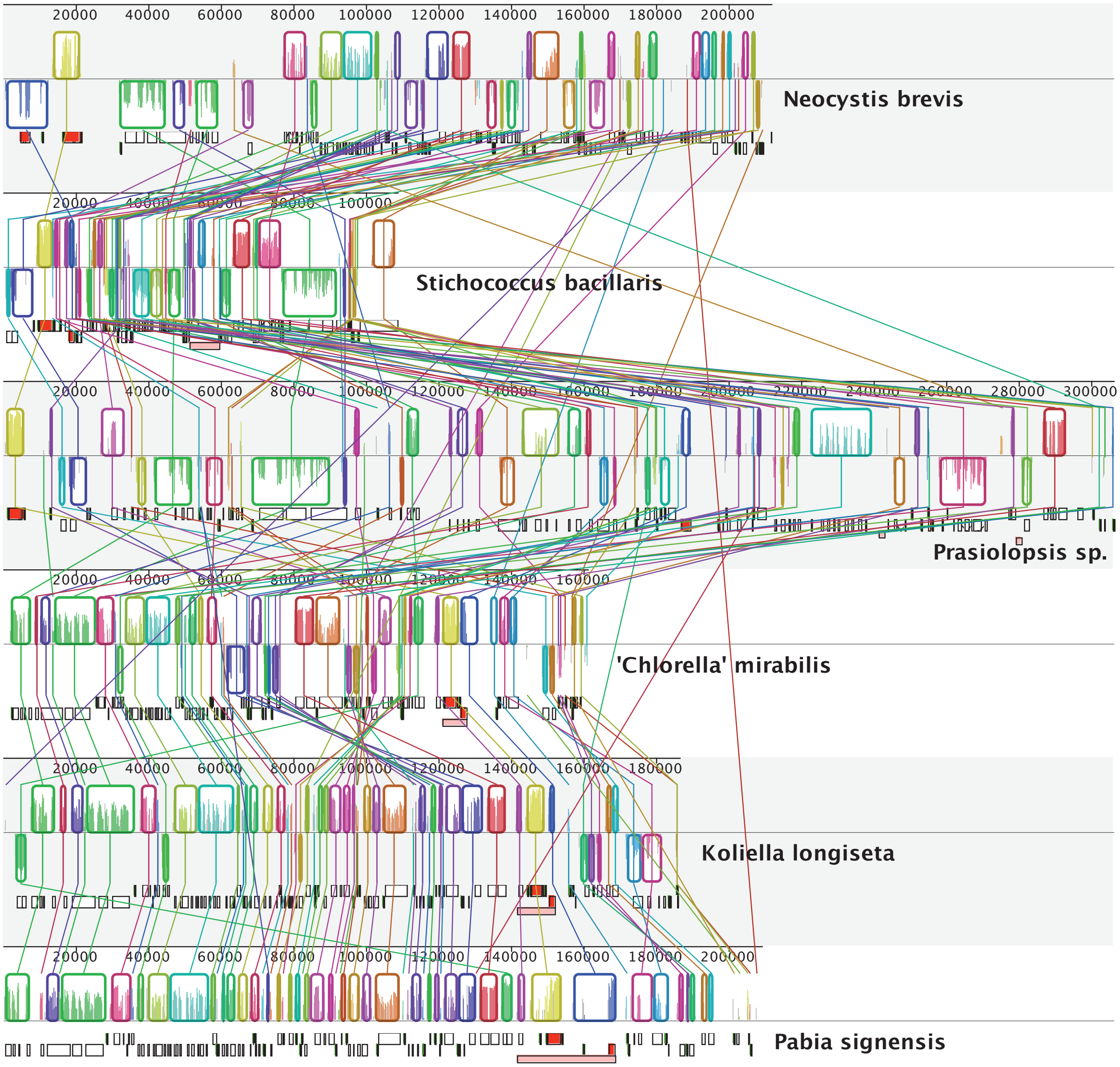

	PABIsigni	KOLIlongi	CHLOmirab	PRASspeci	STICbacil	NEOCbrevi
PABIsigni	0	8	14	35	38	20
KOLIlongi	8	0	14	33	39	17
CHLOmirab	14	14	0	35	36	18
PRASspeci	35	33	35	0	36	33
STICbacil	38	39	36	36	0	36
NEOCbrevi	20	17	18	33	36	0

Figure S8D

Xylochloris/Microthamniales clade

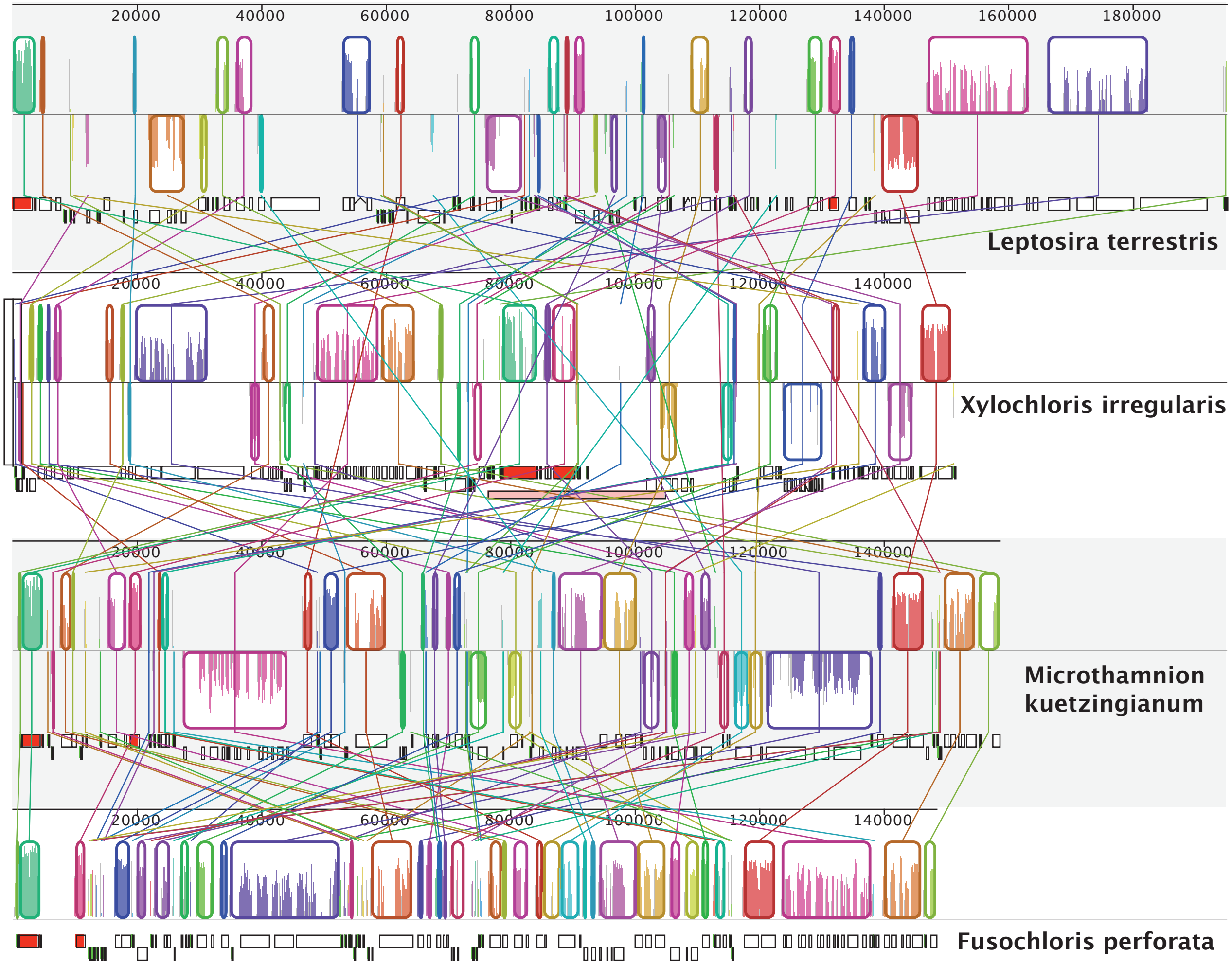

	FUSOperfo	MICRkuetz	XYLOirreg	LEPTterre
FUSOperfo	0	29	37	35
MICRkuetz	29	0	35	34
XYLOirreg	37	35	0	33
LEPTterre	35	34	33	0

Figure S8E

Trebouxiales/Lobosphaera/Watanabea/Choricystis/Elliptochloris clade

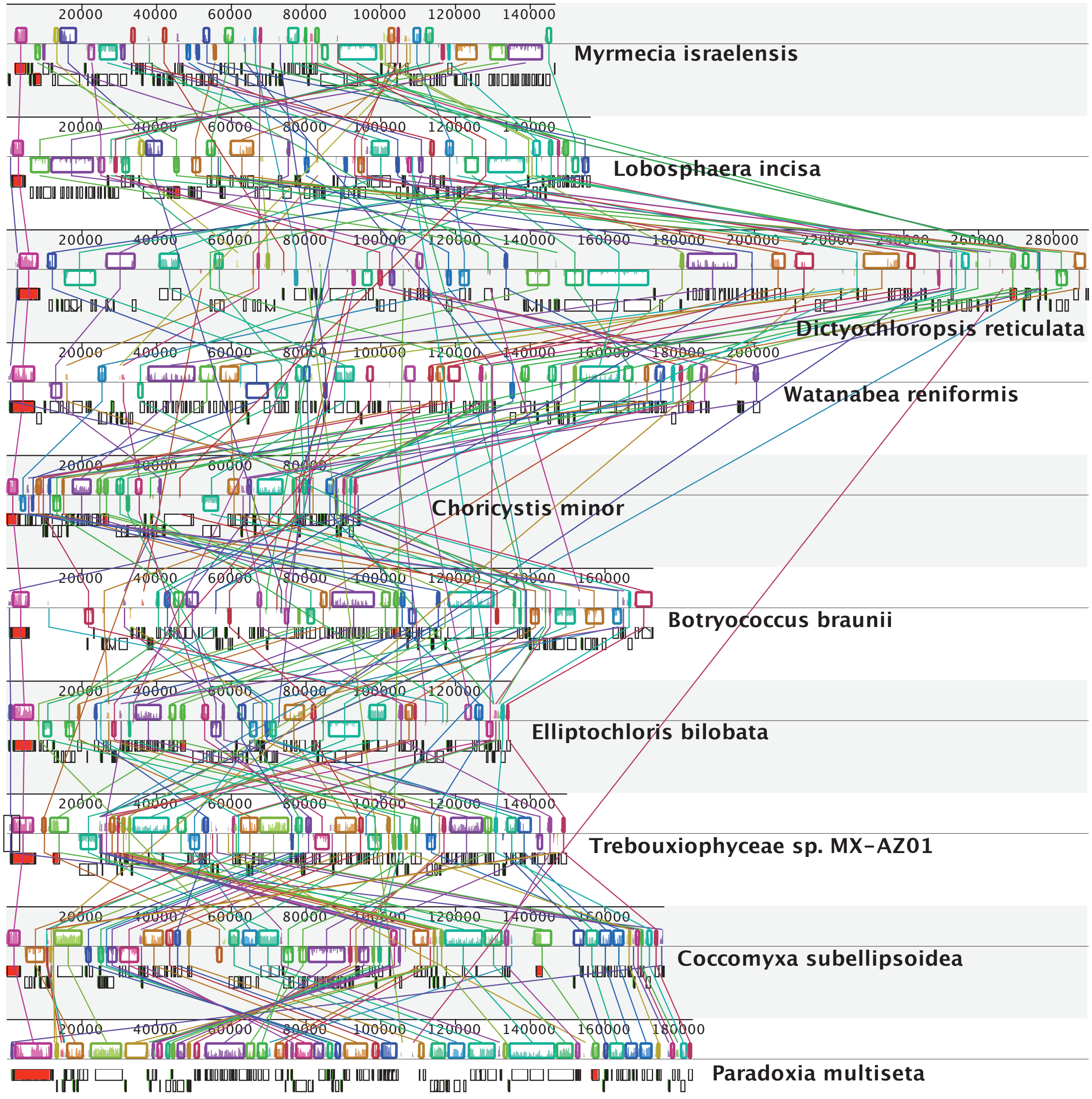

	PARAmulti	COCCsubel	TREBspAZ0	ELLIbilob	BOTRbraun	CHORminor	WATArenif	DICTretic	LOBOincis	MYRMisrae
PARAmulti	0	5	19	17	20	18	22	22	22	22
COCCsubel	5	0	16	16	20	18	22	22	22	21
TREBspAZ0	19	16	0	20	22	19	22	22	19	22
ELLIbilob	17	16	20	0	21	17	18	23	20	18
BOTRbraun	20	20	22	21	0	19	22	25	24	21
CHORminor	18	18	19	17	19	0	21	24	18	19
WATArenif	22	22	22	18	22	21	0	25	21	19
DICTretic	22	22	22	23	25	24	25	0	23	23
LOBOincis	22	22	19	20	24	18	21	23	0	19
MYRMisrae	22	21	22	18	21	19	19	23	19	0

Figure S8F

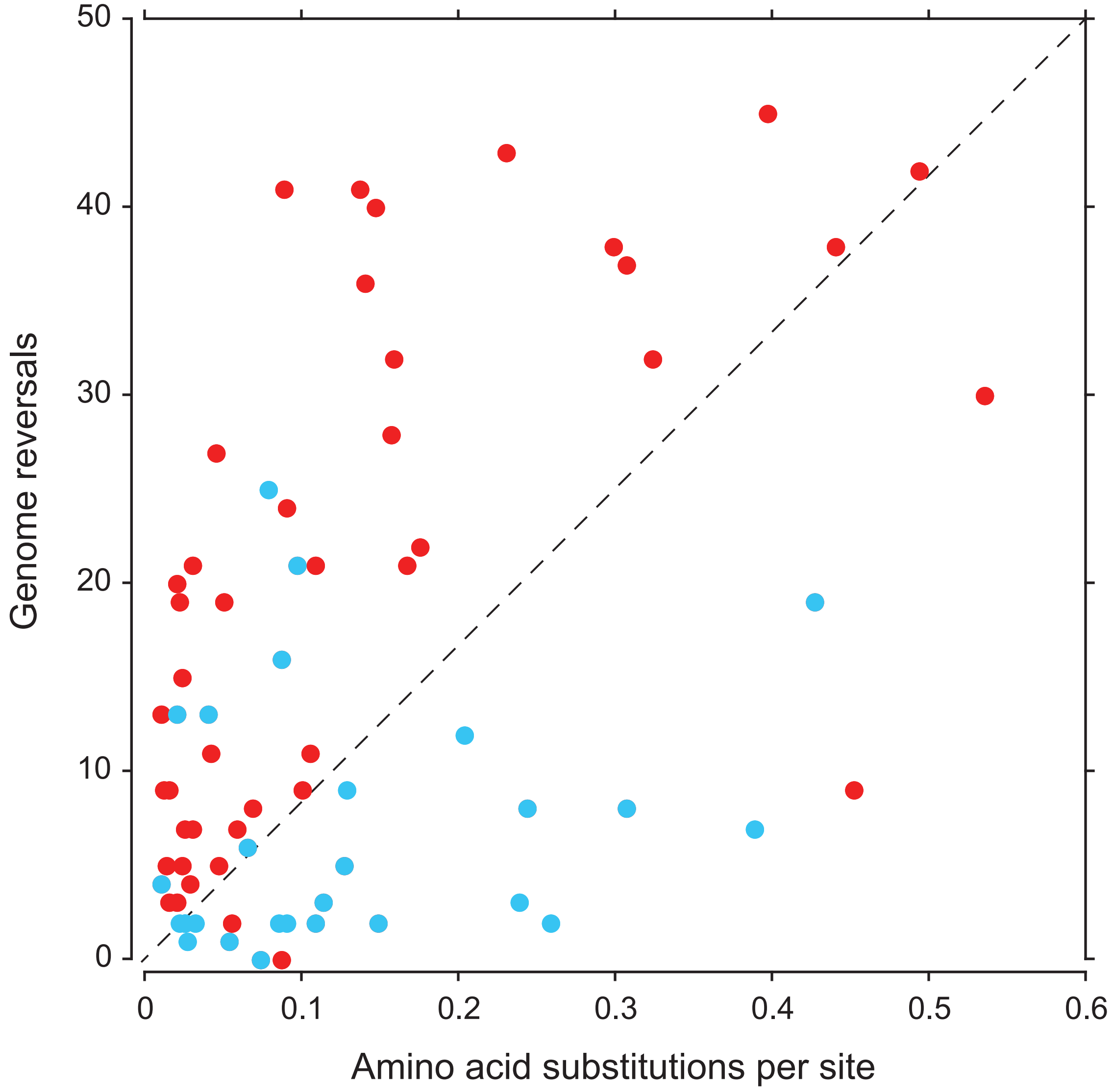

50
40
30
20
10
0
Genome reversals
0
0.1
0.2
0.3
0.4
0.5
0.6
Amino acid substitutions per site

Figure S9

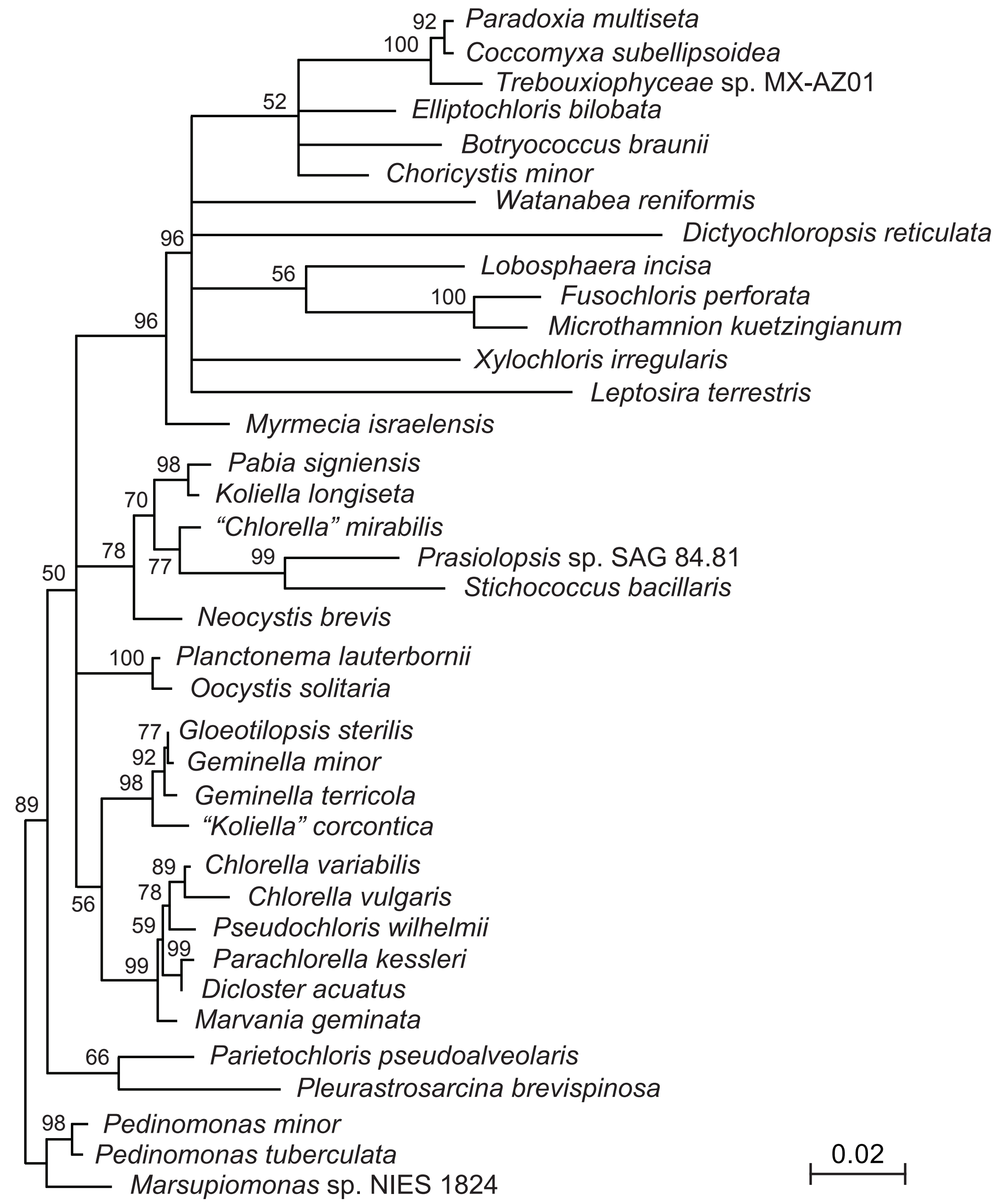

Paradoxia multiseta
Coccomyxa subellipsoidea
Trebouxiophyceae sp. MX-AZ01
Elliptochloris bilobata
Botryococcus braunii
Choricystis minor
Watanabea reniformis
Dictyochloropsis reticulata
Lobosphaera incisa
Fusochloris perforata
Microthamnion kuetzingianum
Xylochloris irregularis
Leptosira terrestris
Myrmecia israelensis
Pabia signiensis
Koliella longiseta
"Chlorella" mirabilis
Prasiolopsis sp. SAG 84.81
Stichococcus bacillaris
Neocystis brevis
Planctonema lauterbornii
Oocystis solitaria
Gloeotilopsis sterilis
Geminella minor
Geminella terricola
"Koliella" corcontica
Chlorella variabilis
Chlorella vulgaris
Pseudochloris wilhelmii
Parachlorella kessleri
Dicloster acuatus
Marvania geminata
Parietochloris pseudoalveolaris
Pleurastrosarcina brevispinosa
Pedinomonas minor
Pedinomonas tuberculata
Marsupiomonas sp. NIES 1824
0.02
92
100
52
96
96
56
100
98
70
78
77
99
50
100
77
92
98
89
89
78
59
99
99
56
66
98

Figure S10

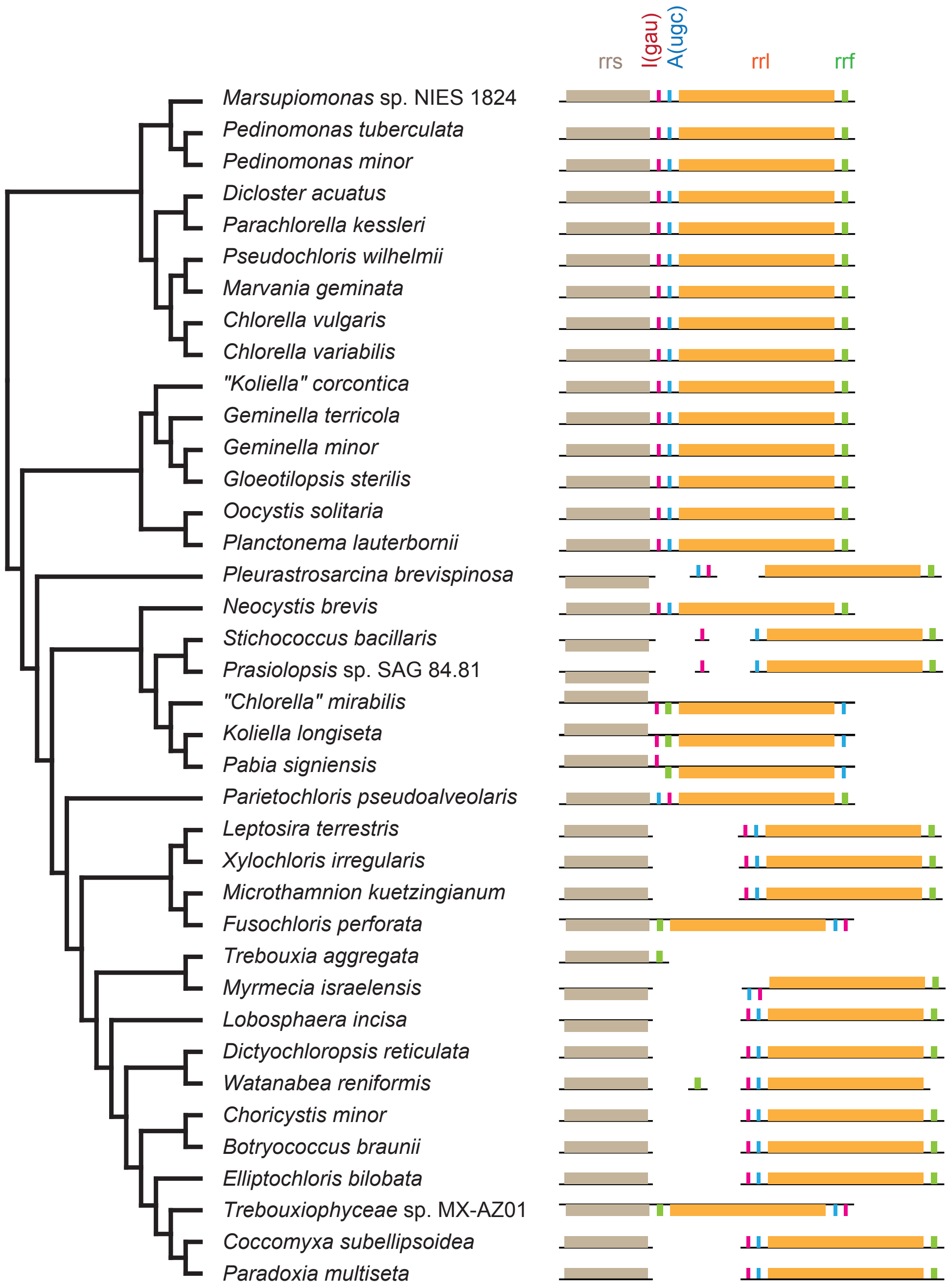
rrs
I(gau)
A(ugc)
rrl
rrf
Marsupiomonas sp. NIES 1824
Pedinomonas tuberculata
Pedinomonas minor
Dicloster acuatus
Parachlorella kessleri
Pseudochloris wilhelmii
Marvania geminata
Chlorella vulgaris
Chlorella variabilis
"Koliella" corcontica
Geminella terricola
Geminella minor
Gloeotilopsis sterilis
Oocystis solitaria
Planctonema lauterbornii
Pleurastrosarcina brevispinosa
Neocystis brevis
Stichococcus bacillaris
Prasiolopsis sp. SAG 84.81
"Chlorella" mirabilis
Koliella longiseta
Pabia signiensis
Parietochloris pseudoalveolaris
Leptosira terrestris
Xylochloris irregularis
Microthamnion kuetzingianum
Fusochloris perforata
Trebouxia aggregata
Myrmecia israelensis
Lobosphaera incisa
Dictyochloropsis reticulata
Watanabea reniformis
Choricystis minor
Botryococcus braunii
Elliptochloris bilobata
Trebouxiophyceae sp. MX-AZ01
Coccomyxa subellipsoidea
Paradoxia multiseta

Figure S11

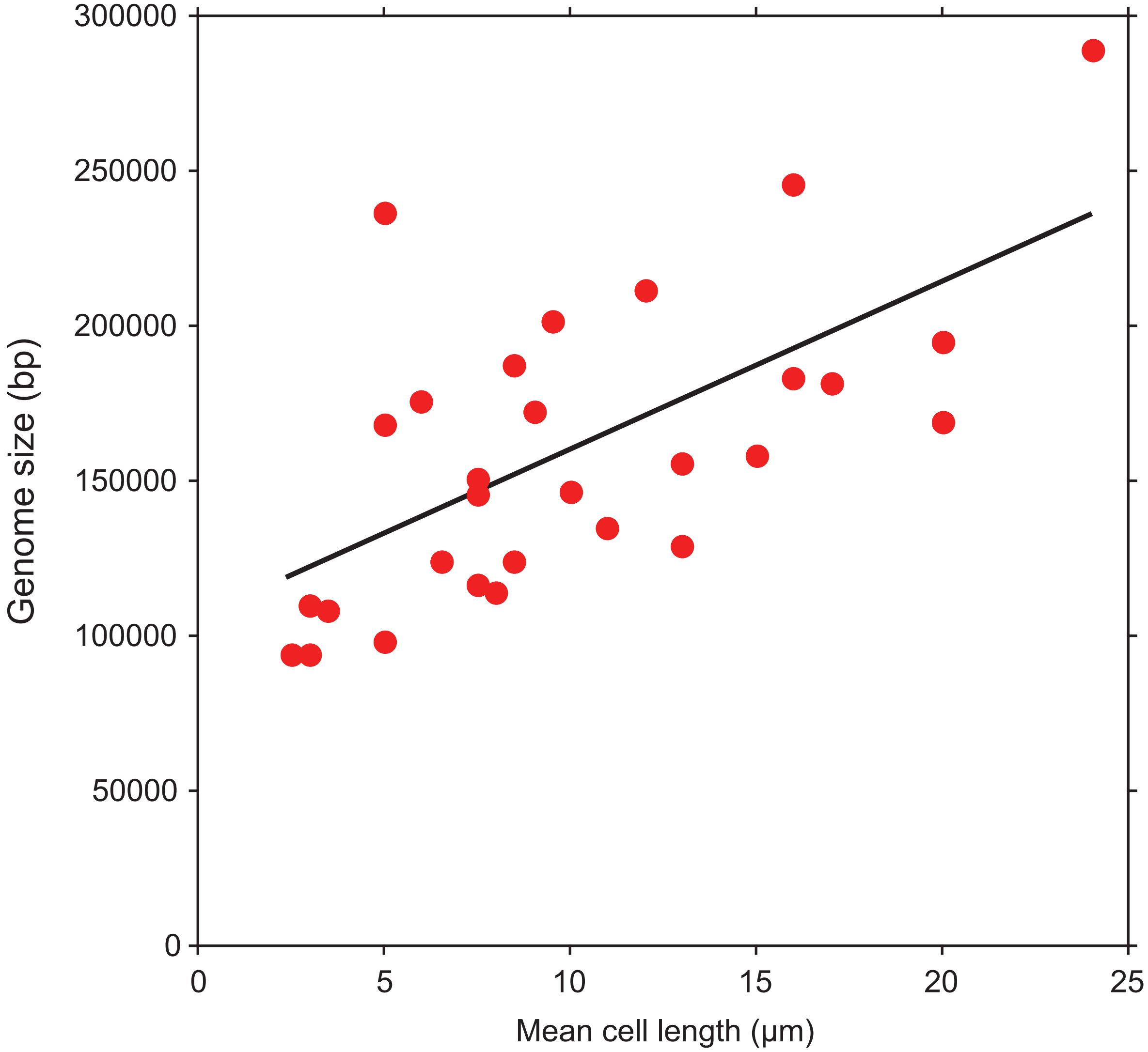

300000
250000
200000
150000
100000
50000
0
Genome size (bp)
0
5
10
15
20
25
Mean cell length (μm)

Figure S12

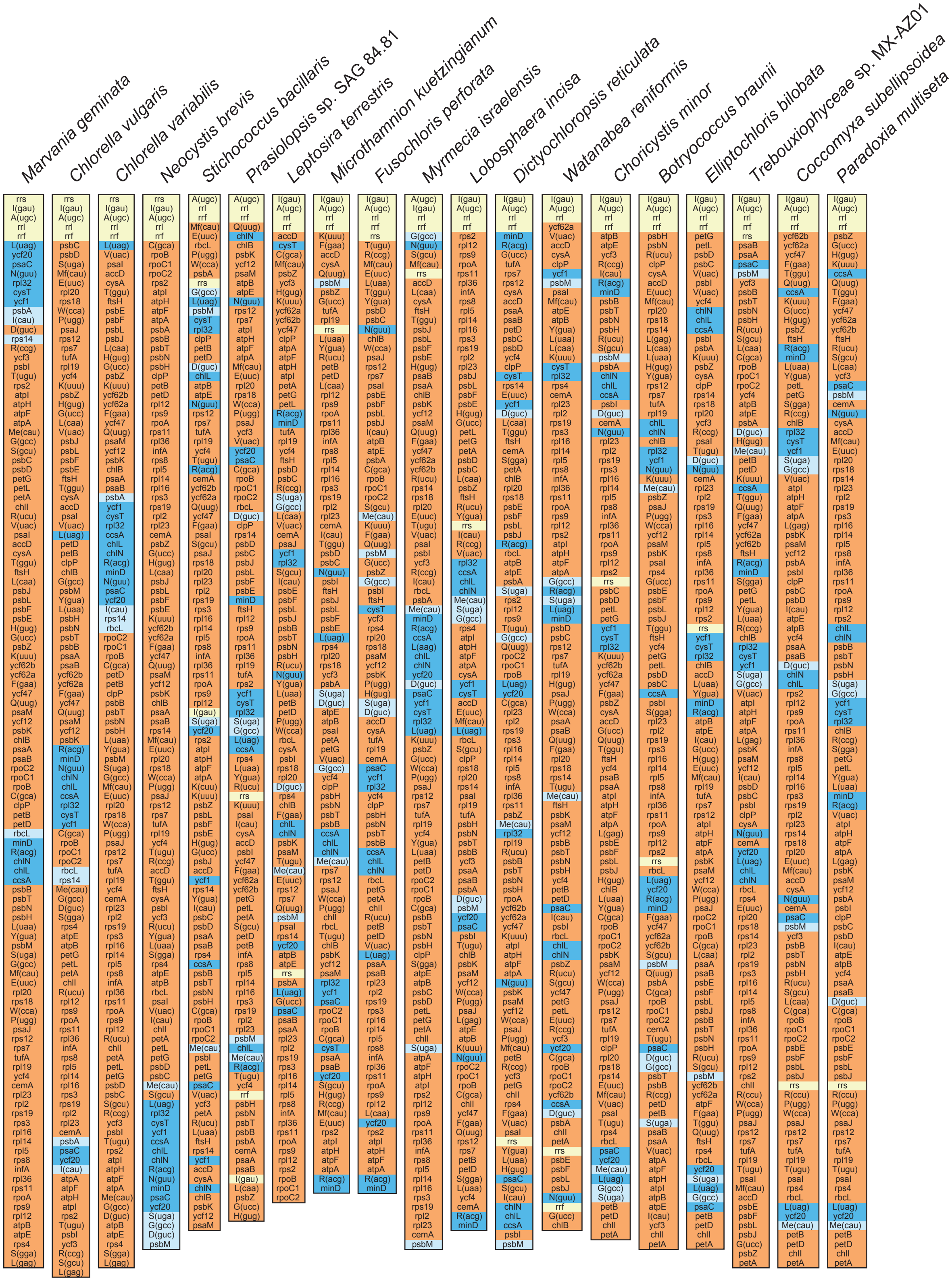
Marvania geminata
Chlorella vulgaris
Chlorella variabilis
Neocystis brevis
Stichococcus bacillaris
Prasiolopsis sp. SAG 84.81
Leptosira terrestris
Microthamnion kuetzingianum
Fusochloris perforata
Myrmecia israelensis
Lobosphaera incisa
Dictyochloropsis reticulata
Watanabea reniformis
Choricystis minor
Botryococcus braunii
Elliptochloris bilobata
Trebouxiophyceae sp. MX-AZ01
Coccomyxa subellipsoidea
Paradoxia multiseta

Figure S13
