# Supplementary material for: Dynamic Evolution of the Chloroplast Genome in the Green Algal Classes Pedinophyceae and Trebouxiophyceae
Source: Genome Biol Evol. 2015 Jul 1;7(7):2062–82. doi: 10.1093/gbe/evv130 (PMC4524492; doi:10.1093/gbe/evv130)
Supplement: Supplementary Data [file supp_evv130_Supplementary_figure_S1.pdf]

Dynamic Evolution of the Chloroplast Genome in the Green Algal Classes Pedinophyceae and Trebouxiophyceae

Supplementary Figure S1

Monique Turmel, Christian Otis, and Claude Lemieux

Institut de biologie intégrative et des systèmes, Département de biochimie, de microbiologie et de bio-informatique, Université Laval, Québec (QC) Canada

Corresponding Author: Monique Turmel
Institut de Biologie Intégrative et des Systèmes
1030 ave de la medicine, Pavillon Charles-Eugène Marchand
Université Laval, Québec (QC) Canada G1V 0A6;
phone: 418-656-2131 ext. 7623; Fax: 418-656-7176;
email: monique.turmel@bcm.ulaval.ca

Fig. S1. – Gene maps of all chloroplast genomes compared in this study, except that of *Trebouxia aggregata*. Lemieux, et al. (2014a) recently reported 29 of these genome sequences and used them to construct the multigene data sets they subjected to phylogenetic analyses. Filled boxes represent genes, with colors denoting gene categories as indicated in the legend at the bottom the figure. Genes on the outside of the map are transcribed counterclockwise; those on the inside are transcribed clockwise. The inner ring shows variations in G+C content and the positions of the IR and single-copy regions (SSC and LSC). The circle inside the G+C content graph marks the 50% threshold (dark gray, G+C; light gray, A+T). All indicated ORFs potentially code for proteins similar to previously reported proteins with known functions and/or recognized protein domains (Table 2) or hypothetical proteins (Fig. S5). Sites corresponding to sequence gaps in the partially sequenced cpDNAs of *Oocystis solitaria* and *Pleurastrosarcina brevispinosa* are indicated by thick lines. GenBank accession numbers of all genomes are indicated in Table 1.

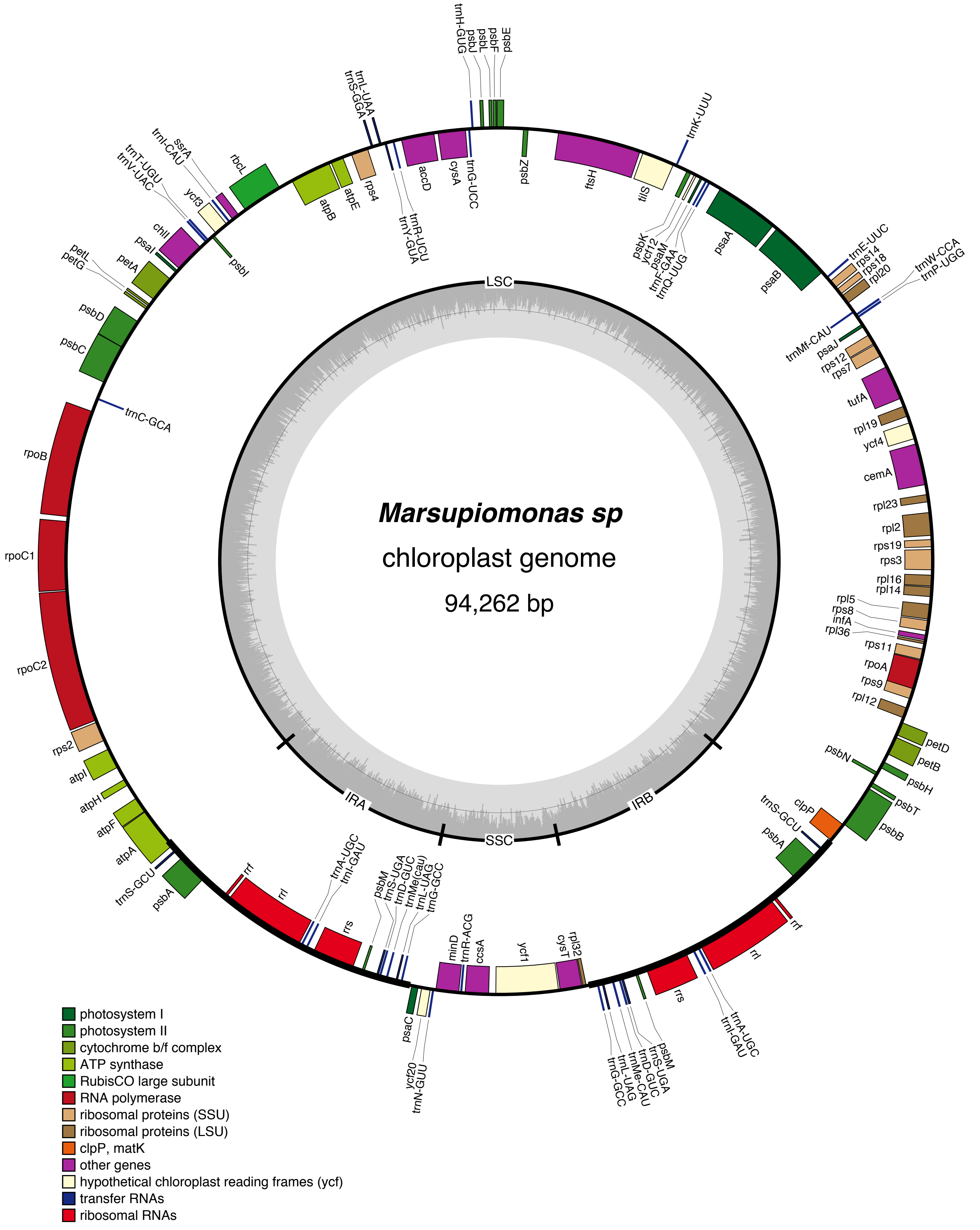

Marsupiomonas sp
chloroplast genome
94,262 bp
LSC
IRA
SSC
IRB
trnH-GUG
psbJ
psbL
psbF
psbE
psbZ
ftsH
tilS
trnK-UUU
psbK
ycf12
psaM
trnF-GAA
trnQ-UUG
psaA
psaB
trnE-UUC
rps14
rps18
rpl20
trnW-CCA
trnP-UGG
trnMf-CAU
psaJ
rps12
rps7
tufA
rpl19
ycf4
cemA
rpl23
rpl2
rps19
rps3
rpl16
rpl14
rpl5
rps8
infA
rpl36
rps11
rpoA
rps9
rpl12
petD
petB
psbN
psbH
psbT
clpP
trnS-GCU
psbB
psbA
rrf
rrl
trnA-UGC
trnI-GAU
rrs
psbM
trnS-UGA
trnD-GUC
trnMe-CAU
trnL-UAG
trnG-GCC
rpl32
cysT
ycf1
ccsA
trnR-ACG
minD
psaC
ycf20
trnN-GUU
trnMe(cau)
atpA
atpF
atpH
atpI
rps2
rpoC2
rpoC1
rpoB
trnC-GCA
psbC
psbD
petA
petL
petG
psaI
chlI
psbI
trnT-UGU
trnV-UAC
ycf3
trnI-CAU
ssrA
rbcL
atpB
atpE
rps4
trnS-GGA
trnL-UAA
trnR-UCU
trnY-GUA
accD
cysA
trnG-UCC
photosystem I
photosystem II
cytochrome b/f complex
ATP synthase
RubisCO large subunit
RNA polymerase
ribosomal proteins (SSU)
ribosomal proteins (LSU)
clpP, matK
other genes
hypothetical chloroplast reading frames (ycf)
transfer RNAs
ribosomal RNAs

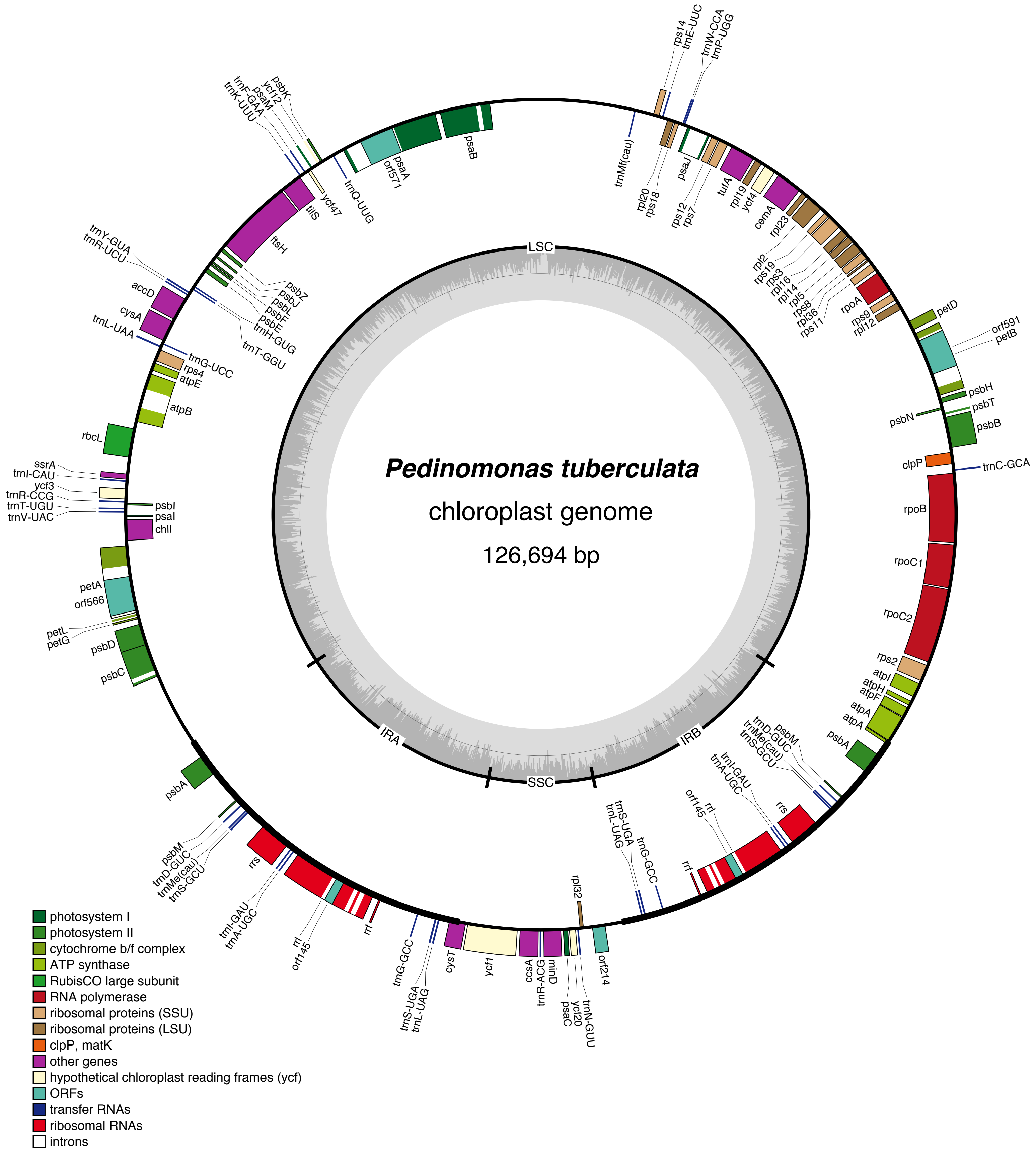
Pedinomonas tuberculata
chloroplast genome
126,694 bp
LSC
IRA
SSC
IRB
psaB
psaA
orf571
trnQ-UUG
ycf47
tilS
ftsH
psbK
ycf12
psaM
trnF-GAA
trnK-UUU
trnY-GUA
trnR-UCU
accD
cysA
trnL-UAA
psbZ
psbJ
psbL
psbF
psbE
trnH-GUG
trnT-GGU
trnG-UCC
rps4
atpE
atpB
rbcL
ssrA
trnI-CAU
ycf3
trnR-CCG
trnT-UGU
trnV-UAC
psbI
psaI
chlI
petA
orf566
petL
petG
psbD
psbC
psbA
psbM
trnD-GUC
trnMe(cau)
trnS-GCU
rrs
trnI-GAU
trnA-UGC
rrl
orf145
rrf
trnG-GCC
trnS-UGA
trnL-UAG
cysT
ycf1
ccsA
trnR-ACG
minD
psaC
ycf20
trnN-GUU
orf214
rpl32
trnMf(cau)
rps14
trnE-UUC
trnW-CCA
trnP-UGG
rpl20
rps18
psaJ
rps12
rps7
tufA
rpl19
ycf4
cemA
rpl23
rpl2
rps19
rps3
rpl16
rpl14
rpl5
rps8
rpl36
rps11
rpoA
rps9
rpl12
petD
orf591
petB
psbH
psbT
psbN
psbB
clpP
trnC-GCA
rpoB
rpoC1
rpoC2
rps2
atpI
atpH
atpF
atpA
photosystem I
photosystem II
cytochrome b/f complex
ATP synthase
RubisCO large subunit
RNA polymerase
ribosomal proteins (SSU)
ribosomal proteins (LSU)
clpP, matK
other genes
hypothetical chloroplast reading frames (ycf)
ORFs
transfer RNAs
ribosomal RNAs
introns

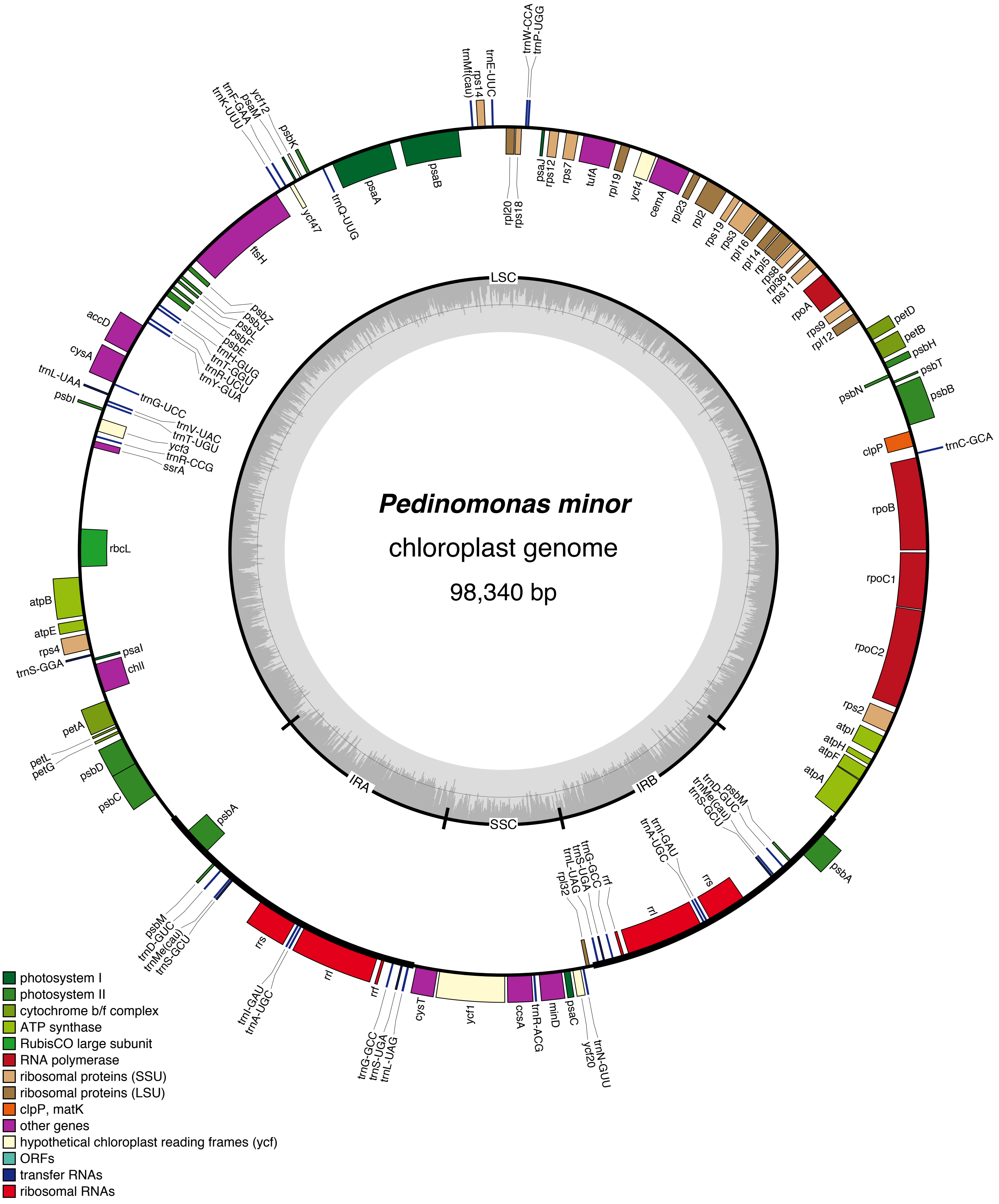
Pedinomonas minor
chloroplast genome
98,340 bp
LSC
IRA
SSC
IRB
psaA
psaB
ftsH
accD
cysA
rbcL
atpB
atpE
rps4
chlI
petA
psbD
psbC
psbA
rrs
rrl
rrf
cysT
ycf1
ccsA
minD
psaC
rpoA
rpoB
rpoC1
rpoC2
rps2
atpI
atpH
atpF
atpA
clpP
psbB
tufA
cemA
ycf4
rpl2
rpl23
rps19
rps3
rpl16
rpl14
rpl5
rps8
rpl36
rps11
rps9
rpl12
petD
petB
psbH
psbT
psbN
trnC-GCA
photosystem I
photosystem II
cytochrome b/f complex
ATP synthase
RubisCO large subunit
RNA polymerase
ribosomal proteins (SSU)
ribosomal proteins (LSU)
clpP, matK
other genes
hypothetical chloroplast reading frames (ycf)
ORFs
transfer RNAs
ribosomal RNAs

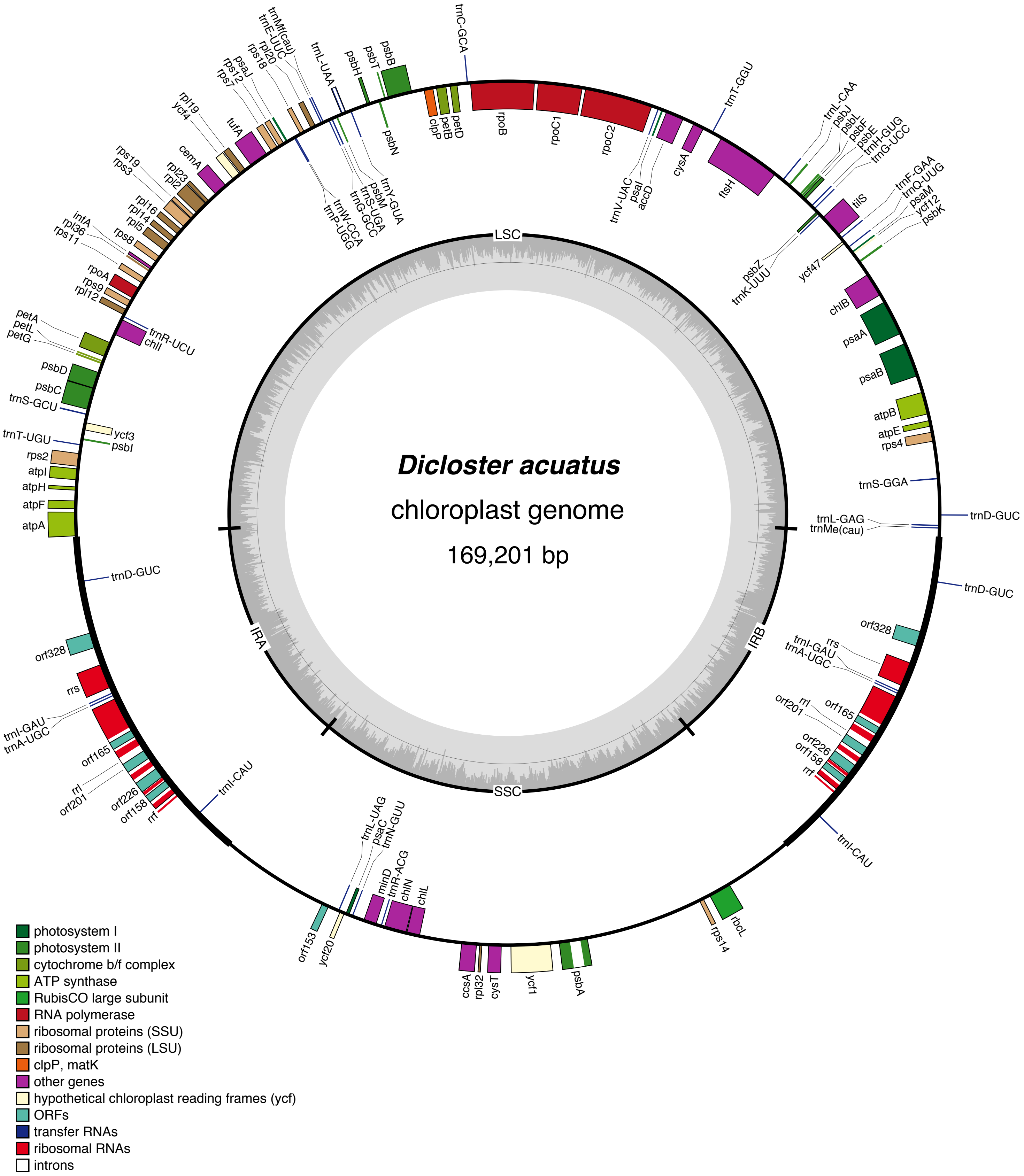
Dicloster acuatus
chloroplast genome
169,201 bp
LSC
IRA
IRB
SSC
trnC-GCA
rpoB
rpoC1
rpoC2
trnT-GGU
trnV-UAC
psaI
accD
cysA
ftsH
trnL-CAA
psbJ
psbL
psbF
psbE
trnH-GUG
trnG-UCC
tilS
trnF-GAA
trnQ-UUG
psaM
ycf12
psbK
psbZ
trnK-UUU
ycf47
chlB
psaA
psaB
atpB
atpE
rps4
trnS-GGA
trnD-GUC
trnL-GAG
trnMe(cau)
trnD-GUC
orf328
rrs
trnI-GAU
trnA-UGC
orf165
rrl
orf201
orf226
orf158
rrf
trnI-CAU
rbcL
rps14
psbA
ycf1
cysT
rpl32
ccsA
chlL
chlN
trnR-ACG
minD
trnN-GUU
psaC
trnL-UAG
ycf20
orf153
trnI-CAU
rrf
orf158
orf226
orf201
rrl
orf165
trnA-UGC
trnI-GAU
rrs
orf328
trnD-GUC
atpA
atpF
atpH
atpI
rps2
trnT-UGU
psbI
ycf3
trnS-GCU
psbC
psbD
petG
petL
petA
trnR-UCU
chlI
rpl12
rps9
rpoA
rps11
rpl36
infA
rps8
rpl5
rpl14
rpl16
rps3
rps19
rpl2
rpl23
cemA
ycf4
rpl19
tufA
rps7
rps12
psaJ
rps18
rpl20
trnE-UUC
trnMf(cau)
trnW-CCA
trnP-UGG
trnG-GCC
trnS-UGA
psbM
trnY-GUA
trnL-UAA
psbH
psbT
psbN
psbB
clpP
petB
petD
photosystem I
photosystem II
cytochrome b/f complex
ATP synthase
RubisCO large subunit
RNA polymerase
ribosomal proteins (SSU)
ribosomal proteins (LSU)
clpP, matK
other genes
hypothetical chloroplast reading frames (ycf)
ORFs
transfer RNAs
ribosomal RNAs
introns

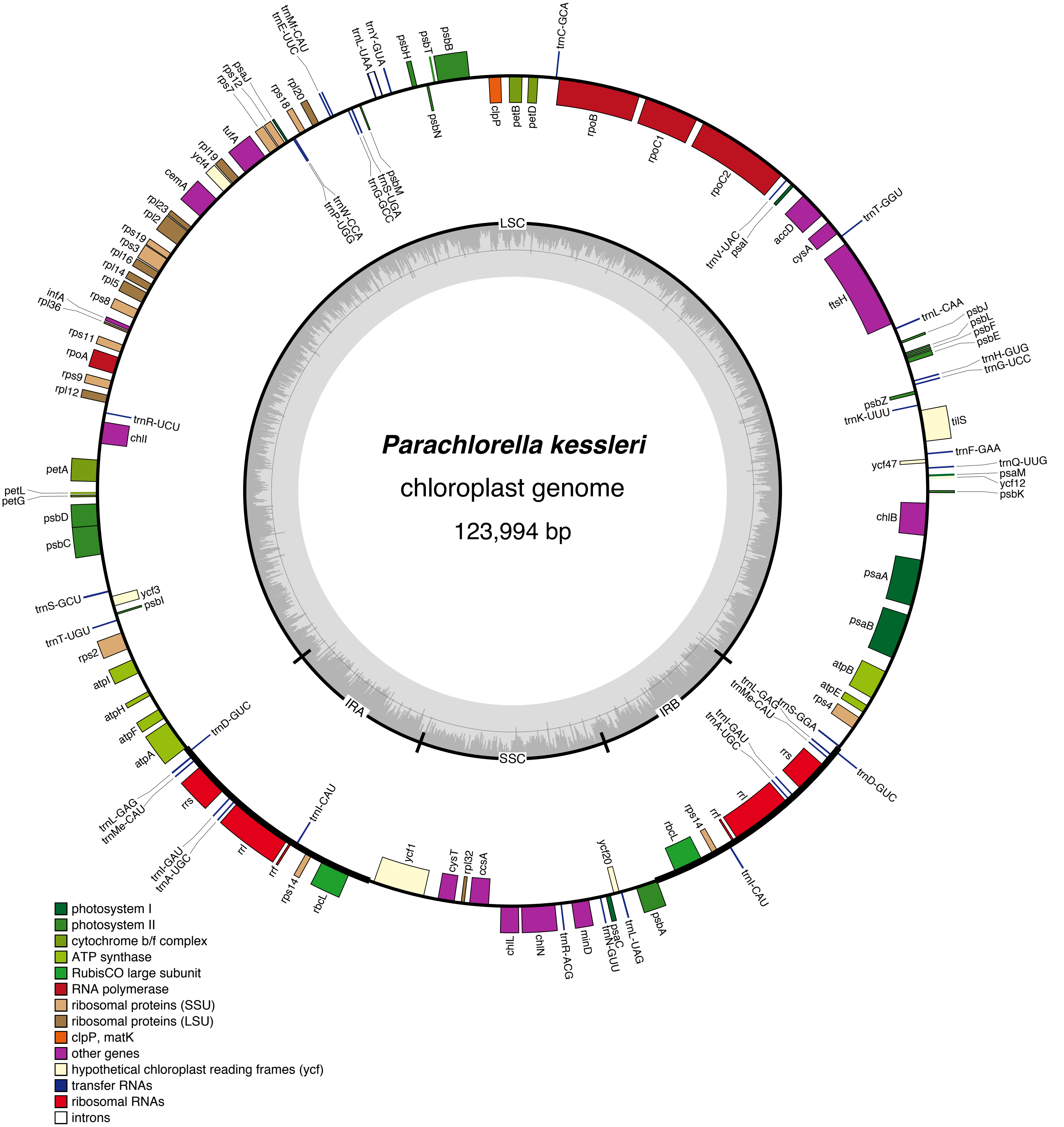

Parachlorella kessleri
chloroplast genome
123,994 bp
LSC
IRA
SSC
IRB
trnC-GCA
rpoB
rpoC1
rpoC2
trnV-UAC
psaI
accD
cysA
trnT-GGU
ftsH
trnL-CAA
psbJ
psbL
psbF
psbE
trnH-GUG
trnG-UCC
psbZ
trnK-UUU
tilS
trnF-GAA
ycf47
trnQ-UUG
psaM
ycf12
psbK
chlB
psaA
psaB
atpB
atpE
rps4
trnL-GAG
trnMe-CAU
trnS-GGA
trnI-GAU
trnA-UGC
rrs
trnD-GUC
rrl
rrf
rps14
rbcL
trnI-CAU
ycf20
psbA
trnL-UAG
psaC
trnN-GUU
minD
trnR-ACG
chlN
chlL
ccsA
rpl32
cysT
ycf1
trnI-CAU
rbcL
rps14
rrf
rrl
trnI-GAU
trnA-UGC
rrs
trnL-GAG
trnMe-CAU
trnD-GUC
atpA
atpF
atpH
atpI
rps2
trnT-UGU
trnS-GCU
ycf3
psbI
psbC
psbD
petG
petL
petA
chlI
trnR-UCU
rpl12
rps9
rpoA
rps11
rpl36
infA
rps8
rpl5
rpl14
rpl16
rps3
rps19
rpl2
rpl23
cemA
ycf4
rpl19
tufA
rps7
rps12
psaJ
rps18
rpl20
trnE-UUC
trnMf-CAU
trnW-CCA
trnP-UGG
trnL-UAA
trnY-GUA
trnG-GCC
trnS-UGA
psbM
psbH
psbT
psbN
psbB
clpP
petB
petD
photosystem I
photosystem II
cytochrome b/f complex
ATP synthase
RubisCO large subunit
RNA polymerase
ribosomal proteins (SSU)
ribosomal proteins (LSU)
clpP, matK
other genes
hypothetical chloroplast reading frames (ycf)
transfer RNAs
ribosomal RNAs
introns

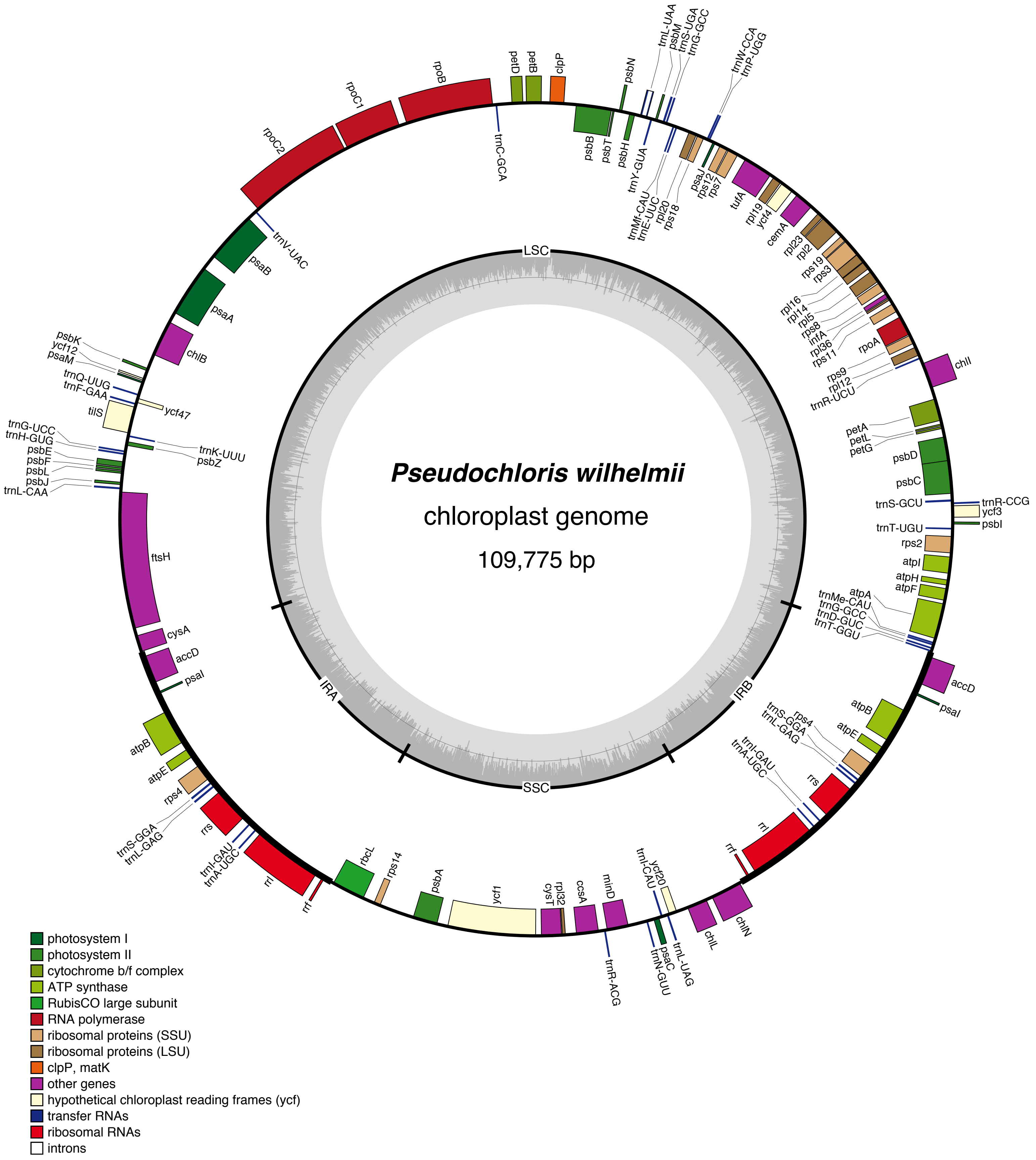
Pseudochloris wilhelmii
chloroplast genome
109,775 bp
LSC
IRA
IRB
SSC
photosystem I
photosystem II
cytochrome b/f complex
ATP synthase
RubisCO large subunit
RNA polymerase
ribosomal proteins (SSU)
ribosomal proteins (LSU)
clpP, matK
other genes
hypothetical chloroplast reading frames (ycf)
transfer RNAs
ribosomal RNAs
introns

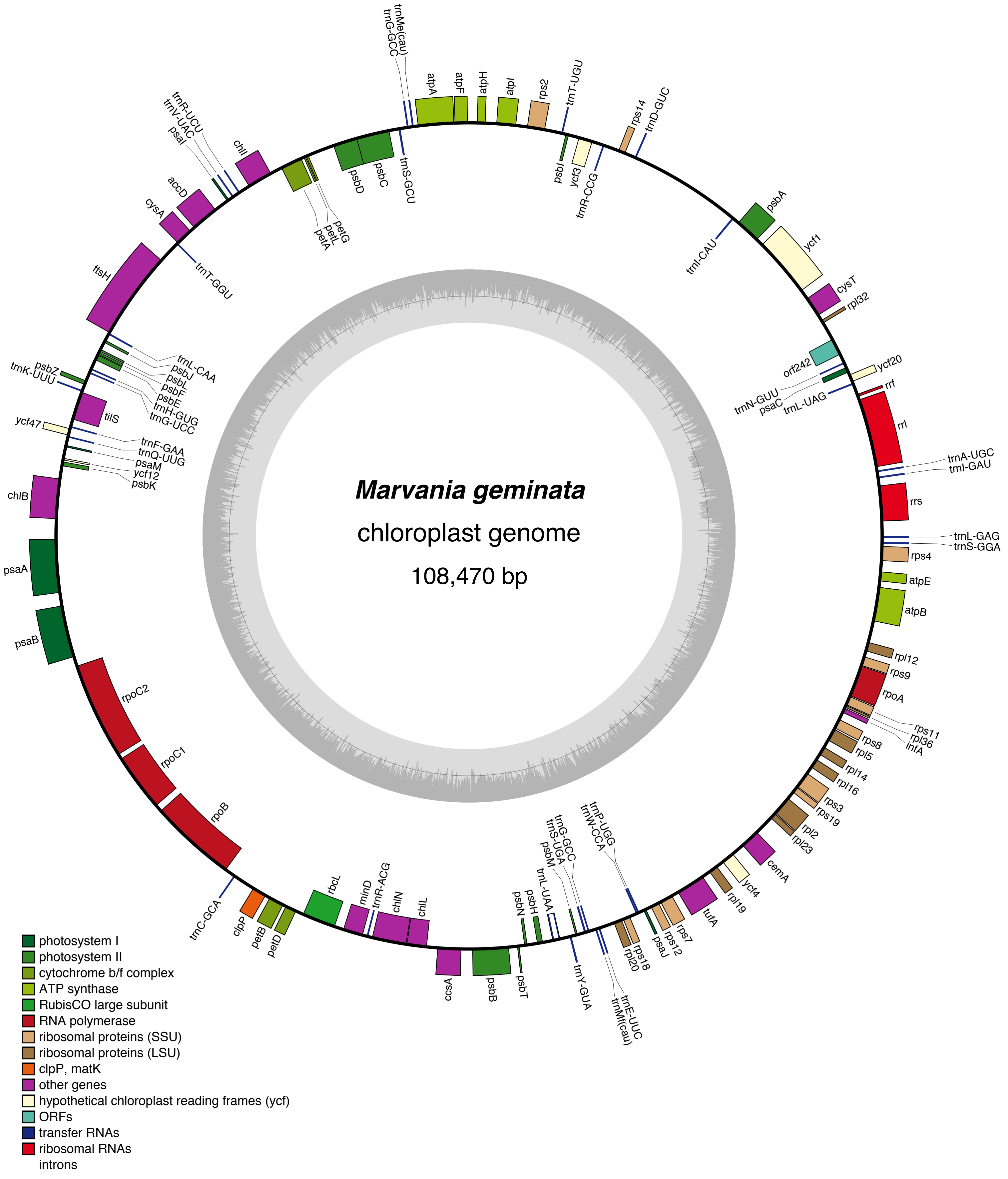
Marvania geminata
chloroplast genome
108,470 bp
trnMe(cau)
trnG-GCC
atpA
atpF
atpH
atpI
rps2
trnT-UGU
rps14
trnD-GUC
psbI
ycf3
trnR-CCG
psbD
psbC
trnS-GCU
petG
petL
petA
chlI
trnR-UCU
trnV-UAC
psaI
accD
cysA
trnT-GGU
ftsH
trnL-CAA
psbJ
psbL
psbF
psbE
trnH-GUG
trnG-UCC
psbZ
trnK-UUU
tilS
ycf47
trnF-GAA
trnQ-UUG
psaM
ycf12
psbK
chlB
psaA
psaB
rpoC2
rpoC1
rpoB
trnC-GCA
clpP
petB
petD
rbcL
minD
trnR-ACG
chlN
chlL
ccsA
psbB
psbT
psbN
psbH
trnL-UAA
psbM
trnS-UGA
trnG-GCC
trnP-UGG
trnW-CCA
trnY-GUA
trnE-UUC
trnMf(cau)
rps18
rpl20
psaJ
rps12
rps7
tufA
rpl19
ycf4
cemA
rpl23
rpl2
rps19
rps3
rpl16
rpl14
rpl5
rps8
infA
rpl36
rps11
rpoA
rps9
rpl12
atpB
atpE
rps4
trnS-GGA
trnL-GAG
rrs
trnI-GAU
trnA-UGC
rrl
rrf
ycf20
trnL-UAG
psaC
trnN-GUU
orf242
rpl32
cysT
ycf1
psbA
trnI-CAU
photosystem I
photosystem II
cytochrome b/f complex
ATP synthase
RubisCO large subunit
RNA polymerase
ribosomal proteins (SSU)
ribosomal proteins (LSU)
clpP, matK
other genes
hypothetical chloroplast reading frames (ycf)
ORFs
transfer RNAs
ribosomal RNAs
introns

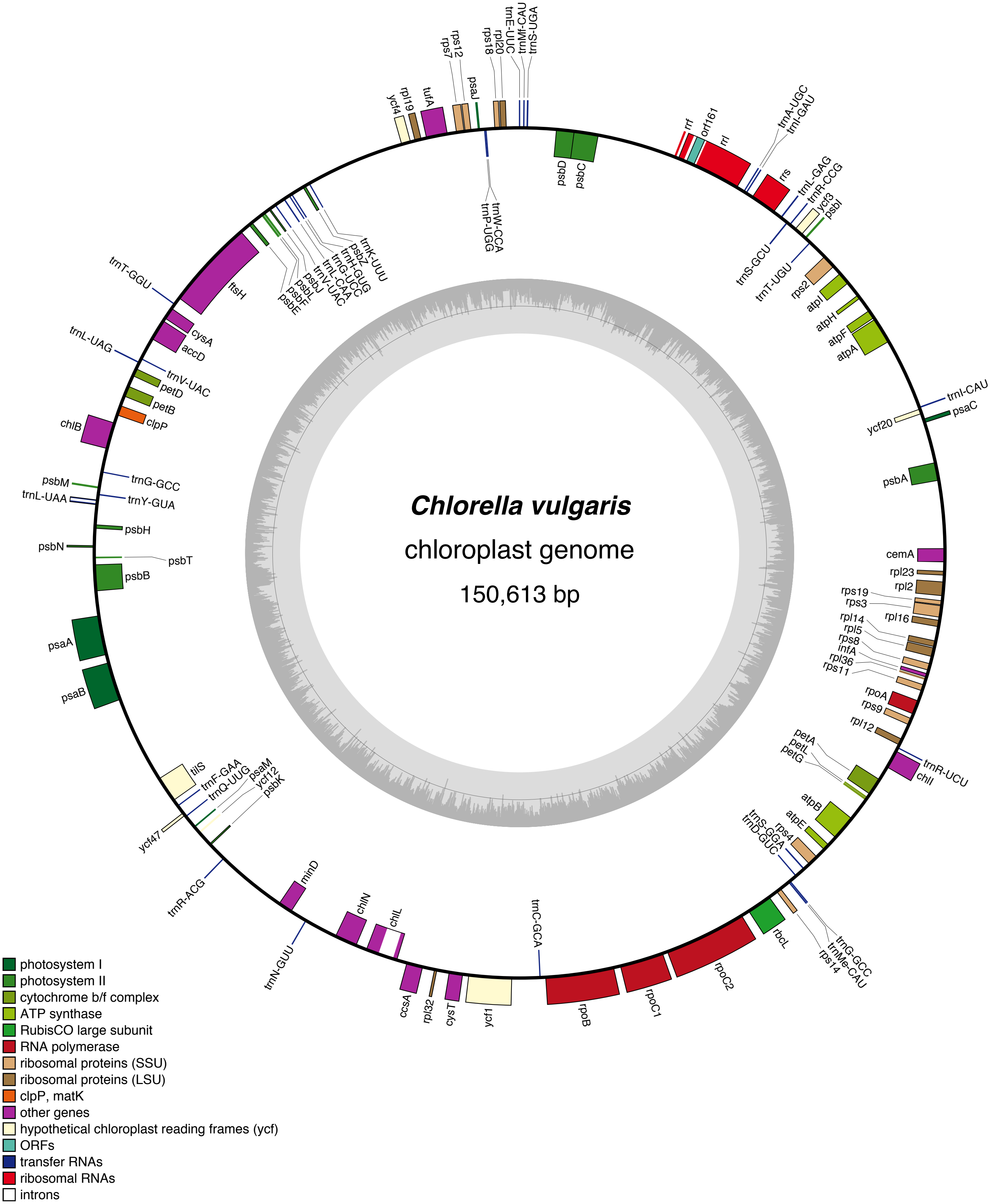

Chlorella vulgaris
chloroplast genome
150,613 bp
ycf4
rpl19
tufA
rps7
rps12
psaJ
rps18
rpl20
trnE-UUC
trnMf-CAU
trnS-UGA
trnW-CCA
trnP-UGG
psbD
psbC
rrf
orf161
rrl
trnA-UGC
trnI-GAU
rrs
trnL-GAG
trnR-CCG
ycf3
psbI
trnS-GCU
trnT-UGU
rps2
atpI
atpH
atpF
atpA
trnI-CAU
psaC
ycf20
psbA
cemA
rpl23
rpl2
rps19
rps3
rpl16
rpl14
rpl5
rps8
infA
rpl36
rps11
rpoA
rps9
rpl12
petA
petL
petG
trnR-UCU
chlI
atpB
atpE
rps4
trnS-GGA
trnD-GUC
rbcL
trnG-GCC
trnMe-CAU
rps14
rpoC2
rpoC1
rpoB
trnC-GCA
ycf1
cysT
rpl32
ccsA
chlL
chlN
trnN-GUU
minD
trnR-ACG
ycf47
tilS
trnF-GAA
trnQ-UUG
psaM
ycf12
psbK
psaB
psaA
psbB
psbT
psbN
psbH
trnL-UAA
trnY-GUA
psbM
trnG-GCC
chlB
clpP
petB
petD
trnV-UAC
trnL-UAG
accD
cysA
trnT-GGU
ftsH
trnK-UUU
psbZ
trnH-GUG
trnG-UCC
trnL-CAA
trnV-UAC
psbJ
psbL
psbF
psbE
photosystem I
photosystem II
cytochrome b/f complex
ATP synthase
RubisCO large subunit
RNA polymerase
ribosomal proteins (SSU)
ribosomal proteins (LSU)
clpP, matK
other genes
hypothetical chloroplast reading frames (ycf)
ORFs
transfer RNAs
ribosomal RNAs
introns

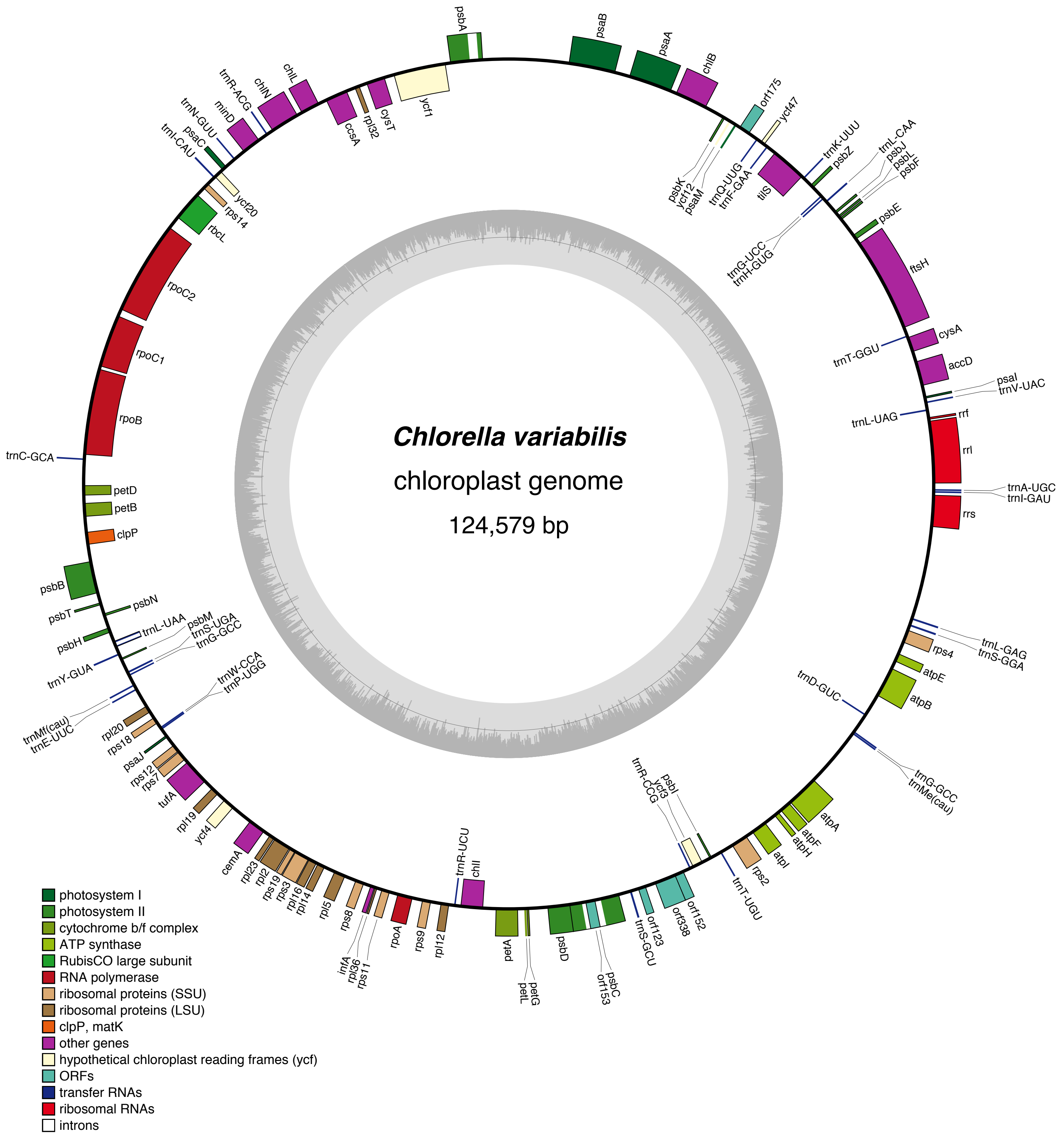

Chlorella variabilis
chloroplast genome
124,579 bp
psbA
psaB
psaA
chlB
orf175
ycf47
trnK-UUU
psbZ
trnL-CAA
psbJ
psbL
psbF
psbK
ycf12
psaM
trnQ-UUG
trnF-GAA
tilS
trnG-UCC
trnH-GUG
psbE
ftsH
cysA
trnT-GGU
accD
psaI
trnV-UAC
trnL-UAG
rrf
rrl
trnA-UGC
trnI-GAU
rrs
trnL-GAG
trnS-GGA
rps4
atpE
trnD-GUC
atpB
trnG-GCC
trnMe(cau)
atpA
atpF
atpH
atpI
rps2
trnT-UGU
psbI
ycf3
trnR-CCG
orf152
orf338
orf123
trnS-GCU
psbC
orf153
psbD
petG
petL
petA
chlI
trnR-UCU
rpl12
rps9
rpoA
infA
rpl36
rps11
rps8
rpl5
rpl14
rpl16
rps3
rps19
rpl2
rpl23
cemA
ycf4
rpl19
tufA
rps7
rps12
psaJ
rps18
rpl20
trnMf(cau)
trnE-UUC
trnW-CCA
trnP-UGG
trnY-GUA
psbH
trnL-UAA
psbM
trnS-UGA
trnG-GCC
psbT
psbN
psbB
clpP
petB
petD
trnC-GCA
rpoB
rpoC1
rpoC2
rbcL
rps14
ycf20
trnI-CAU
psaC
trnN-GUU
minD
trnR-ACG
chlN
chlL
ccsA
rpl32
cysT
ycf1
photosystem I
photosystem II
cytochrome b/f complex
ATP synthase
RubisCO large subunit
RNA polymerase
ribosomal proteins (SSU)
ribosomal proteins (LSU)
clpP, matK
other genes
hypothetical chloroplast reading frames (ycf)
ORFs
transfer RNAs
ribosomal RNAs
introns

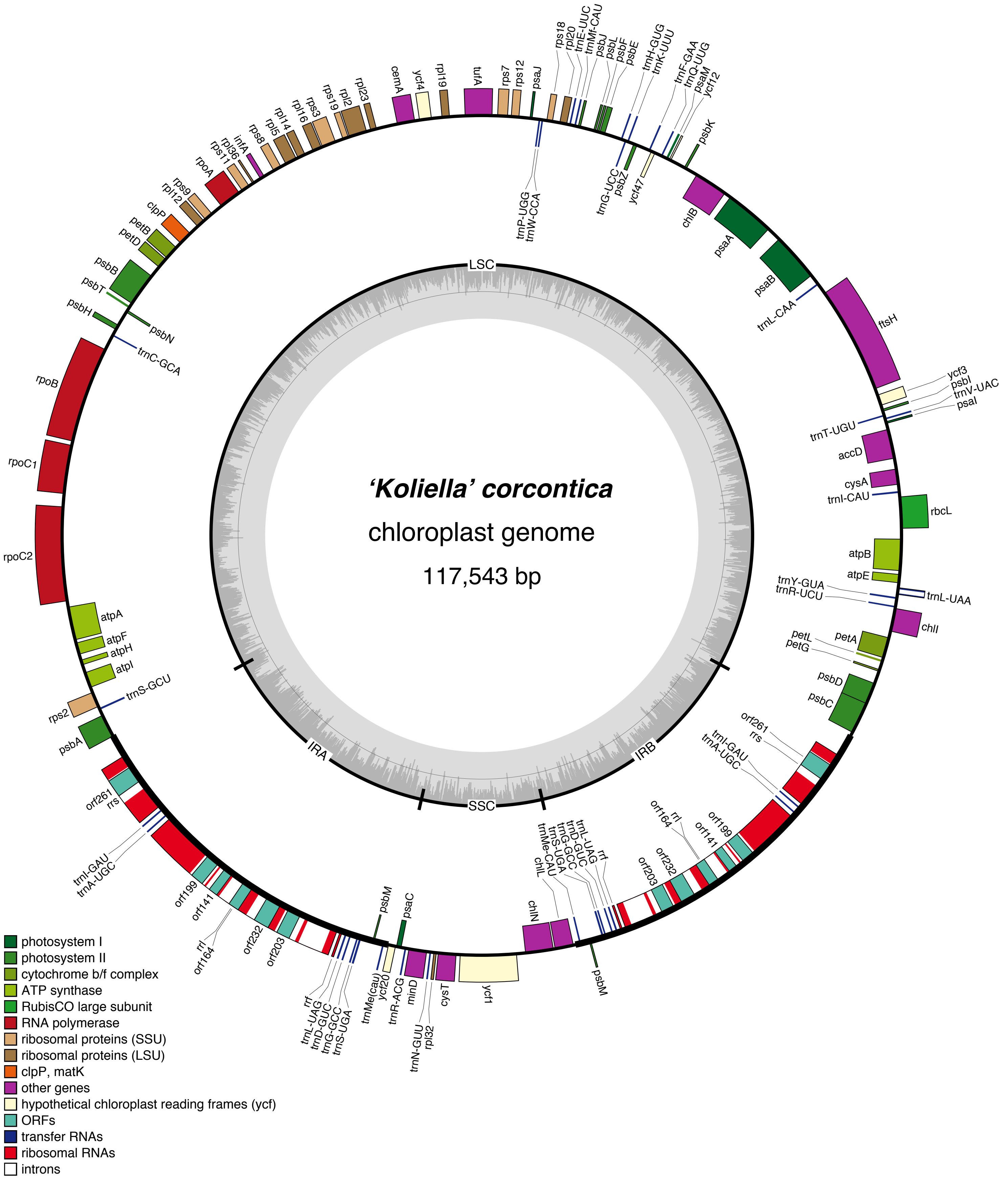

‘Koliella’ corcontica
chloroplast genome
117,543 bp
LSC
IRA
SSC
IRB
cemA
ycf4
rpl19
tufA
rps7
rps12
psaJ
rps18
rpl20
trnE-UUC
trnMf-CAU
psbJ
psbL
psbF
psbE
trnH-GUG
trnK-UUU
trnF-GAA
trnQ-UUG
psaM
ycf12
psbK
trnP-UGG
trnW-CCA
trnG-UCC
psbZ
ycf47
chlB
psaA
psaB
trnL-CAA
ftsH
ycf3
psbI
trnV-UAC
psaI
trnT-UGU
accD
cysA
trnI-CAU
rbcL
atpB
atpE
trnY-GUA
trnR-UCU
trnL-UAA
chlI
petA
petL
petG
psbD
psbC
orf261
rrs
trnI-GAU
trnA-UGC
orf164
rrl
orf141
orf199
orf203
orf232
trnL-UAG
trnD-GUC
trnG-GCC
trnS-UGA
trnMe-CAU
chlL
rrf
chlN
psbM
ycf1
cysT
rpl32
trnN-GUU
minD
trnR-ACG
ycf20
trnMe(cau)
psaC
rps2
psbA
trnS-GCU
atpA
atpF
atpH
atpI
rpoC2
rpoC1
rpoB
trnC-GCA
psbH
psbN
psbT
psbB
petD
petB
clpP
rpl12
rps9
rpoA
rps11
rpl36
infA
rps8
rpl5
rpl14
rpl16
rps3
rps19
rpl2
rpl23
photosystem I
photosystem II
cytochrome b/f complex
ATP synthase
RubisCO large subunit
RNA polymerase
ribosomal proteins (SSU)
ribosomal proteins (LSU)
clpP, matK
other genes
hypothetical chloroplast reading frames (ycf)
ORFs
transfer RNAs
ribosomal RNAs
introns

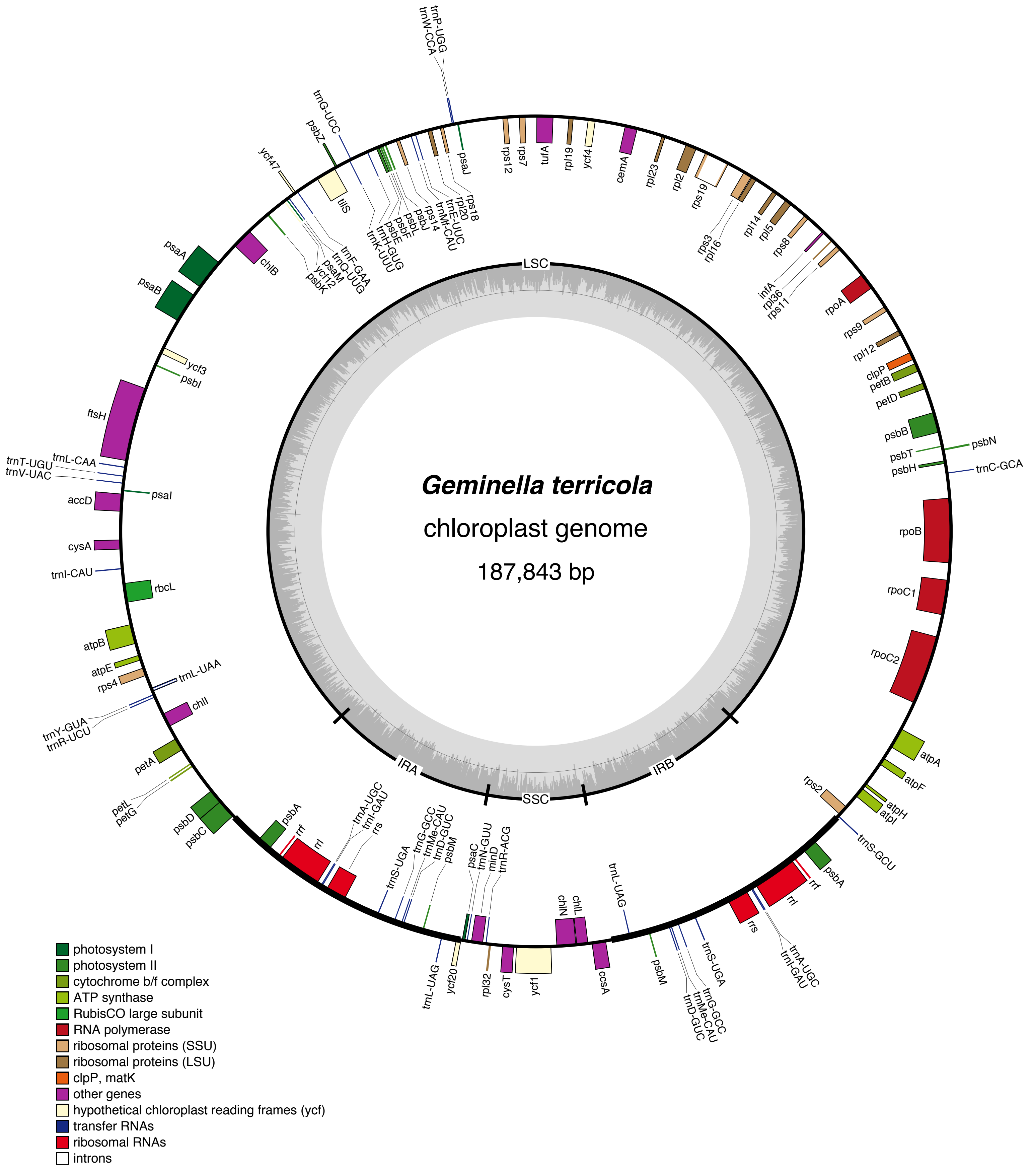

Geminella terricola
chloroplast genome
187,843 bp
LSC
IRA
SSC
IRB
trnP-UGG
trnW-CCA
trnG-UCC
psbZ
ycf47
psaJ
rps12
rps7
tufA
rpl19
ycf4
cemA
rpl23
rpl2
rps19
rps3
rpl16
rpl14
rpl5
rps8
infA
rpl36
rps11
rpoA
rps9
rpl12
clpP
petB
petD
psbB
psbN
psbT
psbH
trnC-GCA
rpoB
rpoC1
rpoC2
atpA
atpF
atpH
atpI
rps2
trnS-GCU
psbA
rrf
rrl
rrs
trnA-UGC
trnI-GAU
trnS-UGA
trnG-GCC
trnMe-CAU
trnD-GUC
psbM
trnL-UAG
chlN
chlL
ccsA
ycf1
cysT
rpl32
ycf20
psaC
trnN-GUU
minD
trnR-ACG
psbD
psbC
petL
petG
petA
trnY-GUA
trnR-UCU
chlI
trnL-UAA
rps4
atpE
atpB
rbcL
trnI-CAU
cysA
accD
psaI
trnT-UGU
trnV-UAC
trnL-CAA
ftsH
psbI
ycf3
psaB
psaA
chlB
psbK
ycf12
psaM
trnQ-UUG
trnF-GAA
tilS
trnK-UUU
trnH-GUG
psbE
psbF
psbL
psbJ
rps14
trnMf-CAU
trnE-UUC
rpl20
rps18
photosystem I
photosystem II
cytochrome b/f complex
ATP synthase
RubisCO large subunit
RNA polymerase
ribosomal proteins (SSU)
ribosomal proteins (LSU)
clpP, matK
other genes
hypothetical chloroplast reading frames (ycf)
transfer RNAs
ribosomal RNAs
introns

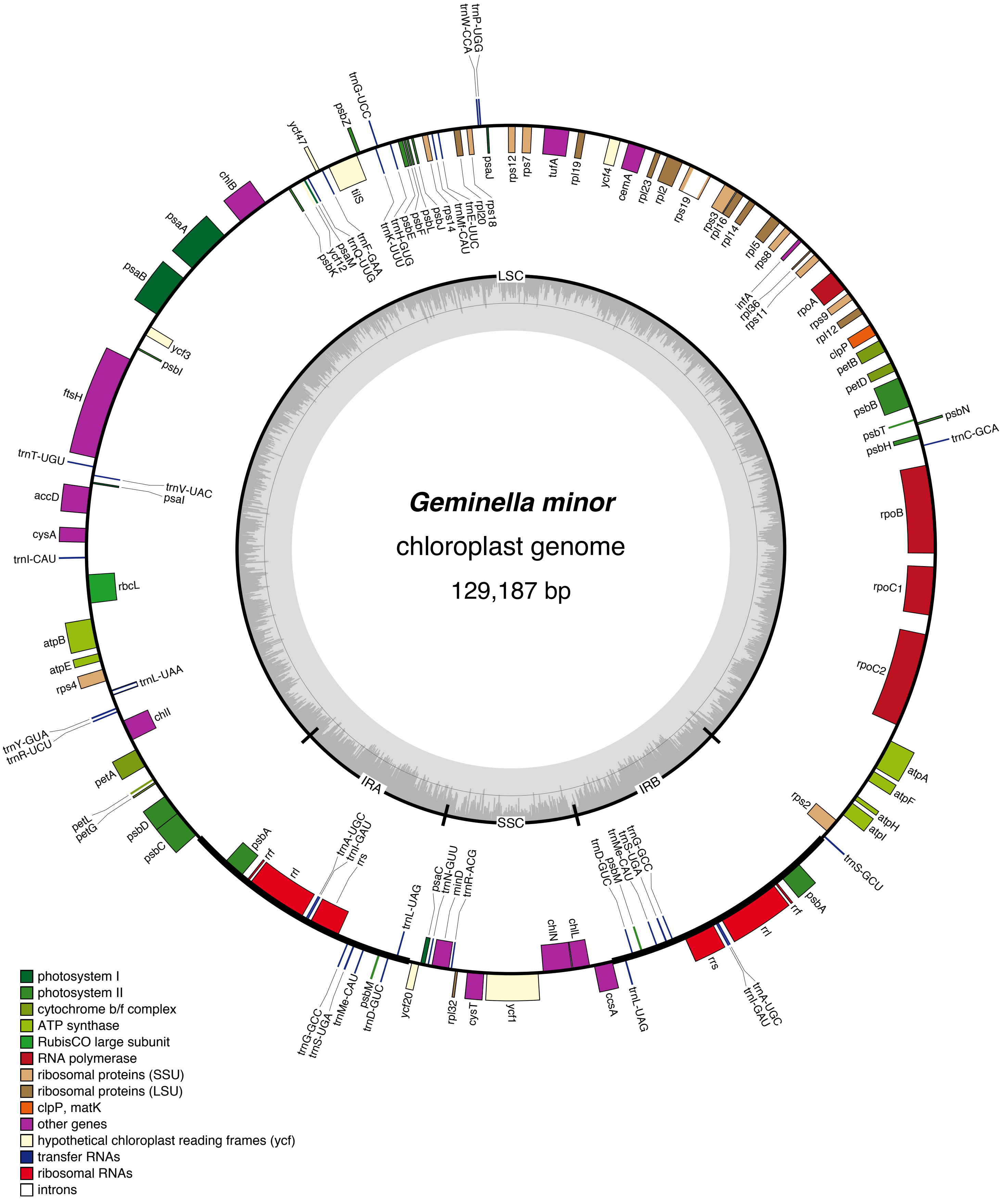
Geminella minor
chloroplast genome
129,187 bp
LSC
IRA
IRB
SSC
trnP-UGG
trnW-CCA
trnG-UCC
psbZ
ycf47
tilS
chlB
psaA
psaB
ycf3
psbI
ftsH
trnT-UGU
trnV-UAC
psaI
accD
cysA
trnI-CAU
rbcL
atpB
atpE
rps4
trnL-UAA
trnY-GUA
trnR-UCU
chlI
petA
petL
petG
psbD
psbC
psbA
rrf
rrl
trnA-UGC
trnI-GAU
rrs
trnG-GCC
trnS-UGA
trnMe-CAU
psbM
trnD-GUC
trnL-UAG
ycf20
psaC
trnN-GUU
minD
trnR-ACG
rpl32
cysT
ycf1
chlN
chlL
ccsA
trnS-GCU
rps2
atpA
atpF
atpH
atpI
rpoC2
rpoC1
rpoB
trnC-GCA
psbN
psbT
psbH
psbB
petD
petB
clpP
rpl12
rps9
rpoA
infA
rpl36
rps11
rps8
rpl5
rpl14
rpl16
rps3
rps19
rpl2
rpl23
cemA
ycf4
rpl19
tufA
rps7
rps12
psaJ
rps18
rpl20
trnE-UUC
trnMf-CAU
rps14
psbJ
psbL
psbF
psbE
trnH-GUG
trnK-UUU
trnF-GAA
trnQ-UUG
psaM
ycf12
psbK
photosystem I
photosystem II
cytochrome b/f complex
ATP synthase
RubisCO large subunit
RNA polymerase
ribosomal proteins (SSU)
ribosomal proteins (LSU)
clpP, matK
other genes
hypothetical chloroplast reading frames (ycf)
transfer RNAs
ribosomal RNAs
introns

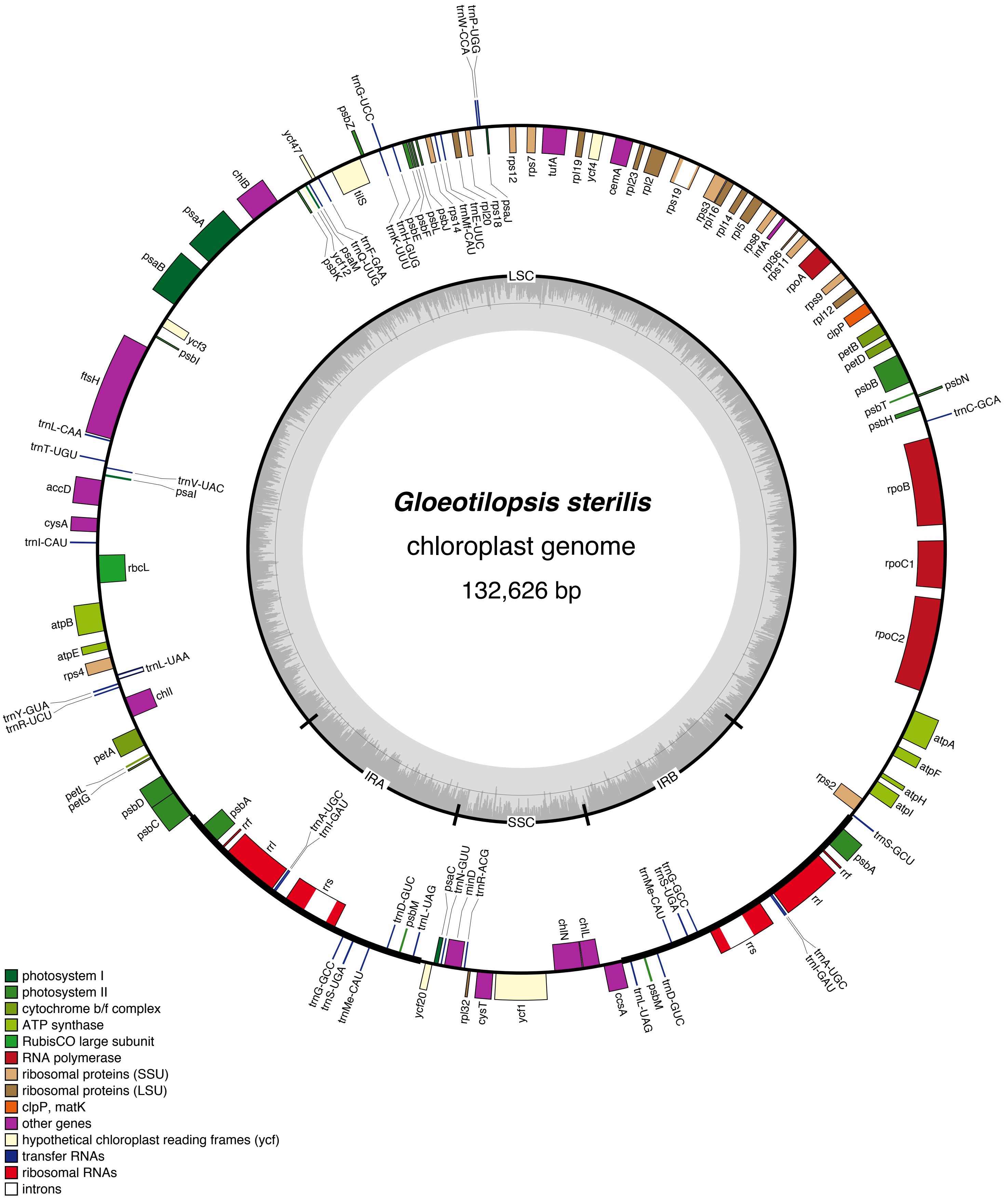
Gloeotilopsis sterilis
chloroplast genome
132,626 bp
LSC
IRA
SSC
IRB
trnP-UGG
trnW-CCA
trnG-UCC
psbZ
ycf47
tilS
chlB
psaA
psaB
ycf3
psbI
ftsH
trnL-CAA
trnT-UGU
trnV-UAC
psaI
accD
cysA
trnI-CAU
rbcL
atpB
atpE
rps4
trnL-UAA
trnY-GUA
trnR-UCU
chlI
petA
petL
petG
psbD
psbC
psbA
rrf
rrl
trnA-UGC
trnI-GAU
rrs
trnG-GCC
trnS-UGA
trnMe-CAU
trnD-GUC
psbM
trnL-UAG
ycf20
psaC
trnN-GUU
minD
trnR-ACG
rpl32
cysT
ycf1
chlN
chlL
ccsA
trnL-UAG
psbM
trnD-GUC
trnMe-CAU
trnS-UGA
trnG-GCC
rrs
trnA-UGC
trnI-GAU
rrl
rrf
psbA
trnS-GCU
rps2
atpI
atpH
atpF
atpA
rpoC2
rpoC1
rpoB
trnC-GCA
psbN
psbT
psbH
psbB
petD
petB
clpP
rpl12
rps9
rpoA
rps11
rpl36
infA
rps8
rpl5
rpl14
rpl16
rps3
rps19
rpl2
rpl23
cemA
ycf4
rpl19
tufA
rps7
rps12
psaJ
rps18
rpl20
trnE-UUC
trnMf-CAU
rps14
psbJ
psbL
psbF
psbE
trnH-GUG
trnK-UUU
trnF-GAA
trnQ-UUG
psaM
ycf12
psbK
photosystem I
photosystem II
cytochrome b/f complex
ATP synthase
RubisCO large subunit
RNA polymerase
ribosomal proteins (SSU)
ribosomal proteins (LSU)
clpP, matK
other genes
hypothetical chloroplast reading frames (ycf)
transfer RNAs
ribosomal RNAs
introns

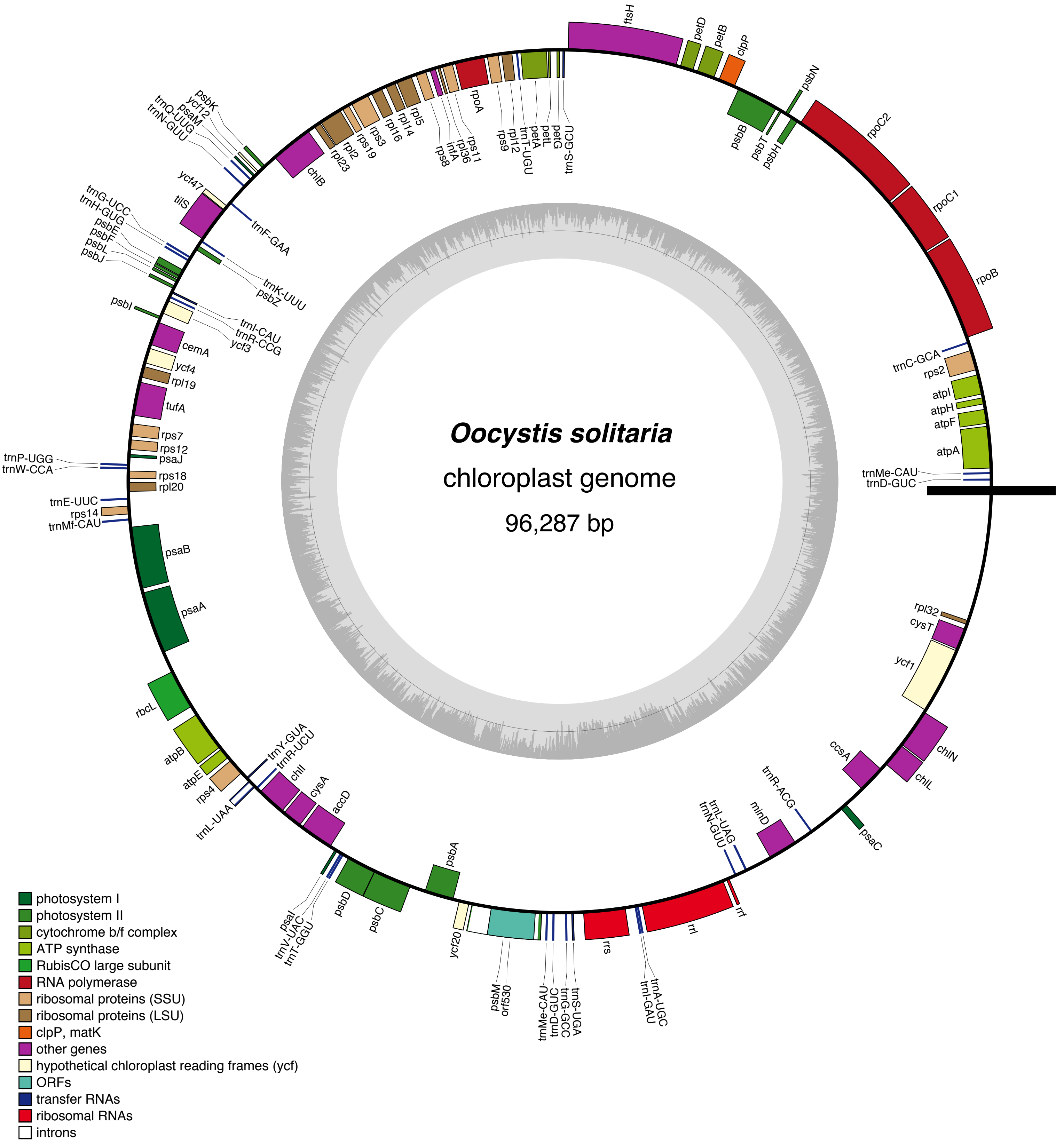

Oocystis solitaria
chloroplast genome
96,287 bp
ftsH
petD
petB
clpP
psbN
rpoC2
rpoC1
rpoB
trnC-GCA
rps2
atpI
atpH
atpF
atpA
trnMe-CAU
trnD-GUC
rpl32
cysT
ycf1
chlN
chlL
ccsA
psaC
trnR-ACG
minD
trnL-UAG
trnN-GUU
rrf
rrl
trnA-UGC
trnI-GAU
rrs
trnS-UGA
trnG-GCC
trnD-GUC
trnMe-CAU
orf530
psbM
ycf20
psbA
psbC
psbD
trnT-GGU
trnV-UAC
psaI
accD
cysA
chlI
trnR-UCU
trnY-GUA
trnL-UAA
rps4
atpE
atpB
rbcL
psaA
psaB
trnMf-CAU
rps14
trnE-UUC
rpl20
rps18
trnW-CCA
trnP-UGG
psaJ
rps12
rps7
tufA
rpl19
ycf4
cemA
ycf3
trnR-CCG
trnI-CAU
psbI
psbJ
psbL
psbF
psbE
trnH-GUG
trnG-UCC
psbZ
trnK-UUU
trnF-GAA
tilS
ycf47
trnN-GUU
trnQ-UUG
psaM
ycf12
psbK
chlB
rpl23
rpl2
rps19
rps3
rpl16
rpl14
rpl5
rps8
infA
rpl36
rps11
rpoA
rps9
rpl12
petA
trnT-UGU
petL
petG
trnS-GCU
psbB
psbT
psbH
photosystem I
photosystem II
cytochrome b/f complex
ATP synthase
RubisCO large subunit
RNA polymerase
ribosomal proteins (SSU)
ribosomal proteins (LSU)
clpP, matK
other genes
hypothetical chloroplast reading frames (ycf)
ORFs
transfer RNAs
ribosomal RNAs
introns

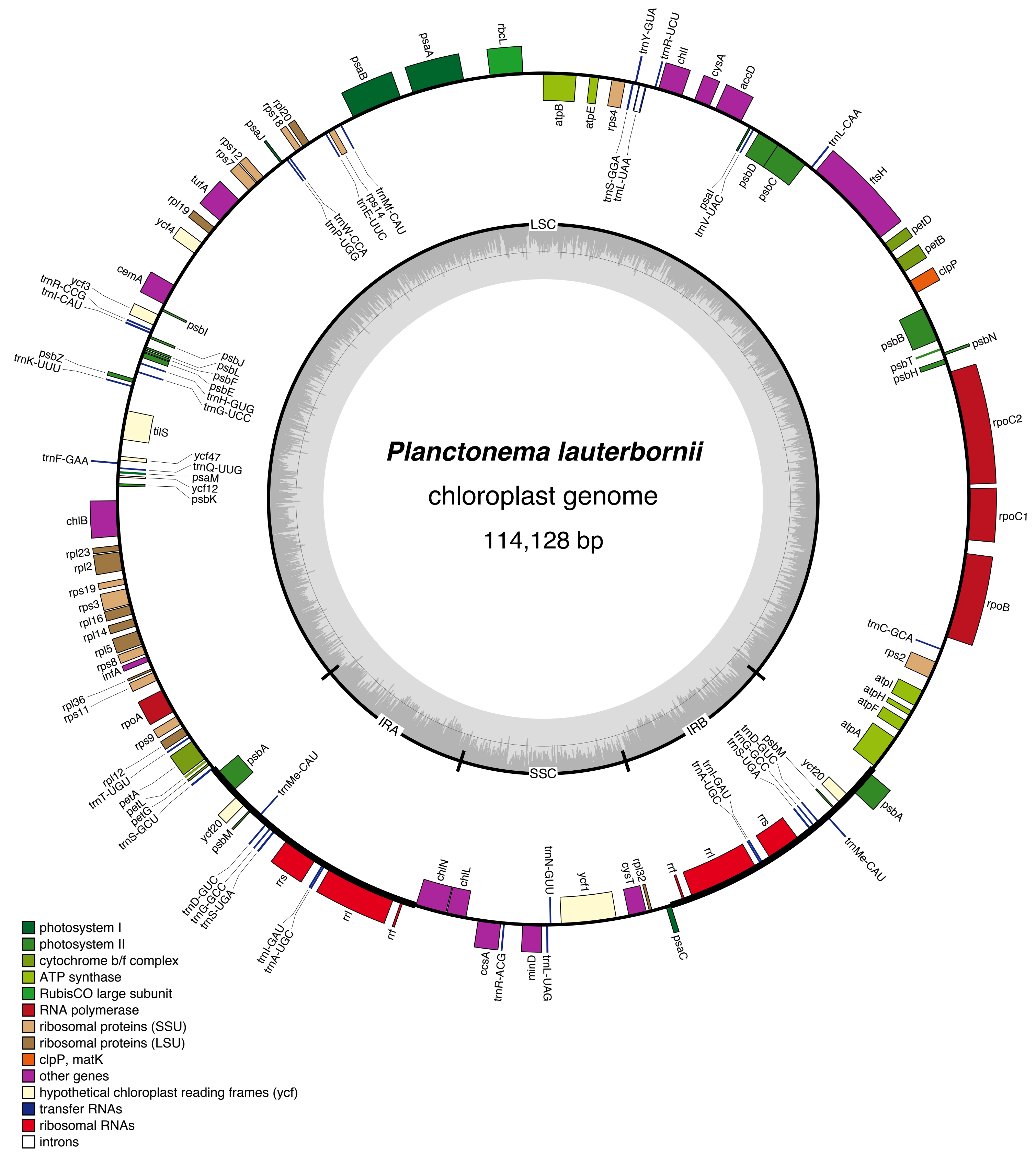

Planctonema lauterbornii
chloroplast genome
114,128 bp
LSC
IRA
IRB
SSC
psaB
psaA
rbcL
atpB
atpE
rps4
trnY-GUA
trnR-UCU
chlI
cysA
accD
trnS-GGA
trnL-UAA
psaI
trnV-UAC
psbD
psbC
trnL-CAA
ftsH
petD
petB
clpP
psbB
psbN
psbT
psbH
rpoC2
rpoC1
rpoB
trnC-GCA
rps2
atpI
atpH
atpF
atpA
psbM
trnD-GUC
trnG-GCC
trnS-UGA
ycf20
psbA
trnMe-CAU
trnI-GAU
trnA-UGC
rrs
rrl
rrf
psaC
rpl32
cysT
ycf1
trnN-GUU
minD
trnL-UAG
ccsA
trnR-ACG
chlN
chlL
tilS
chlB
rpl23
rpl2
rps19
rps3
rpl16
rpl14
rpl5
rps8
infA
rpl36
rps11
rpoA
rps9
rpl12
trnT-UGU
petA
petL
petG
trnS-GCU
trnF-GAA
ycf47
trnQ-UUG
psaM
ycf12
psbK
psbZ
trnK-UUU
psbJ
psbL
psbF
psbE
trnH-GUG
trnG-UCC
ycf3
trnR-CCG
trnI-CAU
psbI
cemA
ycf4
rpl19
tufA
rps7
rps12
psaJ
rps18
rpl20
trnW-CCA
trnP-UGG
trnE-UUC
rps14
trnMf-CAU
photosystem I
photosystem II
cytochrome b/f complex
ATP synthase
RubisCO large subunit
RNA polymerase
ribosomal proteins (SSU)
ribosomal proteins (LSU)
clpP, matK
other genes
hypothetical chloroplast reading frames (ycf)
transfer RNAs
ribosomal RNAs
introns

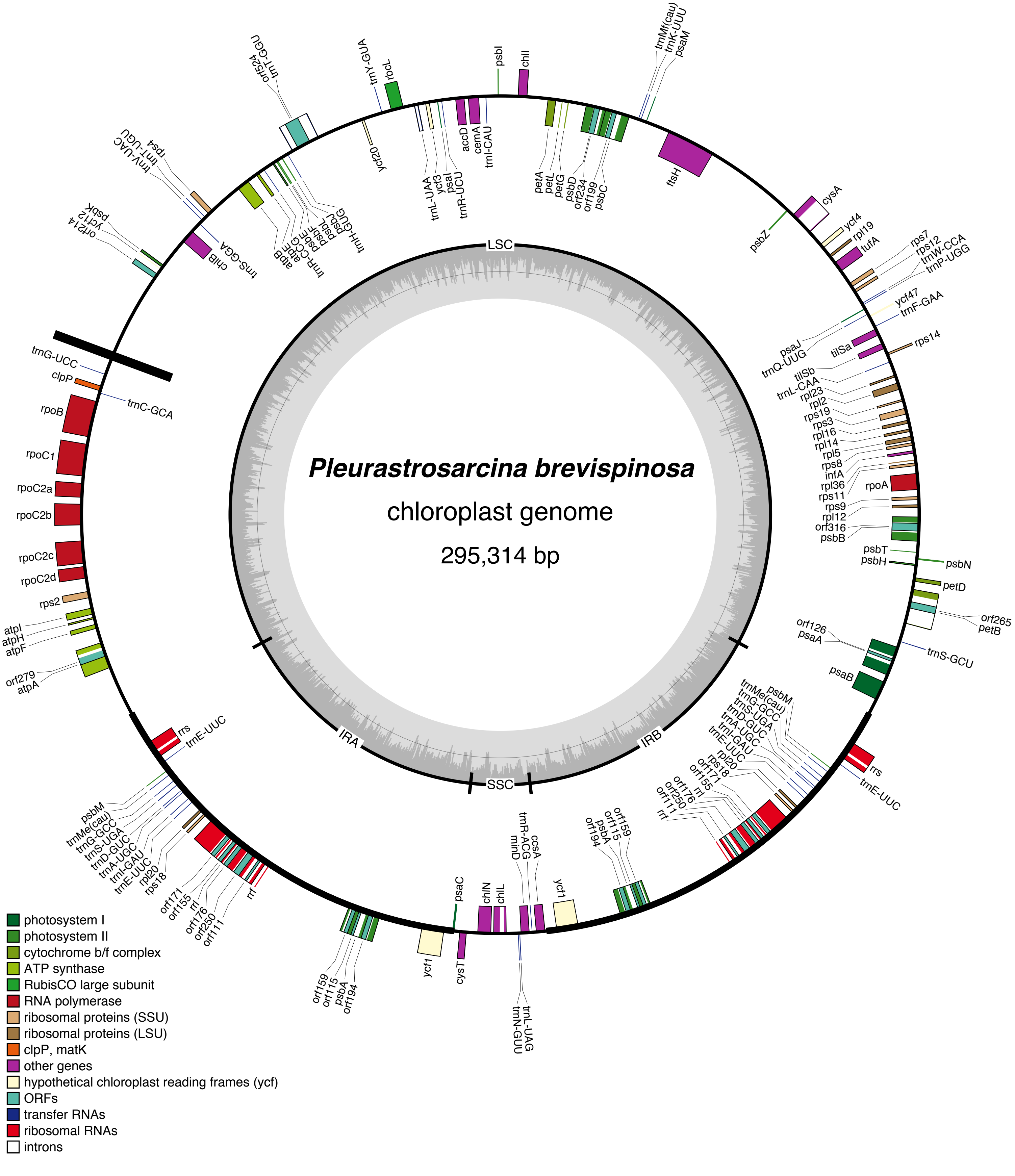

Pleurastrosarcina brevispinosa
chloroplast genome
295,314 bp
LSC
IRA
IRB
SSC
psbI
chlI
trnMf(cau)
trnK-UUU
psaM
ftsH
cysA
psbZ
ycf4
rpl19
tufA
rps7
rps12
trnW-CCA
trnP-UGG
ycf47
trnF-GAA
psaJ
trnQ-UUG
tilSa
rps14
tilSb
trnL-CAA
rpl23
rpl2
rps19
rps3
rpl16
rpl14
rpl5
rps8
infA
rpl36
rps11
rps9
rpl12
orf316
psbB
rpoA
psbT
psbH
psbN
petD
orf265
petB
orf126
psaA
trnS-GCU
psaB
psbM
trnMe(cau)
trnG-GCC
trnS-UGA
trnD-GUC
trnA-UGC
trnI-GAU
trnE-UUC
rpl20
rps18
orf171
orf155
rrl
orf176
orf250
orf111
rrf
rrs
trnE-UUC
orf159
orf115
psbA
orf194
trnR-ACG
minD
ccsA
ycf1
chlN
chlL
psaC
cysT
trnL-UAG
trnN-GUU
petA
petL
petG
psbD
orf234
orf199
psbC
accD
cemA
trnI-CAU
trnL-UAA
ycf3
psaI
trnR-UCU
ycf20
trnY-GUA
rbcL
orf524
trnT-GGU
trnH-GUG
psbJ
psbL
psbF
psbE
trnH-CCG
atpE
atpB
trnV-UAC
trnT-UGU
rps4
trnS-GGA
chlB
orf214
ycf12
psbK
trnG-UCC
clpP
trnC-GCA
rpoB
rpoC1
rpoC2a
rpoC2b
rpoC2c
rpoC2d
rps2
atpI
atpH
atpF
orf279
atpA
photosystem I
photosystem II
cytochrome b/f complex
ATP synthase
RubisCO large subunit
RNA polymerase
ribosomal proteins (SSU)
ribosomal proteins (LSU)
clpP, matK
other genes
hypothetical chloroplast reading frames (ycf)
ORFs
transfer RNAs
ribosomal RNAs
introns

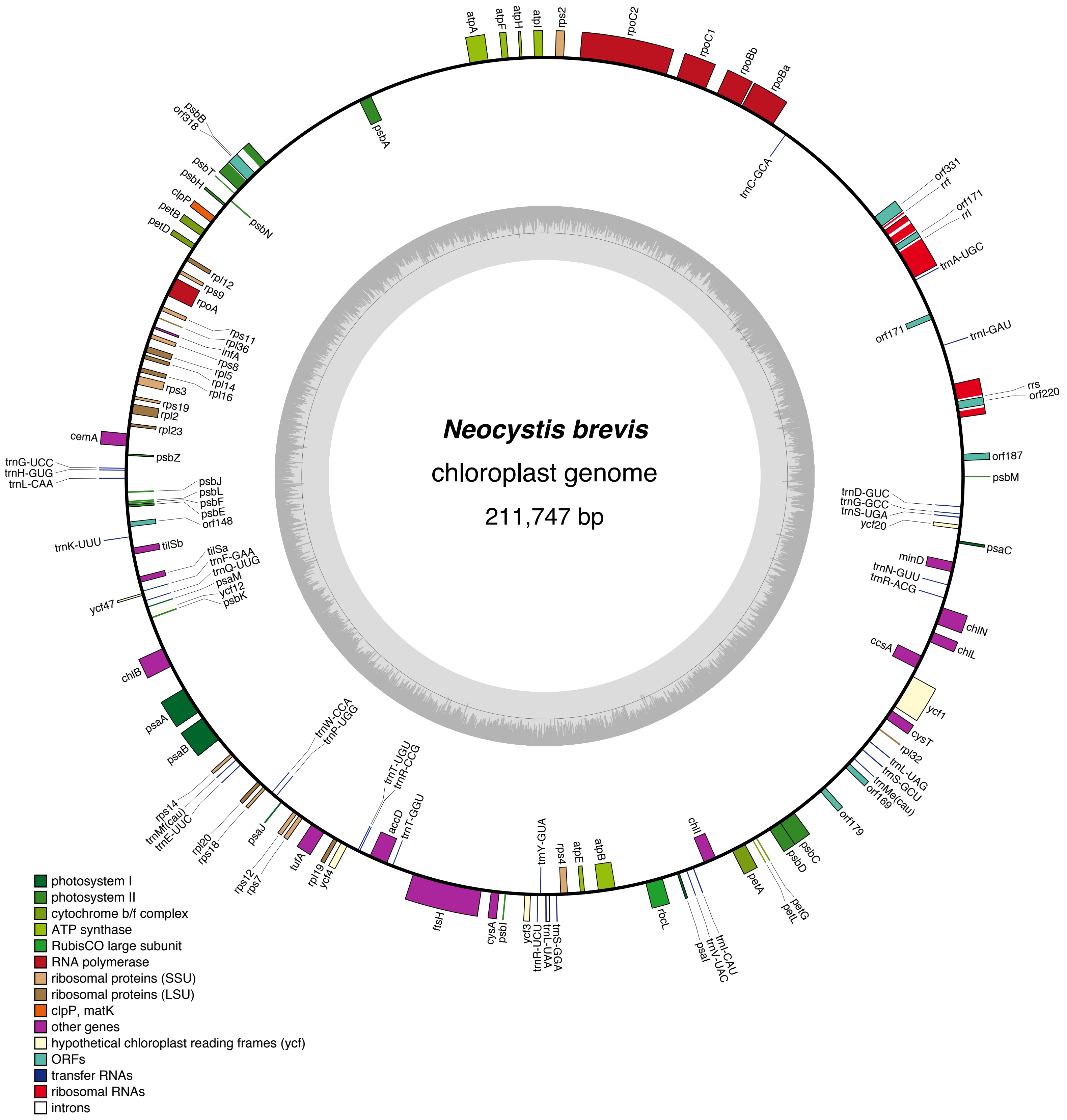

Neocystis brevis
chloroplast genome
211,747 bp
atpA
atpF
atpH
atpI
rps2
rpoC2
rpoC1
rpoBb
rpoBa
trnC-GCA
orf331
rrf
orf171
rrl
trnA-UGC
orf171
trnI-GAU
rrs
orf220
orf187
psbM
trnD-GUC
trnG-GCC
trnS-UGA
ycf20
psaC
minD
trnN-GUU
trnR-ACG
chlN
chlL
ccsA
ycf1
cysT
rpl32
trnL-UAG
trnS-GCU
trnMe(cau)
orf169
orf179
psbC
psbD
petG
petL
petA
chlI
trnI-CAU
trnV-UAC
psaI
rbcL
atpB
atpE
rps4
trnS-GGA
trnL-UAA
trnR-UCU
trnY-GUA
ycf3
psbI
cysA
ftsH
trnT-GGU
accD
trnR-CCG
trnT-UGU
ycf4
rpl19
tufA
rps7
rps12
psaJ
rps18
rpl20
trnP-UGG
trnW-CCA
trnE-UUC
trnMf(cau)
rps14
psaB
psaA
chlB
ycf47
psbK
ycf12
psaM
trnQ-UUG
trnF-GAA
tilSa
tilSb
trnK-UUU
orf148
psbE
psbF
psbL
psbJ
trnL-CAA
trnH-GUG
trnG-UCC
psbZ
cemA
rpl23
rpl2
rps19
rps3
rpl16
rpl14
rpl5
rps8
infA
rpl36
rps11
rpoA
rps9
rpl12
petD
petB
clpP
psbH
psbT
psbN
orf318
psbB
psbA
photosystem I
photosystem II
cytochrome b/f complex
ATP synthase
RubisCO large subunit
RNA polymerase
ribosomal proteins (SSU)
ribosomal proteins (LSU)
clpP, matK
other genes
hypothetical chloroplast reading frames (ycf)
ORFs
transfer RNAs
ribosomal RNAs
introns

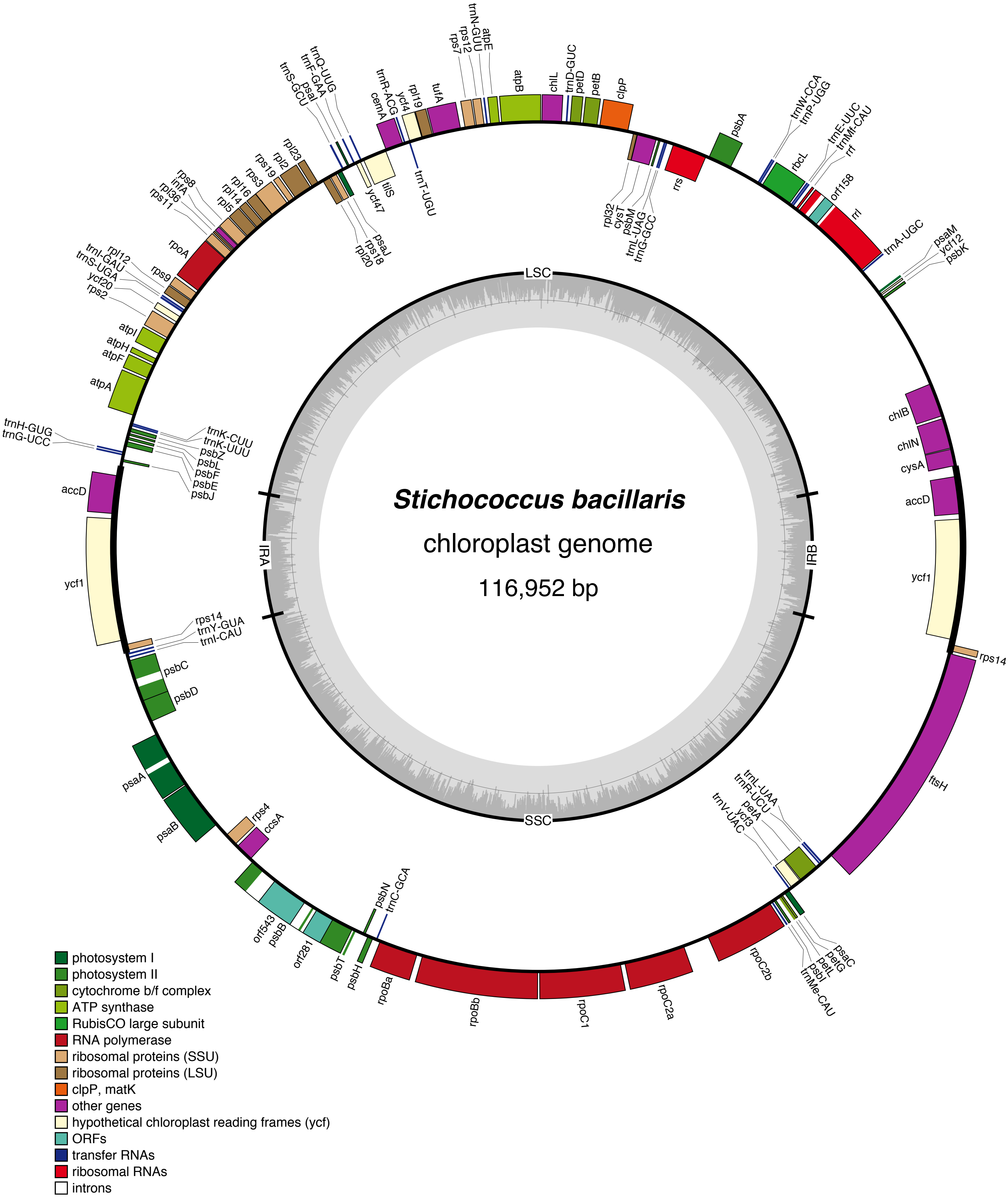
Stichococcus bacillaris
chloroplast genome
116,952 bp
LSC
IRA
IRB
SSC
trnQ-UUG
trnF-GAA
psaI
trnS-GCU
trnR-ACG
cemA
ycf4
rpl19
tufA
rps7
rps12
trnN-GUU
atpE
atpB
chlL
trnD-GUC
petD
petB
clpP
psbA
trnW-CCA
trnP-UGG
rbcL
trnE-UUC
trnMf-CAU
rrf
orf158
rrl
trnA-UGC
psaM
ycf12
psbK
rrs
rpl32
cysT
psbM
trnL-UAG
trnG-GCC
tilS
trnT-UGU
ycf47
psaJ
rps18
rpl20
rpl23
rpl2
rps19
rps3
rpl16
rpl14
rpl5
rps8
infA
rpl36
rps11
rpoA
rps9
rpl12
trnI-GAU
trnS-UGA
ycf20
rps2
atpI
atpH
atpF
atpA
trnH-GUG
trnG-UCC
trnK-CUU
trnK-UUU
psbZ
psbL
psbF
psbE
psbJ
accD
ycf1
rps14
trnY-GUA
trnI-CAU
psbC
psbD
psaA
psaB
rps4
ccsA
orf543
psbB
orf281
psbT
psbH
psbN
trnC-GCA
rpoBa
rpoBb
rpoC1
rpoC2a
rpoC2b
psaC
petG
petL
psbI
trnMe-CAU
trnL-UAA
trnR-UCU
petA
ycf3
trnV-UAC
ftsH
chlB
chlN
cysA
accD
ycf1
rps14
photosystem I
photosystem II
cytochrome b/f complex
ATP synthase
RubisCO large subunit
RNA polymerase
ribosomal proteins (SSU)
ribosomal proteins (LSU)
clpP, matK
other genes
hypothetical chloroplast reading frames (ycf)
ORFs
transfer RNAs
ribosomal RNAs
introns

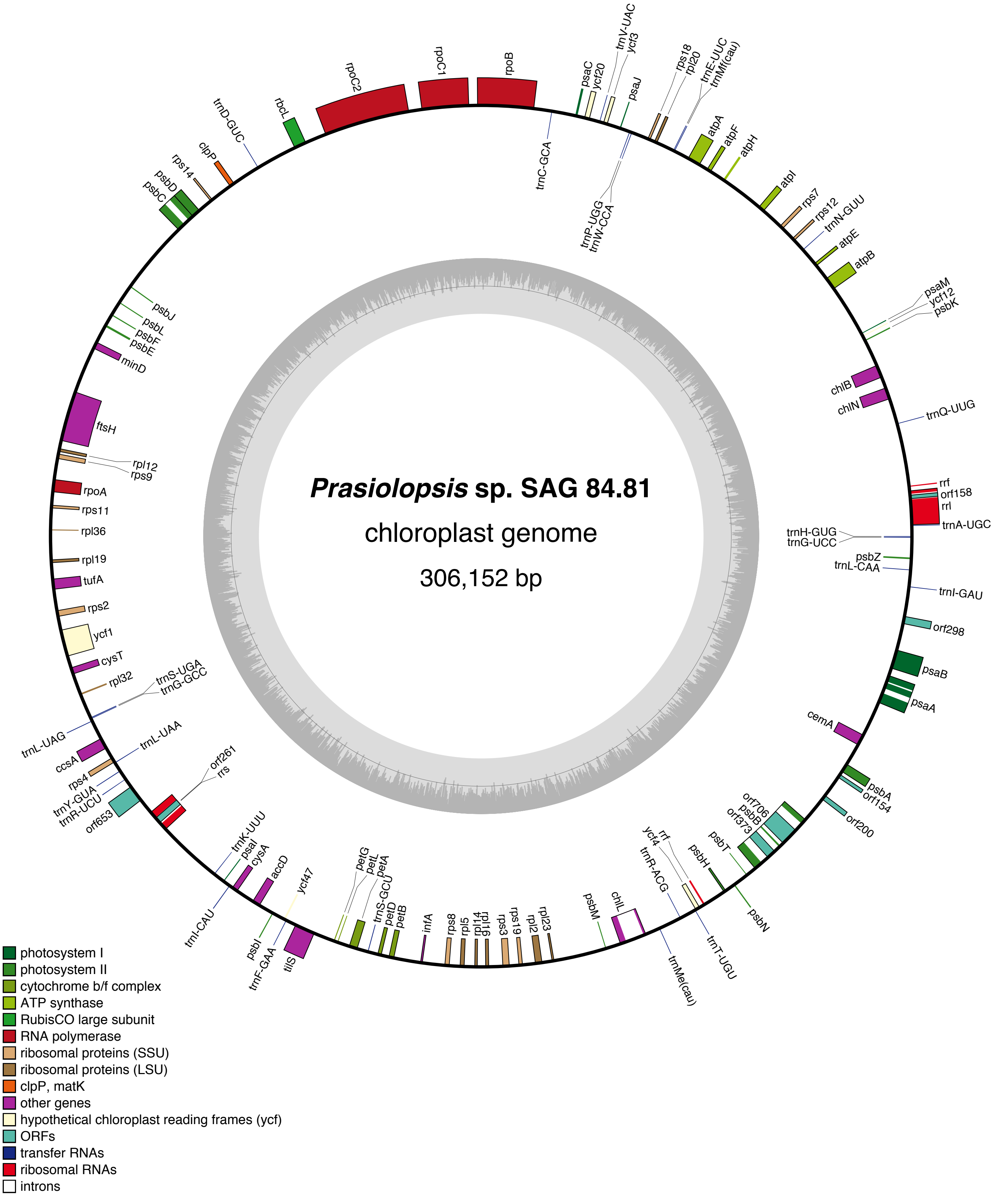

Prasiolopsis sp. SAG 84.81
chloroplast genome
306,152 bp
rpoB
rpoC1
rpoC2
rbcL
trnD-GUC
clpP
rps14
psbD
psbC
trnC-GCA
psaC
ycf20
trnV-UAC
ycf3
psaJ
trnP-UGG
trnW-CCA
rps18
rpl20
trnE-UUC
trnMf(cau)
atpA
atpF
atpH
atpI
rps7
rps12
trnN-GUU
atpE
atpB
psaM
ycf12
psbK
chlB
chlN
trnQ-UUG
rrf
orf158
rrl
trnA-UGC
trnH-GUG
trnG-UCC
psbZ
trnL-CAA
trnI-GAU
orf298
psaB
psaA
cemA
psbA
orf154
orf200
orf706
psbB
orf373
psbT
psbH
psbN
rrf
ycf4
trnR-ACG
trnT-UGU
chlL
trnMe(cau)
psbM
rpl23
rpl2
rps19
rps3
rpl16
rpl14
rpl5
rps8
infA
petB
petD
trnS-GCU
petA
petL
petG
ycf47
tilS
trnF-GAA
psbI
accD
cysA
psaI
trnK-UUU
trnI-CAU
orf261
rrs
orf653
trnR-UCU
trnY-GUA
rps4
ccsA
trnL-UAA
trnL-UAG
trnS-UGA
trnG-GCC
rpl32
cysT
ycf1
rps2
tufA
rpl19
rpl36
rps11
rpoA
rpl12
rps9
ftsH
minD
psbE
psbF
psbL
psbJ
photosystem I
photosystem II
cytochrome b/f complex
ATP synthase
RubisCO large subunit
RNA polymerase
ribosomal proteins (SSU)
ribosomal proteins (LSU)
clpP, matK
other genes
hypothetical chloroplast reading frames (ycf)
ORFs
transfer RNAs
ribosomal RNAs
introns

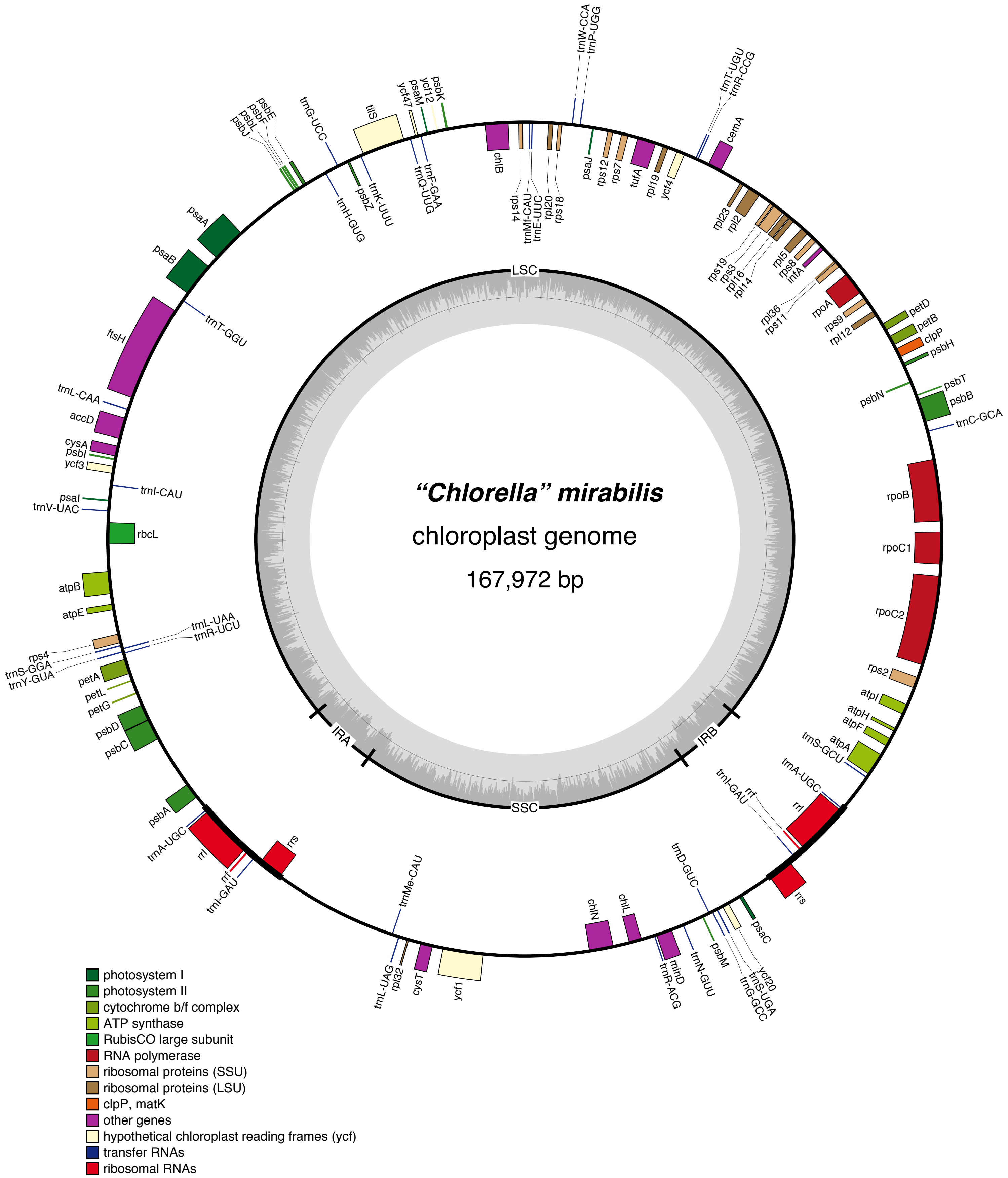

“Chlorella” mirabilis
chloroplast genome
167,972 bp
LSC
IRA
IRB
SSC
trnW-CCA
trnP-UGG
trnT-UGU
trnR-CCG
cemA
psbK
ycf12
psaM
ycf47
tilS
trnG-UCC
psbE
psbF
psbL
psbJ
trnH-GUG
psbZ
trnK-UUU
trnF-GAA
trnQ-UUG
chlB
rps14
trnMf-CAU
trnE-UUC
rpl20
rps18
psaJ
rps12
rps7
tufA
rpl19
ycf4
rpl23
rpl2
rps19
rps3
rpl16
rpl14
rpl5
rps8
infA
rpl36
rps11
rpoA
rps9
rpl12
petD
petB
clpP
psbH
psbN
psbT
psbB
trnC-GCA
psaA
psaB
trnT-GGU
ftsH
trnL-CAA
accD
cysA
psbI
ycf3
trnI-CAU
psaI
trnV-UAC
rbcL
atpB
atpE
trnL-UAA
trnR-UCU
rps4
trnS-GGA
trnY-GUA
petA
petL
petG
psbD
psbC
psbA
trnA-UGC
rrl
rrf
trnI-GAU
rrs
rpoB
rpoC1
rpoC2
rps2
atpI
atpH
atpF
atpA
trnS-GCU
trnMe-CAU
trnL-UAG
rpl32
cysT
ycf1
chlN
chlL
minD
trnR-ACG
trnD-GUC
trnN-GUU
psbM
psaC
ycf20
trnS-UGA
trnG-GCC
photosystem I
photosystem II
cytochrome b/f complex
ATP synthase
RubisCO large subunit
RNA polymerase
ribosomal proteins (SSU)
ribosomal proteins (LSU)
clpP, matK
other genes
hypothetical chloroplast reading frames (ycf)
transfer RNAs
ribosomal RNAs

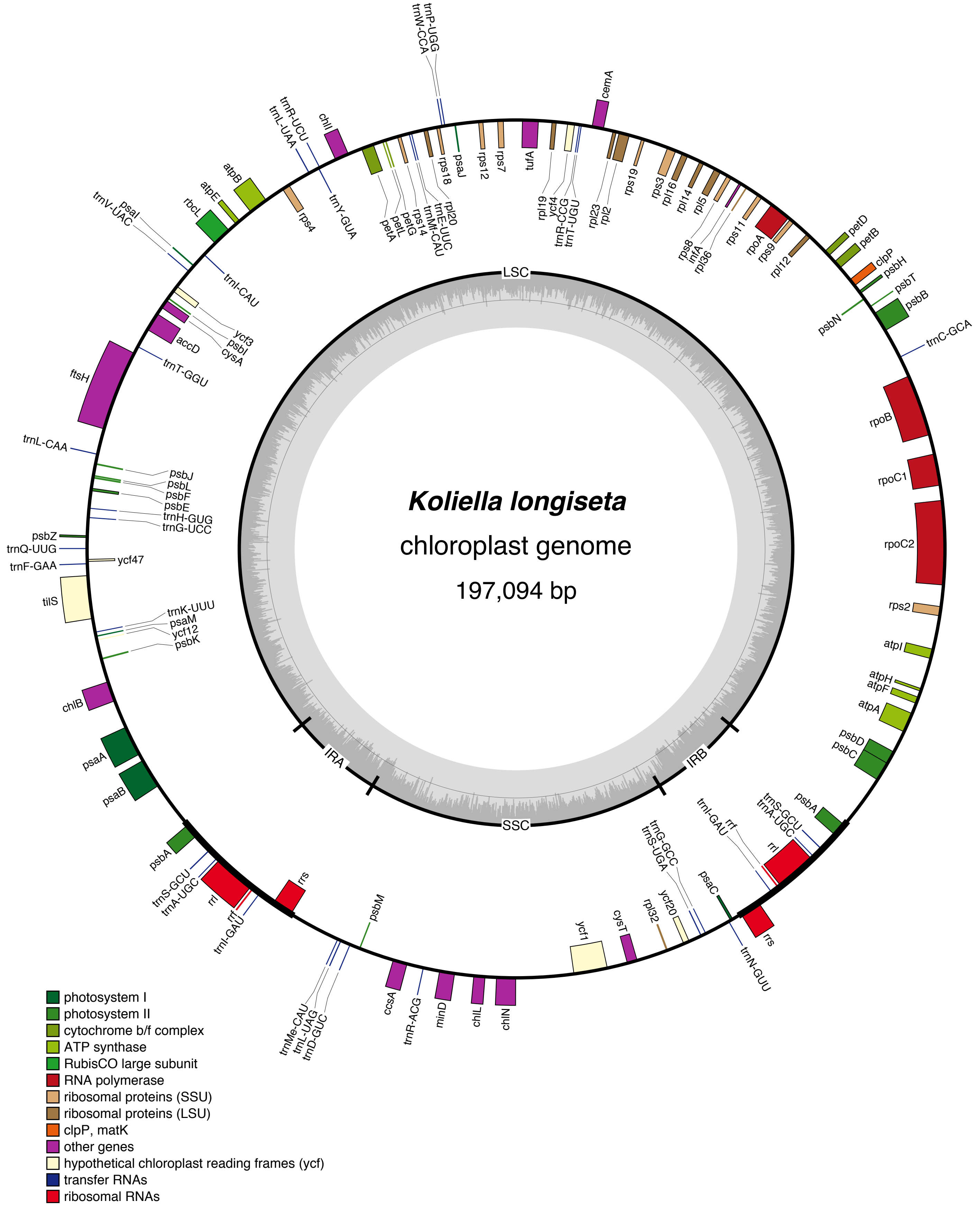

Koliella longiseta
chloroplast genome
197,094 bp
LSC
IRA
IRB
SSC
trnP-UGG
trnW-CCA
cemA
trnR-UCU
trnL-UAA
chlI
atpB
atpE
rbcL
trnV-UAC
psaI
rps4
trnY-GUA
petA
petL
petG
rps14
trnMf-CAU
trnE-UUC
rpl20
rps18
psaJ
rps12
rps7
tufA
rpl19
ycf4
trnR-CCG
trnT-UGU
rpl23
rpl2
rps19
rps3
rpl16
rpl14
rpl5
rps8
infA
rpl36
rps11
rpoA
rps9
rpl12
petD
petB
clpP
psbH
psbT
psbN
psbB
trnC-GCA
trnI-CAU
ycf3
psbI
cysA
accD
trnT-GGU
ftsH
trnL-CAA
psbJ
psbL
psbF
psbE
trnH-GUG
trnG-UCC
psbZ
trnQ-UUG
trnF-GAA
ycf47
tilS
trnK-UUU
psaM
ycf12
psbK
chlB
psaA
psaB
psbA
trnS-GCU
trnA-UGC
rrl
rrf
trnI-GAU
rrs
psbM
trnMe-CAU
trnL-UAG
trnD-GUC
ccsA
trnR-ACG
minD
chlL
chlN
ycf1
cysT
rpl32
ycf20
trnG-GCC
trnS-UGA
psaC
trnN-GUU
rpoB
rpoC1
rpoC2
rps2
atpI
atpH
atpF
atpA
psbD
psbC
photosystem I
photosystem II
cytochrome b/f complex
ATP synthase
RubisCO large subunit
RNA polymerase
ribosomal proteins (SSU)
ribosomal proteins (LSU)
clpP, matK
other genes
hypothetical chloroplast reading frames (ycf)
transfer RNAs
ribosomal RNAs

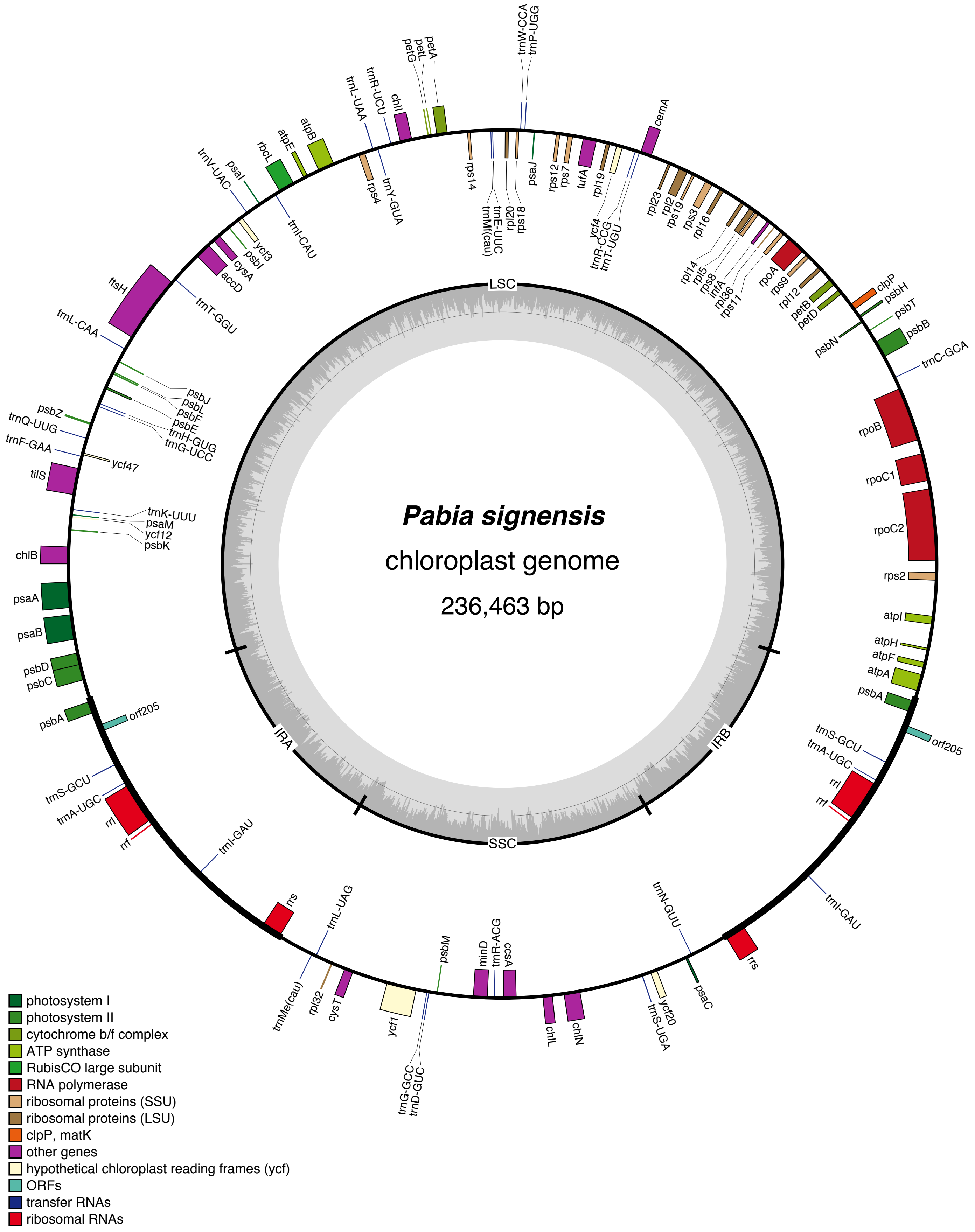

Pabia signensis
chloroplast genome
236,463 bp
LSC
IRA
IRB
SSC
trnW-CCA
trnP-UGG
petA
petL
petG
chlI
trnR-UCU
trnL-UAA
atpB
atpE
rbcL
psaI
trnV-UAC
cemA
rps14
trnMf(cau)
trnE-UUC
rpl20
rps18
psaJ
rps12
rps7
tufA
rpl19
ycf4
trnR-CCG
trnT-UGU
rps4
trnY-GUA
trnI-CAU
ycf3
psbI
cysA
accD
ftsH
trnT-GGU
trnL-CAA
rpl23
rpl2
rps19
rps3
rpl16
rpl14
rpl5
rps8
infA
rpl36
rps11
rpoA
rps9
rpl12
petB
petD
clpP
psbH
psbT
psbN
psbB
trnC-GCA
rpoB
rpoC1
rpoC2
rps2
atpI
atpH
atpF
atpA
psbA
orf205
trnS-GCU
trnA-UGC
rrl
rrf
trnI-GAU
rrs
psbJ
psbL
psbF
psbE
trnH-GUG
trnG-UCC
psbZ
trnQ-UUG
trnF-GAA
ycf47
tilS
trnK-UUU
psaM
ycf12
psbK
chlB
psaA
psaB
psbD
psbC
psbA
orf205
trnS-GCU
trnA-UGC
rrl
rrf
trnI-GAU
rrs
trnL-UAG
trnMe(cau)
rpl32
cysT
ycf1
trnG-GCC
trnD-GUC
psbM
minD
trnR-ACG
ccsA
chlL
chlN
trnN-GUU
psaC
ycf20
trnS-UGA
trnI-GAU
rrs
photosystem I
photosystem II
cytochrome b/f complex
ATP synthase
RubisCO large subunit
RNA polymerase
ribosomal proteins (SSU)
ribosomal proteins (LSU)
clpP, matK
other genes
hypothetical chloroplast reading frames (ycf)
ORFs
transfer RNAs
ribosomal RNAs

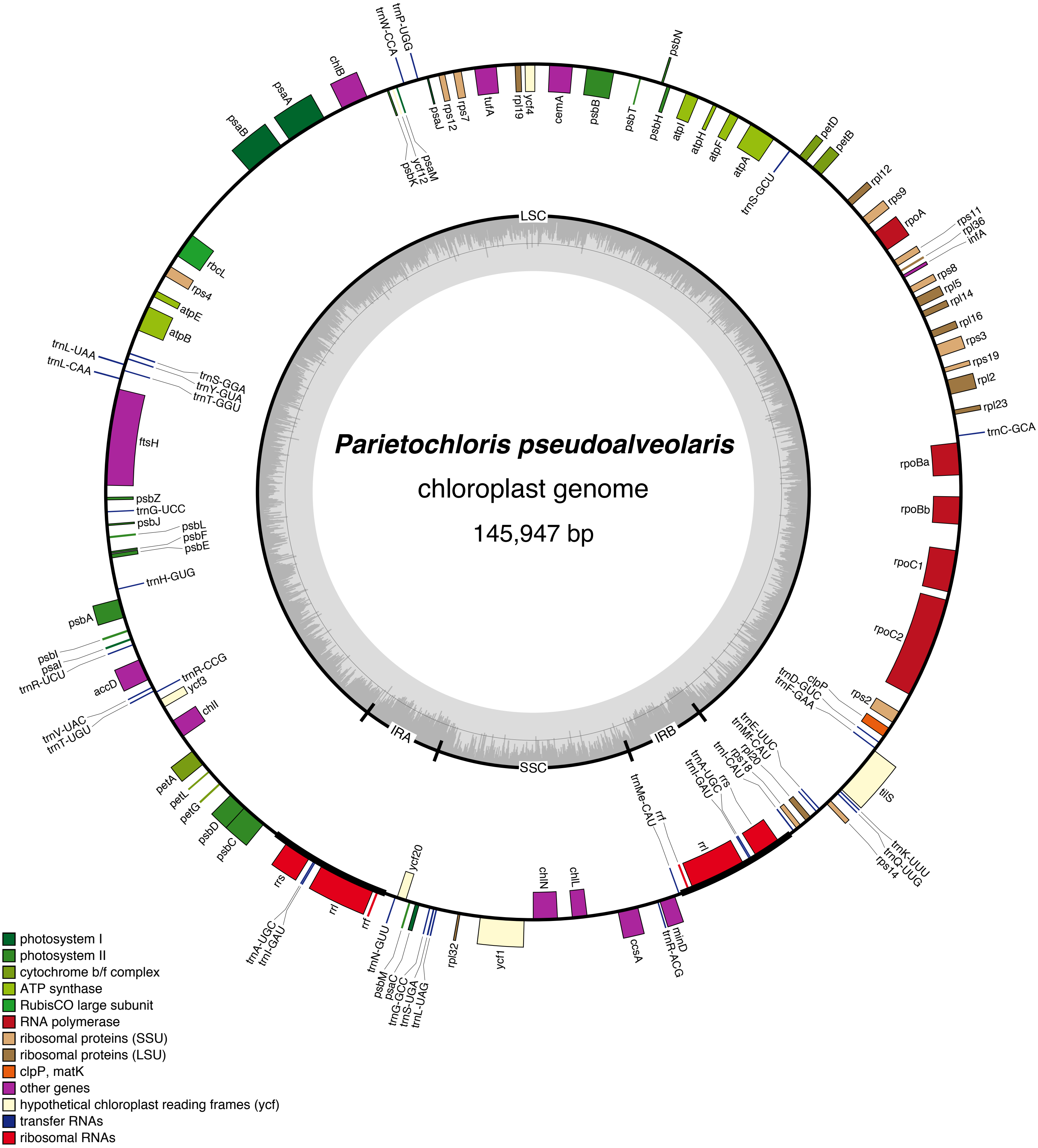

Parietochloris pseudoalveolaris
chloroplast genome
145,947 bp
LSC
IRA
SSC
IRB
trnP-UGG
trnW-CCA
psbN
chlB
psaA
psaB
psaM
ycf12
psbK
psaJ
rps12
rps7
tufA
rpl19
ycf4
cemA
psbB
psbT
psbH
atpI
atpH
atpF
atpA
trnS-GCU
petD
petB
rpl12
rps9
rpoA
rps11
rpl36
infA
rps8
rpl5
rpl14
rpl16
rps3
rps19
rpl2
rpl23
trnC-GCA
rpoBa
rpoBb
rpoC1
rpoC2
rps2
clpP
trnD-GUC
trnF-GAA
tilS
trnK-UUU
trnQ-UUG
rps14
trnE-UUC
trnMf-CAU
rpl20
rps18
trnI-CAU
rrs
trnA-UGC
trnI-GAU
rrl
rrf
trnMe-CAU
minD
trnR-ACG
ccsA
chlL
chlN
ycf1
rpl32
trnL-UAG
trnS-UGA
trnG-GCC
psaC
psbM
trnN-GUU
rrf
rrl
ycf20
trnI-GAU
trnA-UGC
rrs
psbC
psbD
petG
petL
petA
chlI
ycf3
trnR-CCG
trnT-UGU
trnV-UAC
accD
trnR-UCU
psaI
psbI
psbA
trnH-GUG
psbE
psbF
psbL
psbJ
trnG-UCC
psbZ
ftsH
trnT-GGU
trnY-GUA
trnS-GGA
trnL-CAA
trnL-UAA
atpB
atpE
rps4
rbcL
photosystem I
photosystem II
cytochrome b/f complex
ATP synthase
RubisCO large subunit
RNA polymerase
ribosomal proteins (SSU)
ribosomal proteins (LSU)
clpP, matK
other genes
hypothetical chloroplast reading frames (ycf)
transfer RNAs
ribosomal RNAs

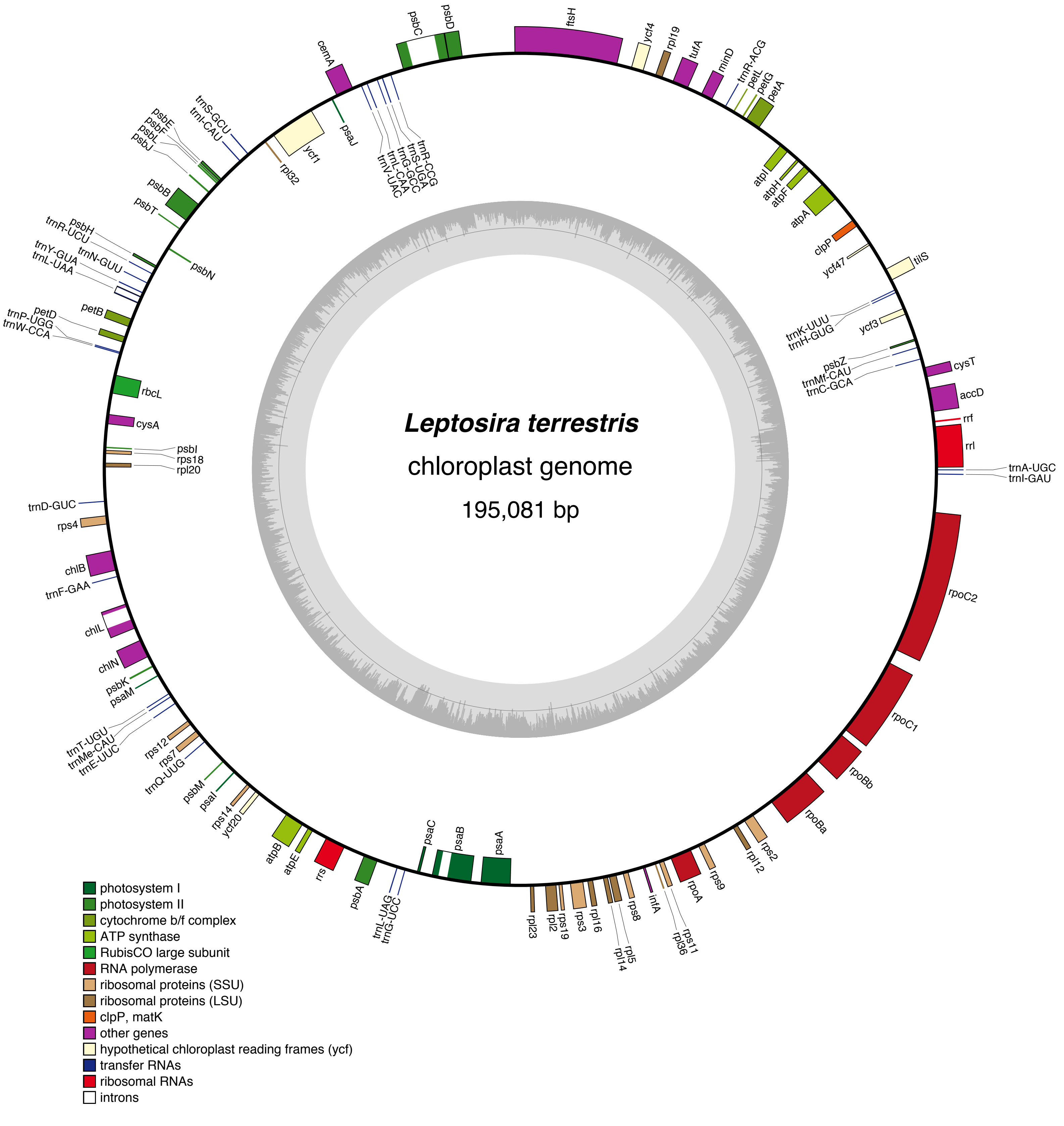

Leptosira terrestris
chloroplast genome
195,081 bp
ftsH
ycf4
rpl19
tufA
minD
trnR-ACG
petL
petG
petA
atpI
atpH
atpF
atpA
clpP
ycf47
tilS
trnK-UUU
trnH-GUG
ycf3
psbZ
trnMf-CAU
trnC-GCA
cysT
accD
rrf
rrl
trnA-UGC
trnI-GAU
rpoC2
rpoC1
rpoBb
rpoBa
rps2
rpl12
rps9
rpoA
rps11
rpl36
infA
rps8
rpl5
rpl14
rpl16
rps3
rps19
rpl2
rpl23
psaA
psaB
psaC
trnL-UAG
trnG-UCC
psbA
rrs
atpE
atpB
ycf20
rps14
psaI
psbM
trnQ-UUG
rps7
rps12
trnE-UUC
trnMe-CAU
trnT-UGU
psaM
psbK
chlN
chlL
trnF-GAA
chlB
rps4
trnD-GUC
rpl20
rps18
psbI
cysA
rbcL
trnW-CCA
trnP-UGG
petD
petB
trnL-UAA
trnY-GUA
trnN-GUU
trnR-UCU
psbH
psbN
psbT
psbB
psbJ
psbL
psbF
psbE
trnI-CAU
trnS-GCU
rpl32
ycf1
cemA
psaJ
trnV-UAC
trnL-CAA
trnG-GCC
trnS-UGA
trnR-CCG
psbC
psbD
photosystem I
photosystem II
cytochrome b/f complex
ATP synthase
RubisCO large subunit
RNA polymerase
ribosomal proteins (SSU)
ribosomal proteins (LSU)
clpP, matK
other genes
hypothetical chloroplast reading frames (ycf)
transfer RNAs
ribosomal RNAs
introns

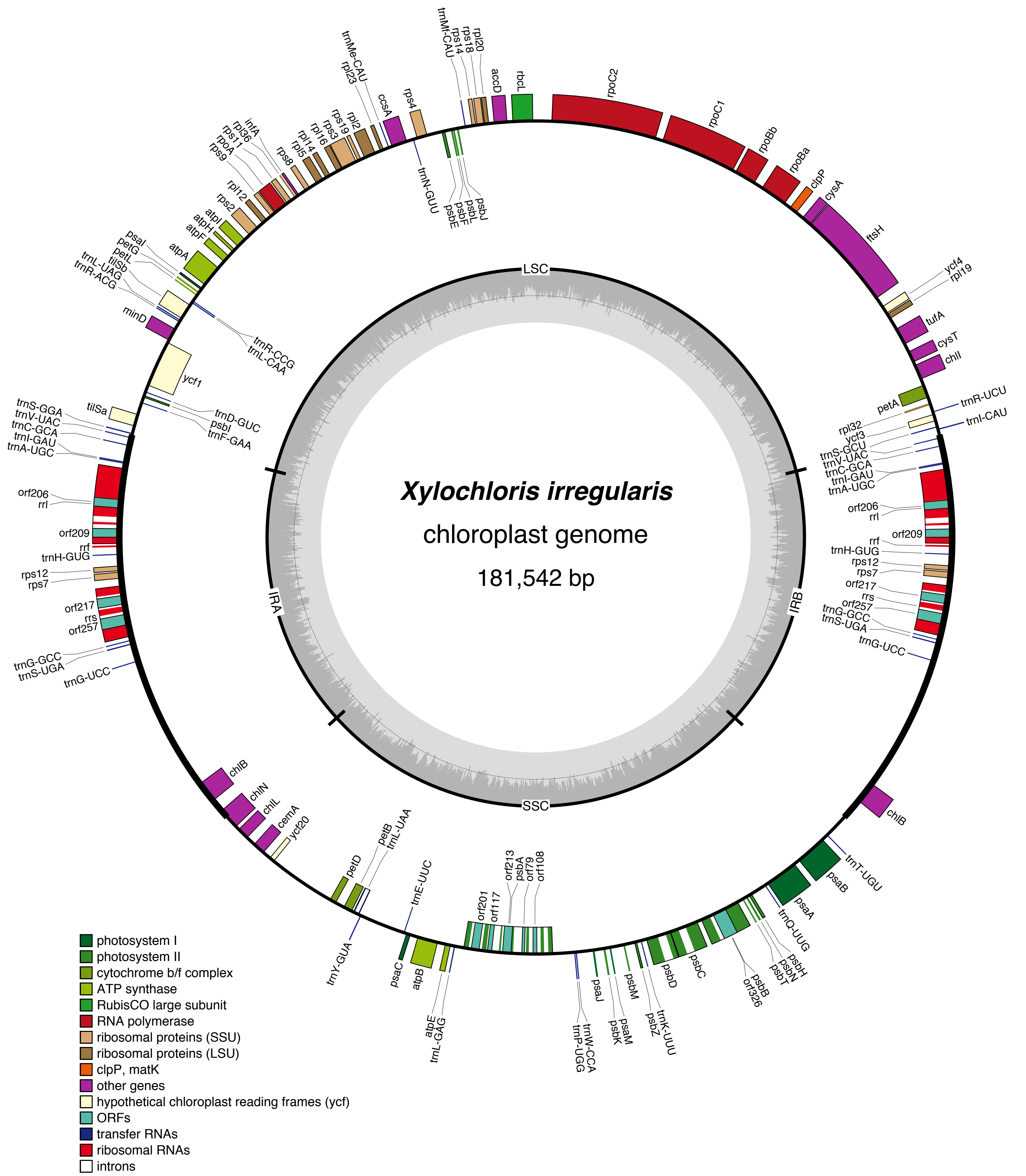

Xylochloris irregularis
chloroplast genome
181,542 bp
LSC
IRA
IRB
SSC
rpoC2
rpoC1
rpoBb
rpoBa
clpP
cysA
ftsH
ycf4
rpl19
tufA
cysT
chlI
petA
trnR-UCU
trnI-CAU
rpl32
ycf3
trnS-GCU
trnV-UAC
trnC-GCA
trnI-GAU
trnA-UGC
orf206
rrl
orf209
rrf
trnH-GUG
rps12
rps7
orf217
rrs
orf257
trnG-GCC
trnS-UGA
trnG-UCC
chlB
trnT-UGU
psaB
psaA
trnQ-UUG
psbH
psbN
psbT
psbB
orf326
psbC
psbD
trnK-UUU
psbZ
psaM
psbK
psbM
psaJ
trnW-CCA
trnP-UGG
orf201
orf117
orf213
psbA
orf79
orf108
trnE-UUC
psaC
atpB
atpE
trnL-GAG
trnY-GUA
petD
petB
trnL-UAA
ycf20
cemA
chlL
chlN
tilSa
trnS-GGA
ycf1
trnD-GUC
psbI
trnF-GAA
minD
trnR-CCG
trnL-CAA
trnL-UAG
trnR-ACG
tilSb
petL
petG
psaI
atpA
atpF
atpH
atpI
rps2
rpl12
rps9
rpoA
rps11
rpl36
infA
rps8
rpl5
rpl14
rpl16
rps3
rps19
rpl2
rpl23
trnMe-CAU
ccsA
rps4
trnN-GUU
trnMf-CAU
rps14
rps18
rpl20
accD
rbcL
psbE
psbF
psbL
psbJ
photosystem I
photosystem II
cytochrome b/f complex
ATP synthase
RubisCO large subunit
RNA polymerase
ribosomal proteins (SSU)
ribosomal proteins (LSU)
clpP, matK
other genes
hypothetical chloroplast reading frames (ycf)
ORFs
transfer RNAs
ribosomal RNAs
introns

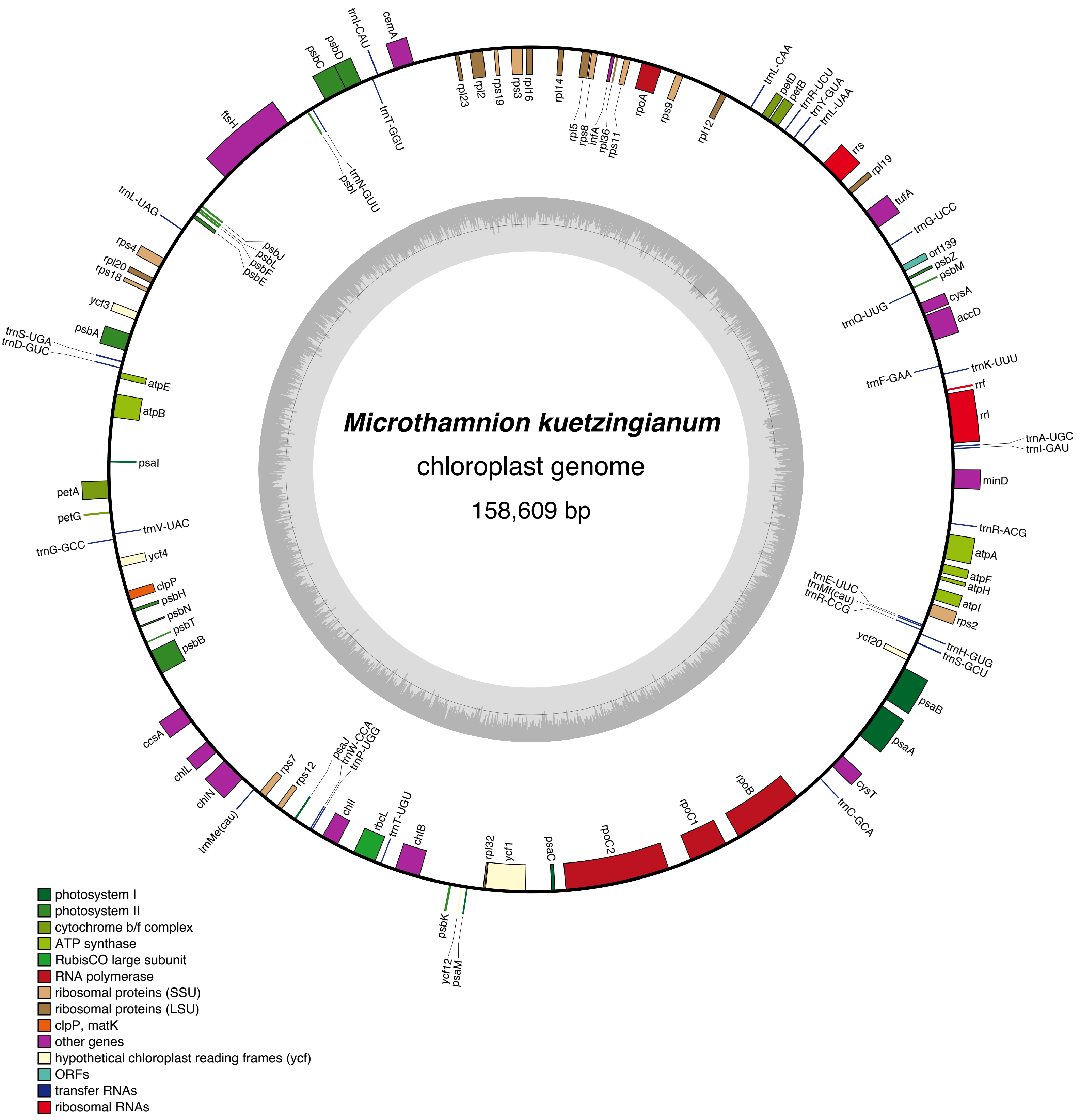
Microthamnion kuetzingianum
chloroplast genome
158,609 bp
cemA
trnI-CAU
psbD
psbC
trnT-GGU
ftsH
trnN-GUU
psbI
rpl23
rpl2
rps19
rps3
rpl16
rpl14
rpl5
rps8
infA
rpl36
rps11
rpoA
rps9
rpl12
trnL-CAA
petD
petB
trnR-UCU
trnY-GUA
trnL-UAA
rrs
rpl19
tufA
trnG-UCC
orf139
psbZ
psbM
cysA
trnQ-UUG
accD
trnK-UUU
trnF-GAA
rrf
rrl
trnA-UGC
trnI-GAU
minD
trnR-ACG
atpA
atpF
atpH
atpI
trnE-UUC
trnMf(cau)
trnR-CCG
rps2
ycf20
trnH-GUG
trnS-GCU
psaB
psaA
cysT
trnC-GCA
rpoB
rpoC1
rpoC2
psaC
ycf1
rpl32
psbK
ycf12
psaM
chlB
trnT-UGU
rbcL
chlI
psaJ
trnW-CCA
trnP-UGG
rps12
rps7
trnMe(cau)
chlN
chlL
ccsA
psbB
psbT
psbN
psbH
clpP
ycf4
trnV-UAC
trnG-GCC
petG
petA
psaI
atpB
atpE
trnS-UGA
trnD-GUC
psbA
ycf3
rps18
rpl20
rps4
trnL-UAG
psbJ
psbL
psbF
psbE
photosystem I
photosystem II
cytochrome b/f complex
ATP synthase
RubisCO large subunit
RNA polymerase
ribosomal proteins (SSU)
ribosomal proteins (LSU)
clpP, matK
other genes
hypothetical chloroplast reading frames (ycf)
ORFs
transfer RNAs
ribosomal RNAs

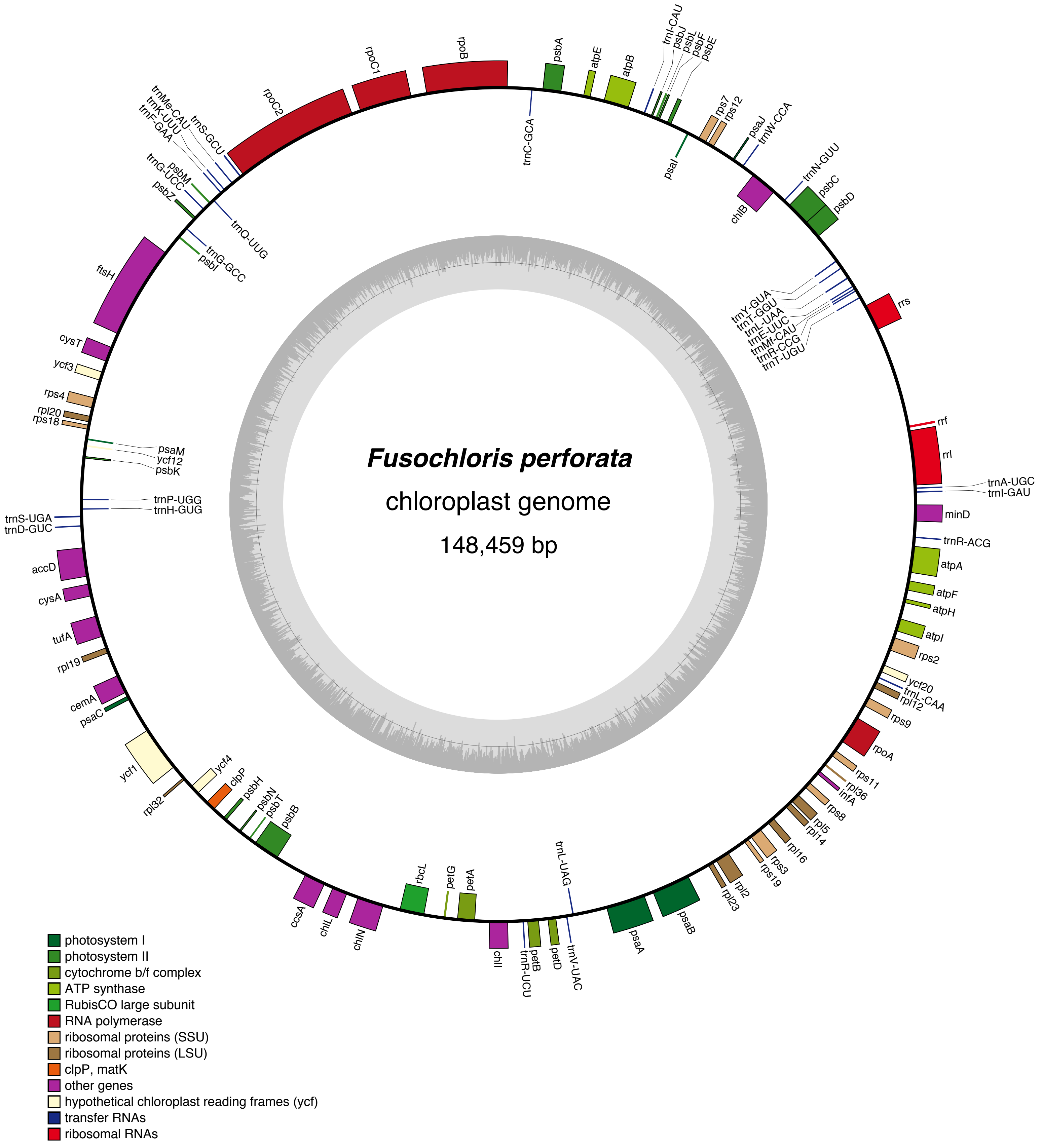
Fusochloris perforata
chloroplast genome
148,459 bp
rpoB
rpoC1
rpoC2
psbA
atpE
atpB
trnI-CAU
psbJ
psbL
psbF
psbE
trnC-GCA
psaI
rps7
rps12
psaJ
trnW-CCA
trnN-GUU
psbC
psbD
chlB
trnMe-CAU
trnK-UUU
trnF-GAA
trnS-GCU
psbM
trnG-UCC
psbZ
trnQ-UUG
trnG-GCC
psbI
ftsH
cysT
ycf3
rps4
rpl20
rps18
psaM
ycf12
psbK
trnP-UGG
trnH-GUG
trnS-UGA
trnD-GUC
accD
cysA
tufA
rpl19
cemA
psaC
ycf1
rpl32
ycf4
clpP
psbH
psbN
psbT
psbB
ccsA
chlL
chlN
rbcL
petG
petA
chlI
trnR-UCU
petB
petD
trnV-UAC
trnL-UAG
psaA
psaB
rpl23
rpl2
rps19
rps3
rpl16
rpl14
rpl5
rps8
infA
rpl36
rps11
rpoA
rps9
rpl12
trnL-CAA
ycf20
rps2
atpI
atpH
atpF
atpA
trnR-ACG
minD
trnA-UGC
trnI-GAU
rrl
rrf
rrs
trnY-GUA
trnT-GGU
trnL-UAA
trnE-UUC
trnMf-CAU
trnR-CCG
trnT-UGU
photosystem I
photosystem II
cytochrome b/f complex
ATP synthase
RubisCO large subunit
RNA polymerase
ribosomal proteins (SSU)
ribosomal proteins (LSU)
clpP, matK
other genes
hypothetical chloroplast reading frames (ycf)
transfer RNAs
ribosomal RNAs

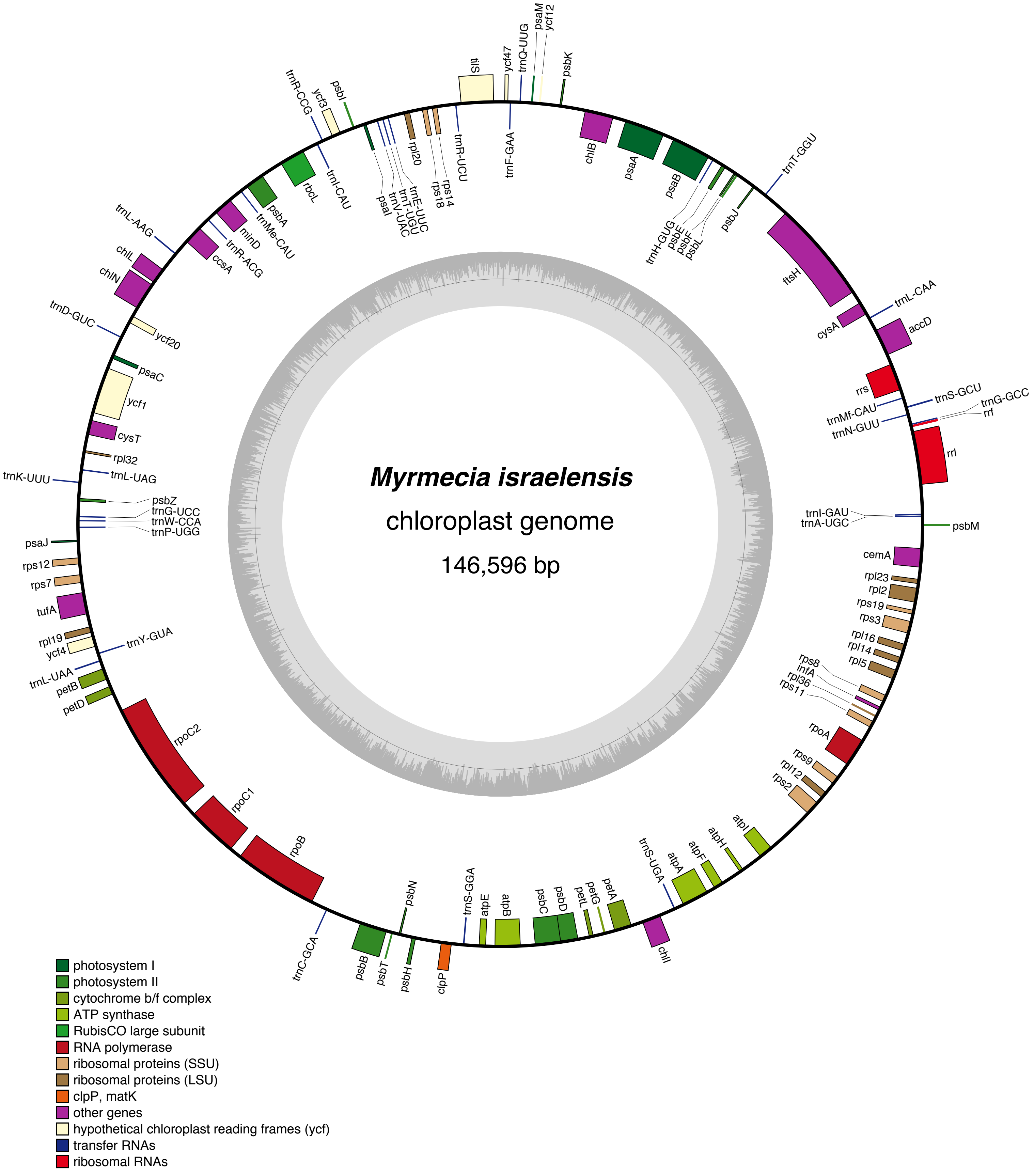
Myrmecia israelensis
chloroplast genome
146,596 bp
tilS
ycf47
trnQ-UUG
psaM
ycf12
psbK
trnF-GAA
chlB
psaA
psaB
trnT-GGU
trnH-GUG
psbE
psbF
psbL
psbJ
ftsH
trnL-CAA
cysA
accD
rrs
trnS-GCU
trnMf-CAU
trnN-GUU
trnG-GCC
rrf
rrl
trnI-GAU
trnA-UGC
psbM
cemA
rpl23
rpl2
rps19
rps3
rpl16
rpl14
rpl5
rps8
infA
rpl36
rps11
rpoA
rps9
rpl12
rps2
atpI
atpH
atpF
atpA
trnS-UGA
chlI
petA
petG
petL
psbD
psbC
atpB
atpE
trnS-GGA
clpP
psbN
psbH
psbT
psbB
trnC-GCA
rpoB
rpoC1
rpoC2
petD
petB
trnL-UAA
trnY-GUA
ycf4
rpl19
tufA
rps7
rps12
psaJ
trnK-UUU
trnL-UAG
psbZ
trnG-UCC
trnW-CCA
trnP-UGG
rpl32
cysT
ycf1
psaC
ycf20
trnD-GUC
chlN
chlL
trnL-AAG
ccsA
trnR-ACG
minD
trnMe-CAU
psbA
rbcL
trnI-CAU
trnR-CCG
ycf3
psbI
psaI
trnV-UAC
trnT-UGU
trnE-UUC
rpl20
rps18
rps14
trnR-UCU
photosystem I
photosystem II
cytochrome b/f complex
ATP synthase
RubisCO large subunit
RNA polymerase
ribosomal proteins (SSU)
ribosomal proteins (LSU)
clpP, matK
other genes
hypothetical chloroplast reading frames (ycf)
transfer RNAs
ribosomal RNAs

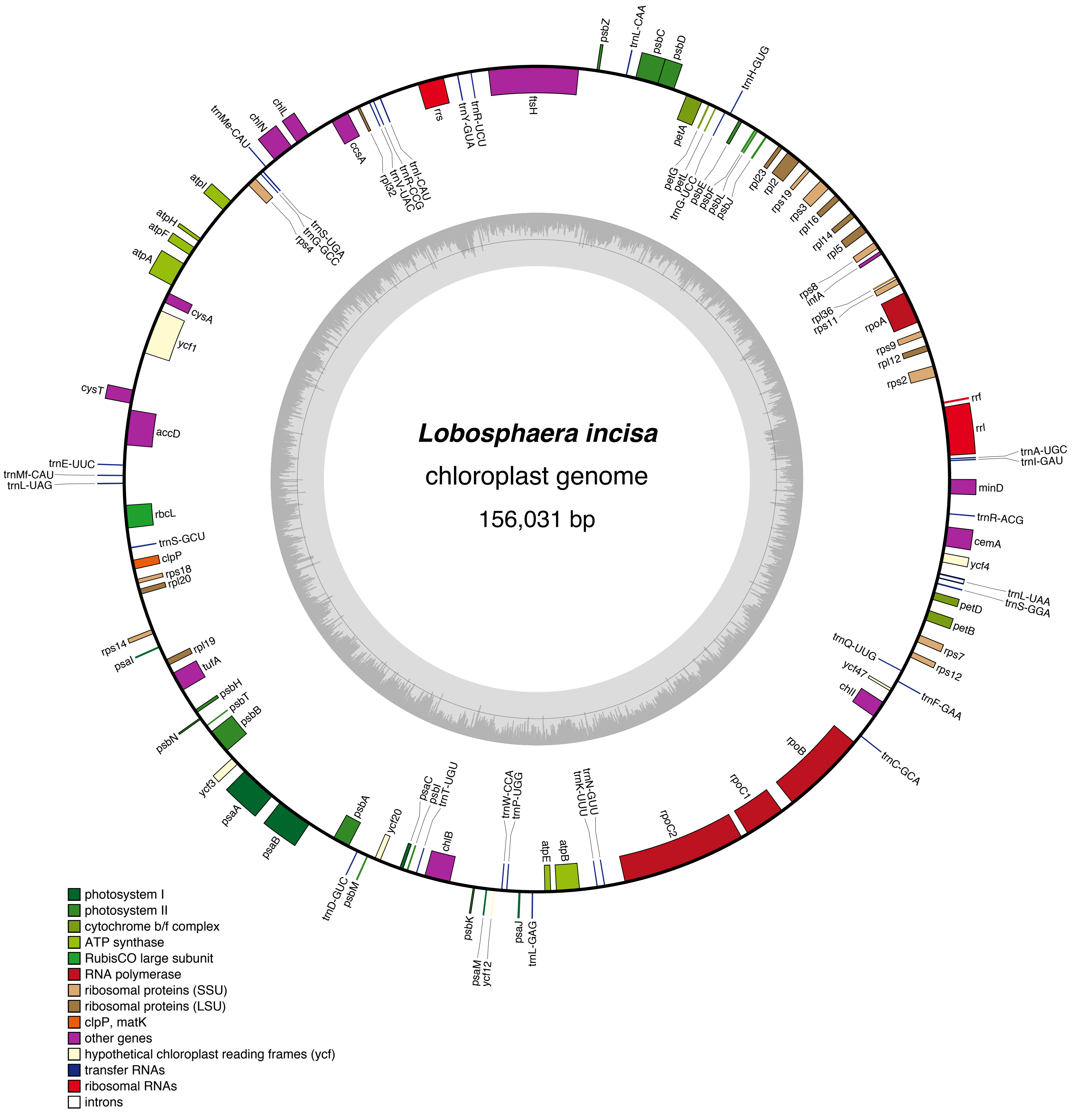

Lobosphaera incisa
chloroplast genome
156,031 bp
psbZ
trnL-CAA
psbC
psbD
trnH-GUG
ftsH
trnR-UCU
trnY-GUA
rrs
trnI-CAU
trnR-CCG
trnV-UAC
rpl32
ccsA
chlL
chlN
trnMe-CAU
trnS-UGA
trnG-GCC
rps4
atpI
atpH
atpF
atpA
cysA
ycf1
cysT
accD
trnE-UUC
trnMf-CAU
trnL-UAG
rbcL
trnS-GCU
clpP
rps18
rpl20
rps14
psaI
rpl19
tufA
psbH
psbT
psbB
psbN
ycf3
psaA
psaB
psbA
trnD-GUC
psbM
ycf20
psaC
psbI
trnT-UGU
chlB
trnW-CCA
trnP-UGG
psbK
psaM
ycf12
psaJ
trnL-GAG
atpE
atpB
trnN-GUU
trnK-UUU
rpoC2
rpoC1
rpoB
trnC-GCA
chlI
ycf47
trnQ-UUG
trnF-GAA
rps12
rps7
petB
petD
trnL-UAA
trnS-GGA
ycf4
cemA
trnR-ACG
minD
trnA-UGC
trnI-GAU
rrl
rrf
rps2
rpl12
rps9
rpoA
rpl36
rps11
rps8
infA
rpl5
rpl14
rpl16
rps3
rps19
rpl2
rpl23
petA
petG
petL
trnG-UCC
psbE
psbF
psbL
psbJ
photosystem I
photosystem II
cytochrome b/f complex
ATP synthase
RubisCO large subunit
RNA polymerase
ribosomal proteins (SSU)
ribosomal proteins (LSU)
clpP, matK
other genes
hypothetical chloroplast reading frames (ycf)
transfer RNAs
ribosomal RNAs
introns

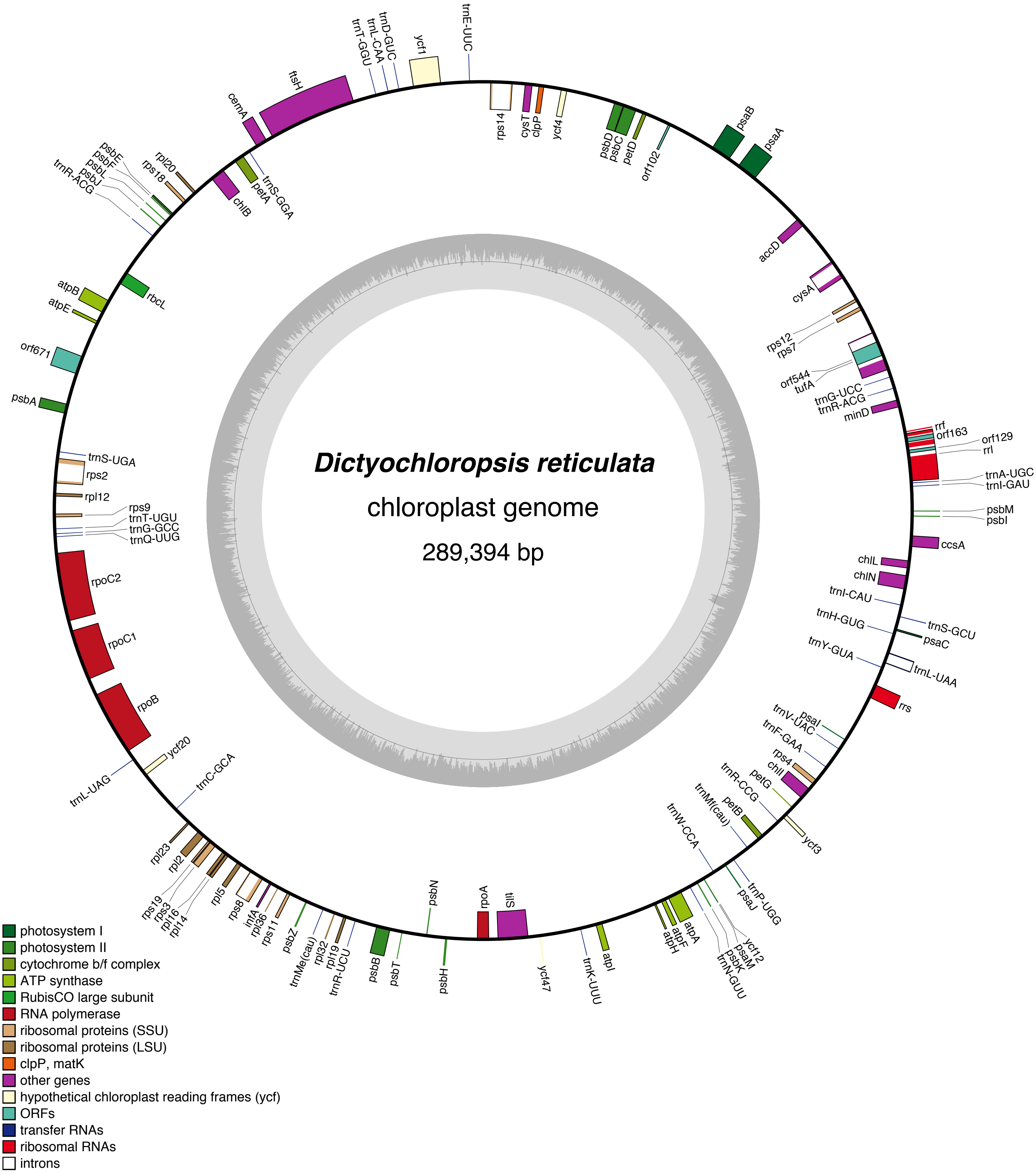

Dictyochloropsis reticulata
chloroplast genome
289,394 bp
trnE-UUC
rps14
cysT
clpP
ycf4
psbD
psbC
petD
orf102
psaB
psaA
accD
cysA
rps12
rps7
orf544
tufA
trnG-UCC
trnR-ACG
minD
rrf
orf163
orf129
rrl
trnA-UGC
trnI-GAU
psbM
psbI
ccsA
chlL
chlN
trnI-CAU
trnS-GCU
trnH-GUG
psaC
trnY-GUA
trnL-UAA
rrs
psaI
trnV-UAC
trnF-GAA
rps4
chlI
petG
trnR-CCG
ycf3
petB
trnMf(cau)
trnW-CCA
trnP-UGG
psaJ
ycf12
psaM
psbK
trnN-GUU
atpA
atpF
atpH
atpI
trnK-UUU
ycf47
tilS
rpoA
psbN
psbH
psbT
psbB
trnR-UCU
rpl19
rpl32
trnMe(cau)
psbZ
rps11
rpl36
infA
rps8
rpl5
rpl14
rpl16
rps3
rps19
rpl2
rpl23
trnC-GCA
trnL-UAG
ycf20
rpoB
rpoC1
rpoC2
trnQ-UUG
trnG-GCC
trnT-UGU
rps9
rpl12
rps2
trnS-UGA
psbA
orf671
atpE
atpB
rbcL
trnR-ACG
psbJ
psbL
psbF
psbE
rps18
rpl20
chlB
petA
trnS-GGA
cemA
ftsH
trnT-GGU
trnL-CAA
trnD-GUC
ycf1
photosystem I
photosystem II
cytochrome b/f complex
ATP synthase
RubisCO large subunit
RNA polymerase
ribosomal proteins (SSU)
ribosomal proteins (LSU)
clpP, matK
other genes
hypothetical chloroplast reading frames (ycf)
ORFs
transfer RNAs
ribosomal RNAs
introns

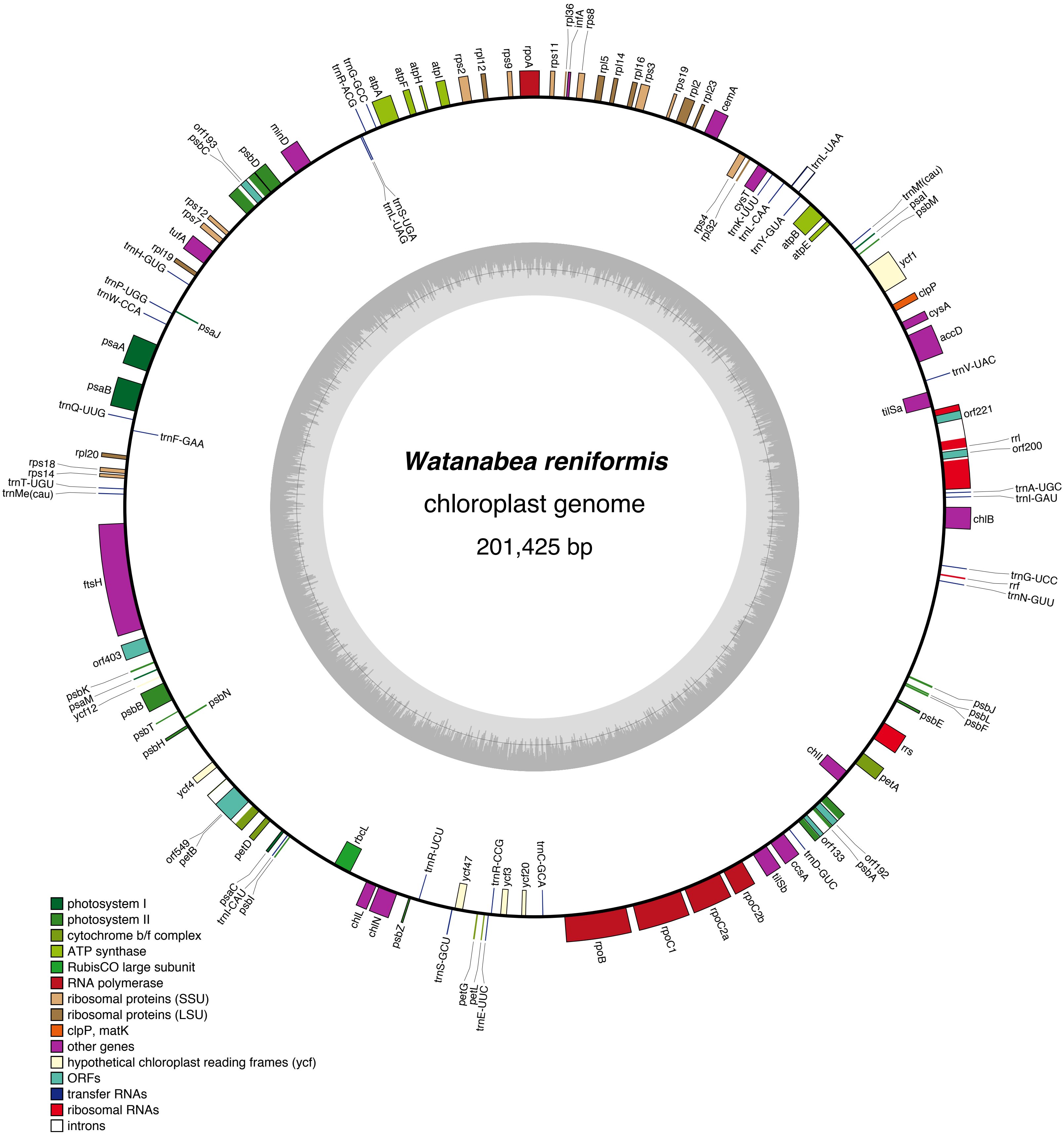
Watanabea reniformis
chloroplast genome
201,425 bp
rpl36
infA
rps8
rps11
rpoA
rps9
rpl12
rps2
atpI
atpH
atpF
atpA
trnG-GCC
trnR-ACG
trnS-UGA
trnL-UAG
minD
psbD
orf193
psbC
rps12
rps7
tufA
rpl19
trnH-GUG
trnP-UGG
trnW-CCA
psaJ
psaA
psaB
trnQ-UUG
trnF-GAA
rpl20
rps18
rps14
trnT-UGU
trnMe(cau)
ftsH
orf403
psbK
psaM
ycf12
psbB
psbN
psbT
psbH
ycf4
orf549
petB
petD
psaC
trnI-CAU
psbI
rbcL
chlL
chlN
psbZ
trnR-UCU
trnS-GCU
ycf47
petG
petL
trnE-UUC
trnR-CCG
ycf3
ycf20
trnC-GCA
rpoB
rpoC1
rpoC2a
rpoC2b
tilSb
ccsA
trnD-GUC
orf133
psbA
orf192
chlI
petA
rrs
psbE
psbJ
psbL
psbF
trnG-UCC
rrf
trnN-GUU
chlB
trnA-UGC
trnI-GAU
rrl
orf200
orf221
tilSa
trnV-UAC
accD
cysA
clpP
ycf1
trnMf(cau)
psaI
psbM
atpB
atpE
trnY-GUA
trnL-CAA
trnK-UUU
cysT
rpl32
rps4
trnL-UAA
cemA
rpl23
rpl2
rps19
rps3
rpl16
rpl14
rpl5
photosystem I
photosystem II
cytochrome b/f complex
ATP synthase
RubisCO large subunit
RNA polymerase
ribosomal proteins (SSU)
ribosomal proteins (LSU)
clpP, matK
other genes
hypothetical chloroplast reading frames (ycf)
ORFs
transfer RNAs
ribosomal RNAs
introns

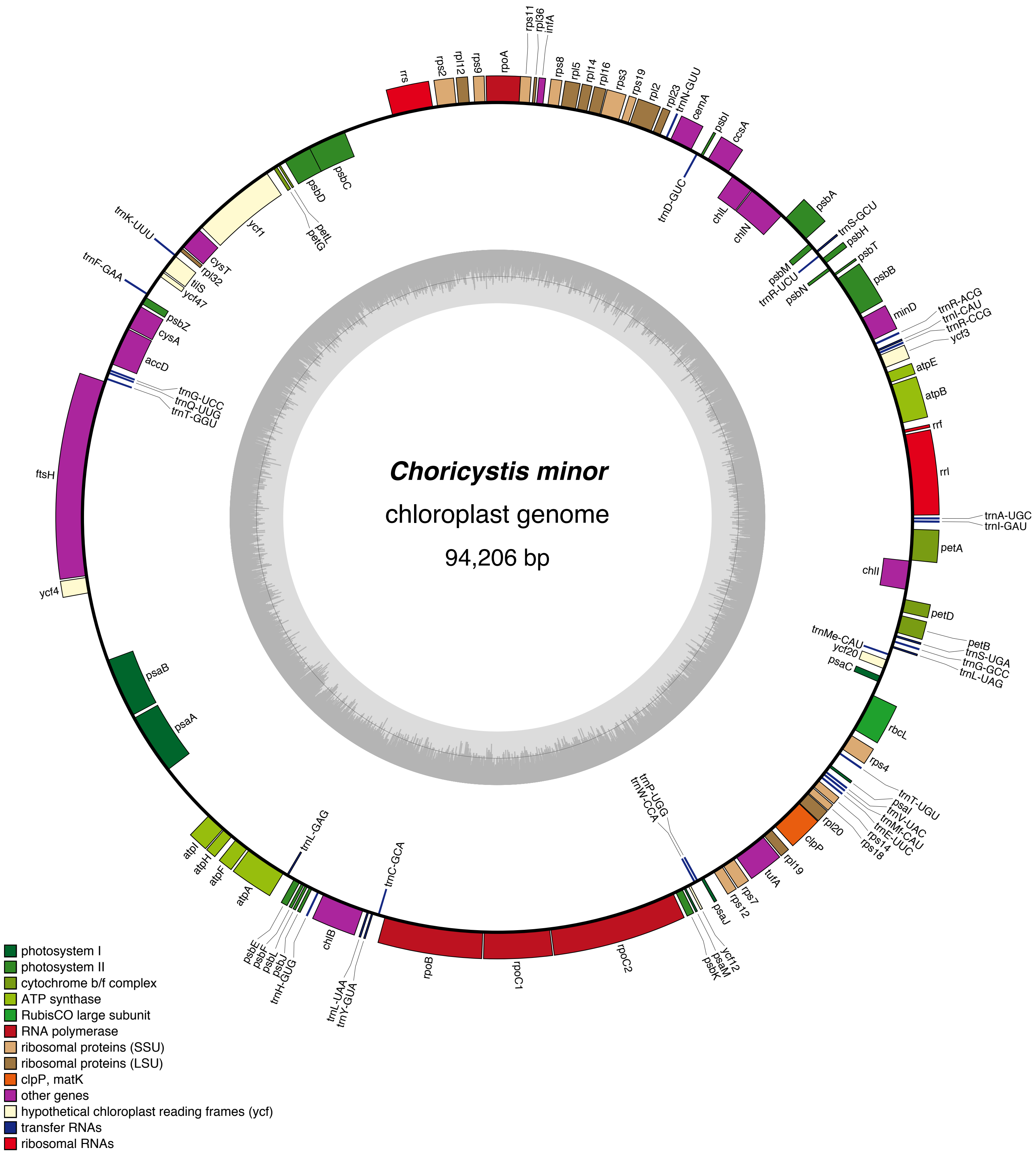

Choricystis minor
chloroplast genome
94,206 bp
rrs
rps2
rpl12
rps9
rpoA
rps11
rpl36
infA
rps8
rpl5
rpl14
rpl16
rps3
rps19
rpl2
rpl23
trnN-GUU
cemA
psbI
ccsA
trnD-GUC
chlL
chlN
psbA
trnS-GCU
psbH
psbT
psbM
trnR-UCU
psbN
psbB
minD
trnR-ACG
trnI-CAU
trnR-CCG
ycf3
atpE
atpB
rrf
rrl
trnA-UGC
trnI-GAU
petA
chlI
petD
petB
trnS-UGA
trnG-GCC
trnL-UAG
trnMe-CAU
ycf20
psaC
rbcL
rps4
trnT-UGU
psaI
trnV-UAC
trnMf-CAU
trnE-UUC
rps14
rps18
rpl20
clpP
rpl19
tufA
rps7
rps12
psaJ
ycf12
psaM
psbK
trnP-UGG
trnW-CCA
rpoC2
rpoC1
rpoB
trnC-GCA
chlB
trnL-UAA
trnY-GUA
trnL-GAG
psbE
psbF
psbL
psbJ
trnH-GUG
atpA
atpF
atpH
atpI
psaA
psaB
ycf4
ftsH
trnG-UCC
trnQ-UUG
trnT-GGU
accD
cysA
psbZ
trnF-GAA
ycf47
tilS
rpl32
cysT
trnK-UUU
ycf1
petG
petL
psbD
psbC
photosystem I
photosystem II
cytochrome b/f complex
ATP synthase
RubisCO large subunit
RNA polymerase
ribosomal proteins (SSU)
ribosomal proteins (LSU)
clpP, matK
other genes
hypothetical chloroplast reading frames (ycf)
transfer RNAs
ribosomal RNAs

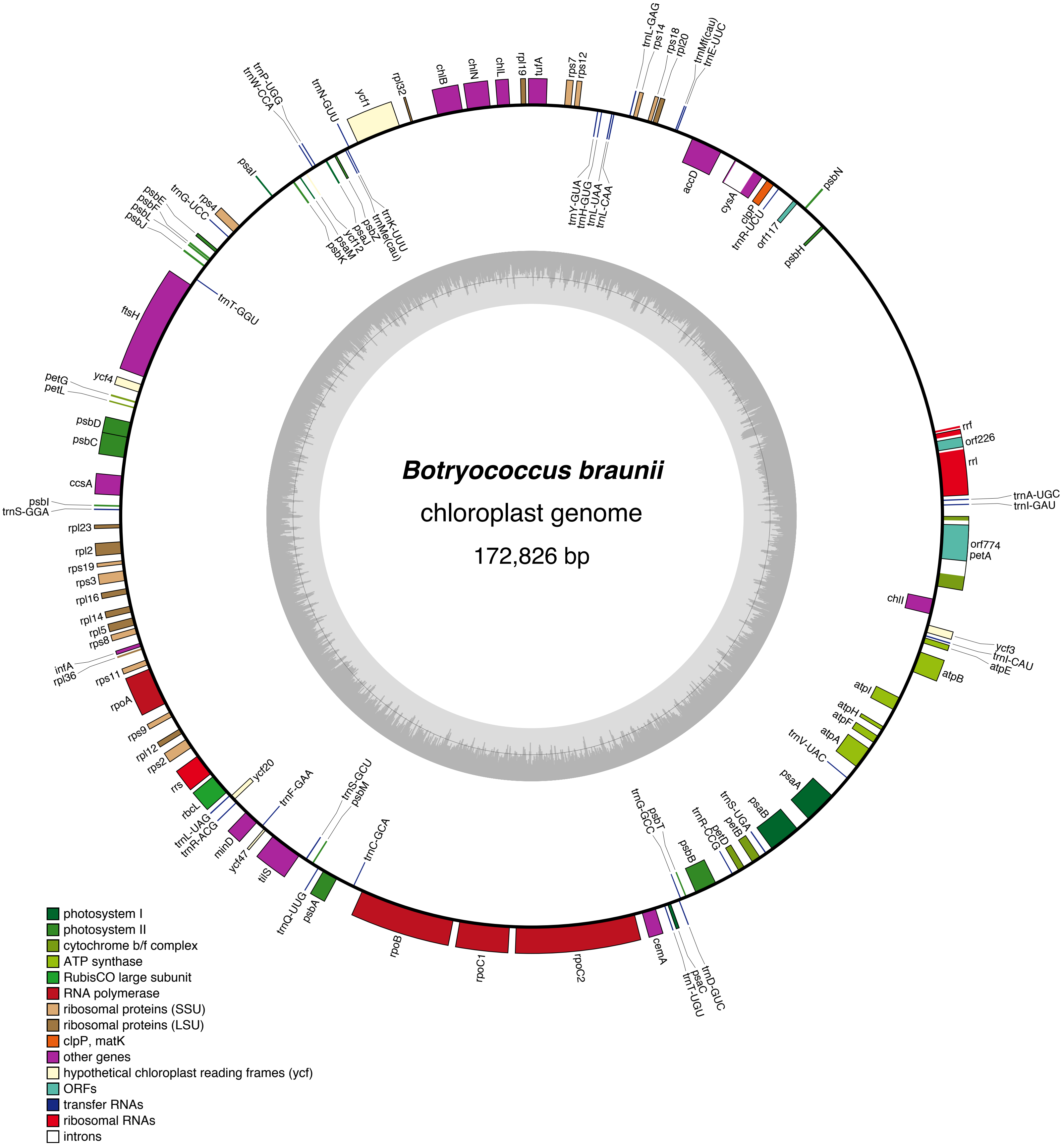
Botryococcus braunii
chloroplast genome
172,826 bp
trnL-GAG
rps14
rps18
rpl20
trnMf(cau)
trnE-UUC
rps7
rps12
tufA
rpl19
chlL
chlN
chlB
rpl32
ycf1
trnN-GUU
trnP-UGG
trnW-CCA
psaI
rps4
trnG-UCC
psbE
psbF
psbL
psbJ
trnT-GGU
ftsH
ycf4
petG
petL
psbD
psbC
ccsA
psbI
trnS-GGA
rpl23
rpl2
rps19
rps3
rpl16
rpl14
rpl5
rps8
infA
rpl36
rps11
rpoA
rps9
rpl12
rps2
rrs
rbcL
trnL-UAG
trnR-ACG
ycf20
minD
ycf47
tilS
trnF-GAA
trnS-GCU
psbM
trnQ-UUG
psbA
trnC-GCA
rpoB
rpoC1
rpoC2
cemA
trnT-UGU
psaC
trnD-GUC
psbT
trnG-GCC
psbB
trnR-CCG
petD
petB
trnS-UGA
psaB
psaA
trnV-UAC
atpA
atpF
atpH
atpI
atpB
atpE
trnI-CAU
ycf3
chlI
petA
orf774
trnI-GAU
trnA-UGC
rrl
orf226
rrf
psbH
psbN
orf117
trnR-UCU
clpP
cysA
accD
trnY-GUA
trnH-GUG
trnL-UAA
trnL-CAA
trnK-UUU
trnMe(cau)
psbZ
psaJ
ycf12
psaM
psbK
photosystem I
photosystem II
cytochrome b/f complex
ATP synthase
RubisCO large subunit
RNA polymerase
ribosomal proteins (SSU)
ribosomal proteins (LSU)
clpP, matK
other genes
hypothetical chloroplast reading frames (ycf)
ORFs
transfer RNAs
ribosomal RNAs
introns

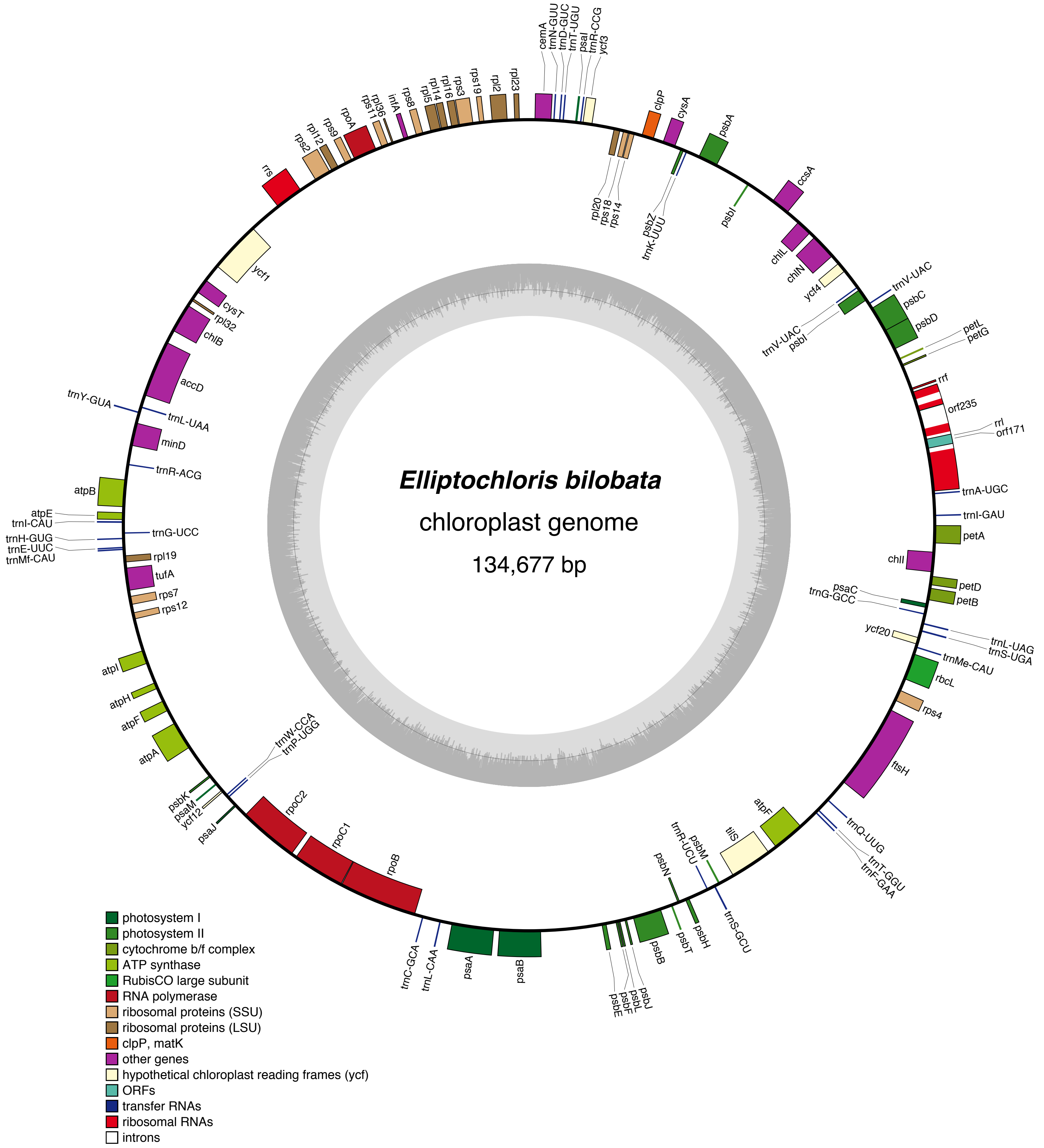

Elliptochloris bilobata
chloroplast genome
134,677 bp
cemA
trnN-GUU
trnD-GUC
trnT-UGU
psaI
trnR-CCG
ycf3
clpP
cysA
psbA
ccsA
psbI
rpl20
rps18
rps14
psbZ
trnK-UUU
chlL
chlN
ycf4
trnV-UAC
psbC
psbD
trnV-UAC
psbI
petL
petG
rrf
orf235
rrl
orf171
trnA-UGC
trnI-GAU
petA
chlI
petD
petB
psaC
trnG-GCC
trnL-UAG
trnS-UGA
ycf20
trnMe-CAU
rbcL
rps4
ftsH
trnQ-UUG
trnT-GGU
trnF-GAA
atpF
tilS
psbM
trnR-UCU
psbN
psbH
psbT
trnS-GCU
psbB
psbJ
psbL
psbF
psbE
psaB
psaA
trnL-CAA
trnC-GCA
rpoB
rpoC1
rpoC2
trnW-CCA
trnP-UGG
psbK
psaM
ycf12
psaJ
atpA
atpF
atpH
atpI
rps12
rps7
tufA
rpl19
trnG-UCC
trnH-GUG
trnE-UUC
trnMf-CAU
atpE
trnI-CAU
atpB
trnR-ACG
minD
trnY-GUA
trnL-UAA
accD
chlB
rpl32
cysT
ycf1
rrs
rps2
rpl12
rps9
rpoA
rps11
rpl36
infA
rps8
rpl5
rpl14
rpl16
rps3
rps19
rpl2
rpl23
photosystem I
photosystem II
cytochrome b/f complex
ATP synthase
RubisCO large subunit
RNA polymerase
ribosomal proteins (SSU)
ribosomal proteins (LSU)
clpP, matK
other genes
hypothetical chloroplast reading frames (ycf)
ORFs
transfer RNAs
ribosomal RNAs
introns

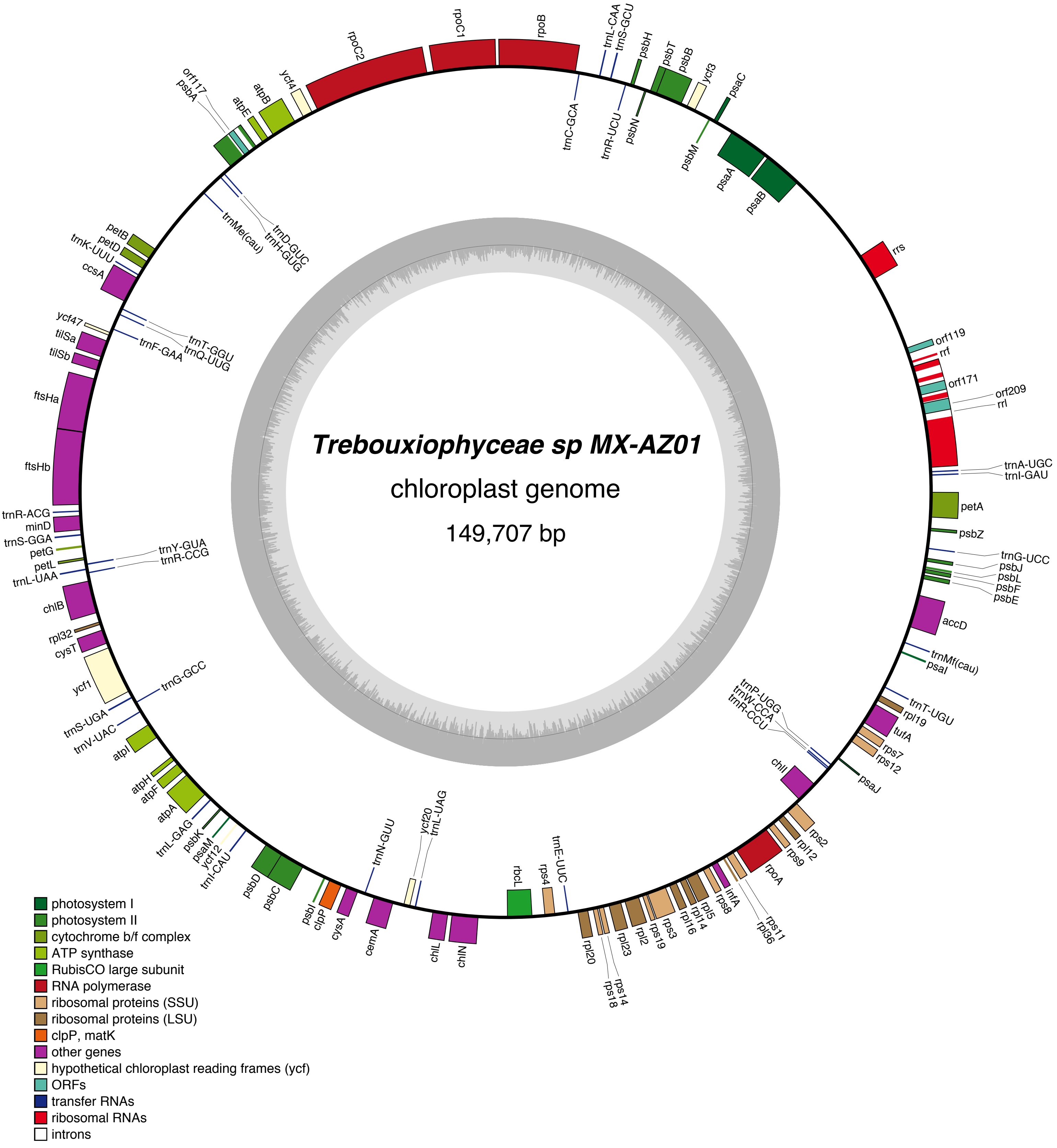

Trebouxiophyceae sp MX-AZ01
chloroplast genome
149,707 bp
rpoC2
rpoC1
rpoB
trnL-CAA
trnS-GCU
psbH
psbT
psbB
ycf3
psaC
trnC-GCA
trnR-UCU
psbN
psbM
psaA
psaB
rrs
orf119
rrf
orf171
orf209
rrl
trnA-UGC
trnI-GAU
petA
psbZ
trnG-UCC
psbJ
psbL
psbF
psbE
accD
trnMf(cau)
psaI
trnT-UGU
rpl19
tufA
rps7
rps12
psaJ
trnP-UGG
trnW-CCA
trnR-CCU
chlI
rps2
rpl12
rps9
rpoA
rps11
rpl36
infA
rps8
rpl5
rpl14
rpl16
rps3
rps19
rpl2
rpl23
rps14
rps18
rpl20
trnE-UUC
rps4
rbcL
chlN
chlL
ycf20
trnL-UAG
trnN-GUU
cemA
cysA
clpP
psbI
psbC
psbD
trnI-CAU
ycf12
psaM
psbK
trnL-GAG
atpA
atpF
atpH
atpI
trnV-UAC
trnS-UGA
trnG-GCC
ycf1
cysT
rpl32
chlB
trnL-UAA
petL
petG
trnS-GGA
minD
trnR-ACG
trnY-GUA
trnR-CCG
ftsHb
ftsHa
tilSb
tilSa
ycf47
trnF-GAA
trnT-GGU
trnQ-UUG
ccsA
trnK-UUU
petD
petB
trnMe(cau)
trnD-GUC
trnH-GUG
orf117
psbA
atpE
atpB
ycf4
photosystem I
photosystem II
cytochrome b/f complex
ATP synthase
RubisCO large subunit
RNA polymerase
ribosomal proteins (SSU)
ribosomal proteins (LSU)
clpP, matK
other genes
hypothetical chloroplast reading frames (ycf)
ORFs
transfer RNAs
ribosomal RNAs
introns

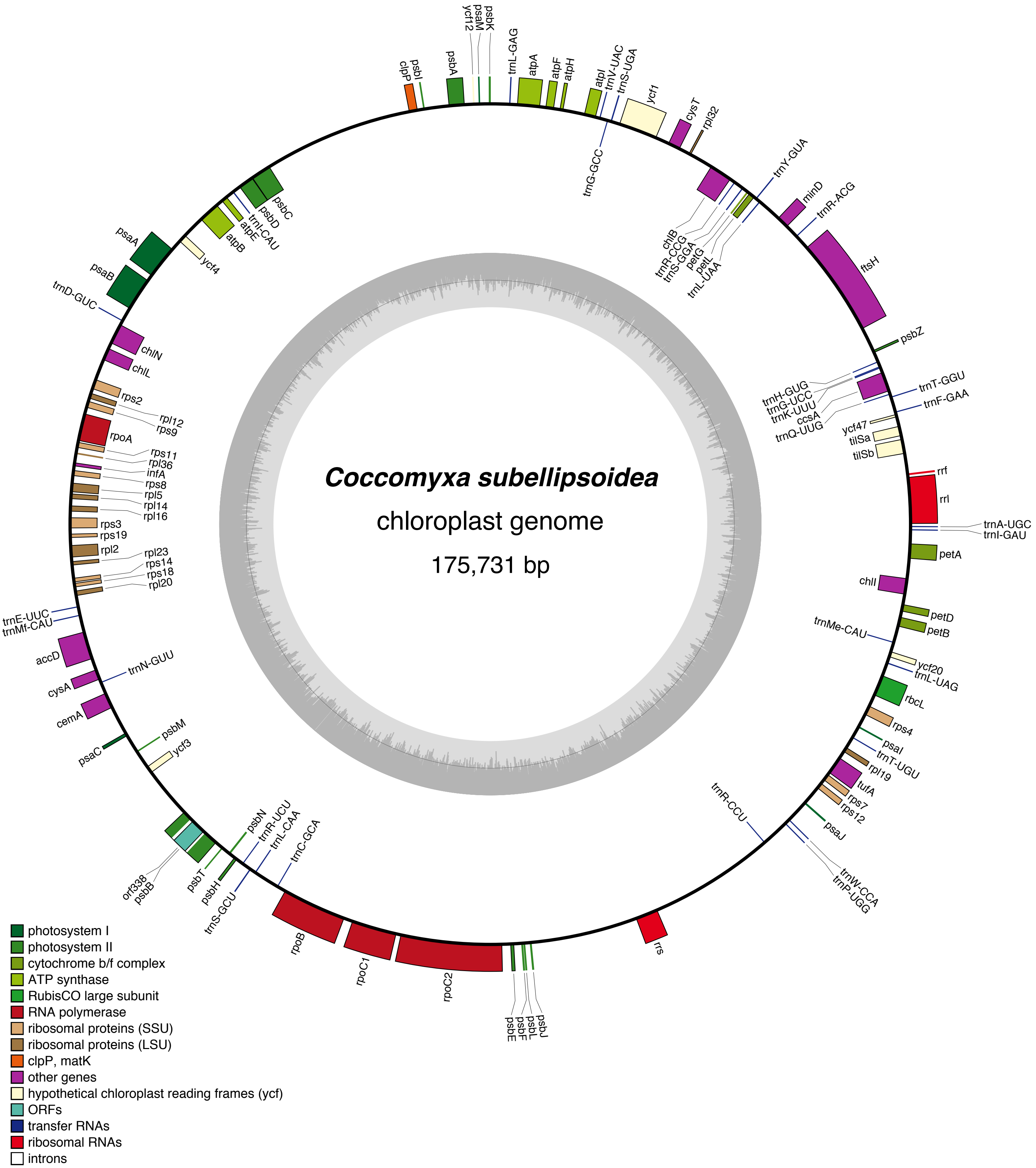

Coccomyxa subellipsoidea
chloroplast genome
175,731 bp
clpP
psbI
psbA
ycf12
psaM
psbK
trnL-GAG
atpA
atpF
atpH
atpI
trnV-UAC
trnS-UGA
ycf1
trnG-GCC
cysT
rpl32
trnY-GUA
chlB
trnR-CCG
trnS-GGA
petG
petL
trnL-UAA
minD
trnR-ACG
ftsH
psbZ
trnH-GUG
trnG-UCC
trnK-UUU
ccsA
trnQ-UUG
trnT-GGU
trnF-GAA
ycf47
tilSa
tilSb
rrf
rrl
trnA-UGC
trnI-GAU
petA
chlI
petD
petB
trnMe-CAU
ycf20
trnL-UAG
rbcL
rps4
psaI
trnT-UGU
rpl19
tufA
rps7
rps12
psaJ
trnR-CCU
trnW-CCA
trnP-UGG
rrs
psbJ
psbL
psbF
psbE
rpoC2
rpoC1
rpoB
trnC-GCA
trnL-CAA
trnR-UCU
psbN
trnS-GCU
psbH
psbT
psbB
orf338
psaC
psbM
ycf3
cemA
cysA
trnN-GUU
accD
trnMf-CAU
trnE-UUC
rpl20
rps18
rps14
rpl23
rpl2
rps19
rps3
rpl16
rpl14
rpl5
rps8
infA
rpl36
rps11
rpoA
rps9
rpl12
rps2
chlL
chlN
trnD-GUC
psaB
psaA
ycf4
atpB
atpE
trnI-CAU
psbD
psbC
photosystem I
photosystem II
cytochrome b/f complex
ATP synthase
RubisCO large subunit
RNA polymerase
ribosomal proteins (SSU)
ribosomal proteins (LSU)
clpP, matK
other genes
hypothetical chloroplast reading frames (ycf)
ORFs
transfer RNAs
ribosomal RNAs
introns

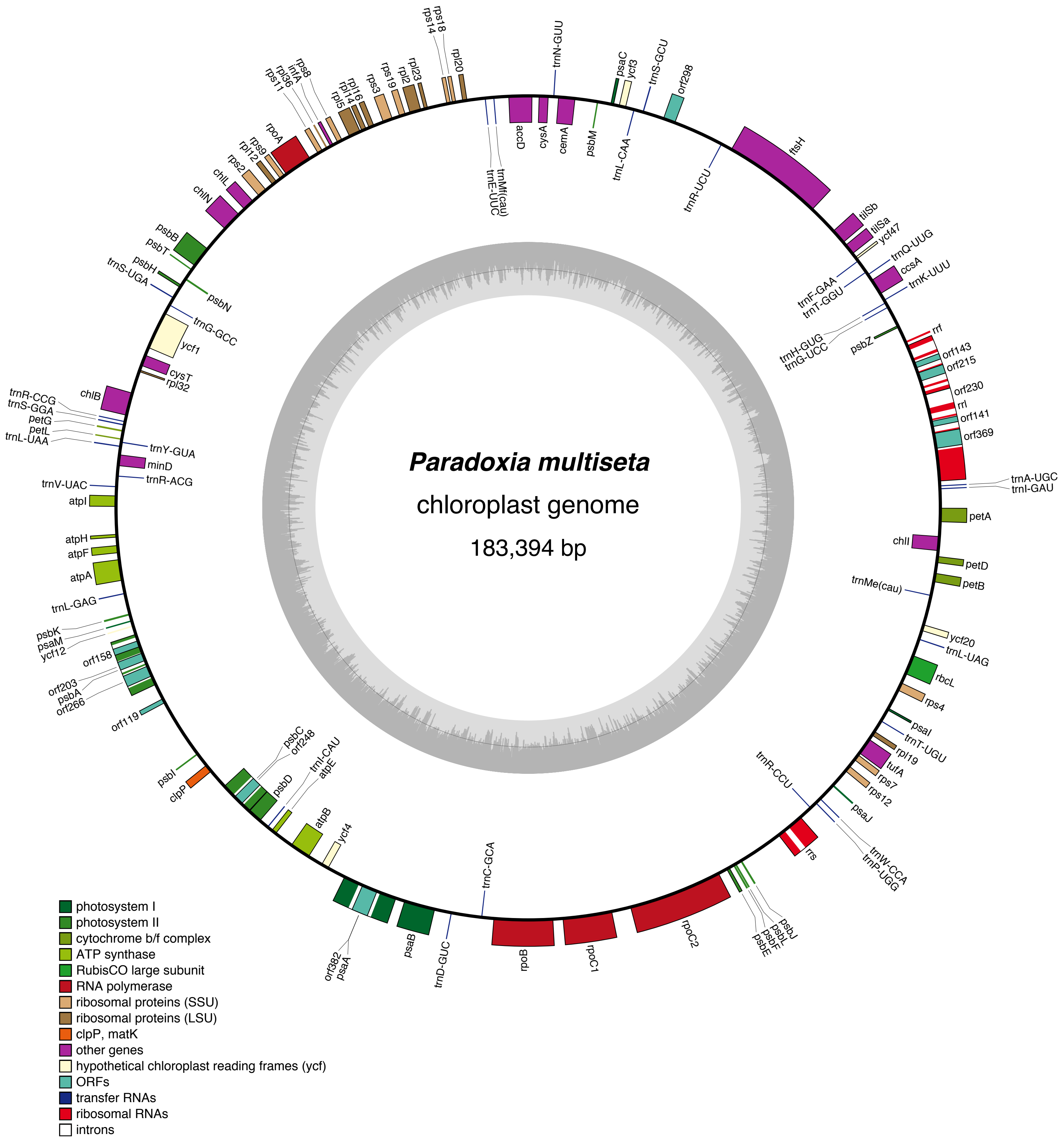

Paradoxia multiseta
chloroplast genome
183,394 bp
trnN-GUU
psaC
ycf3
trnS-GCU
orf298
accD
cysA
cemA
psbM
trnL-CAA
trnR-UCU
ftsH
trnMf(cau)
trnE-UUC
rps18
rps14
rpl20
rpl23
rpl2
rps19
rps3
rpl16
rpl14
rpl5
rps8
infA
rpl36
rps11
rpoA
rps9
rpl12
rps2
chlL
chlN
psbB
psbT
psbH
trnS-UGA
psbN
trnG-GCC
ycf1
cysT
rpl32
chlB
trnR-CCG
trnS-GGA
petG
petL
trnL-UAA
trnY-GUA
minD
trnR-ACG
trnV-UAC
atpI
atpH
atpF
atpA
trnL-GAG
psbK
psaM
ycf12
orf158
orf203
psbA
orf266
orf119
psbI
clpP
psbC
orf248
psbD
trnI-CAU
atpE
atpB
ycf4
orf382
psaA
psaB
trnD-GUC
trnC-GCA
rpoB
rpoC1
rpoC2
psbE
psbF
psbL
psbJ
rrs
trnW-CCA
trnP-UGG
psaJ
trnR-CCU
rps12
rps7
tufA
rpl19
trnT-UGU
psaI
rps4
rbcL
trnL-UAG
ycf20
trnMe(cau)
petB
petD
chlI
petA
trnA-UGC
trnI-GAU
orf369
orf141
rrl
orf230
orf215
orf143
rrf
psbZ
trnH-GUG
trnG-UCC
trnF-GAA
trnT-GGU
trnK-UUU
ccsA
trnQ-UUG
ycf47
tilSa
tilSb
photosystem I
photosystem II
cytochrome b/f complex
ATP synthase
RubisCO large subunit
RNA polymerase
ribosomal proteins (SSU)
ribosomal proteins (LSU)
clpP, matK
other genes
hypothetical chloroplast reading frames (ycf)
ORFs
transfer RNAs
ribosomal RNAs
introns
